# Construction of 11 metabolic-related lncRNAs to predict the prognosis in lung adenocarcinoma

**DOI:** 10.1186/s12920-023-01764-9

**Published:** 2023-12-18

**Authors:** Wei Jiang, Zhanyu Xu, Liuliu Huang, Fanglu Qin, Liqiang Yuan, Yu Sun, Junqi Qin, Kun Deng, Tiaozhan Zheng, Xiaomao Long, Shikang Li

**Affiliations:** 1 Department of Thoracic and Cardiovascular Surgery, The First Affiliated Hospital of Guangxi Medical University, Nanning, Guangxi Zhuang Autonomous Region 530021 China; 2 Department of Scientific Research, The First Affiliated Hospital of Guangxi Medical University, Nanning, Guangxi Zhuang Autonomous Region 530021 China; 3 Department of Cardiothoracic vascular Surgery, The People’s Hospital of Guangxi Zhuang Autonomous Region (Guangxi academy of medical sciences), Nanning, Guangxi Zhuang Autonomous Region 530021 China

**Keywords:** Long non-coding RNA, Lung adenocarcinoma, Nomogram, Risk scoring model, Immune cell infiltration

## Abstract

**Objective:**

To explore the metabolism-related lncRNAs in the tumorigenesis of lung adenocarcinoma.

**Methods:**

The transcriptome data and clinical information about lung adenocarcinoma patients were acquired in TCGA (The Cancer Genome Atlas). Metabolism-related genes were from the GSEA (Gene Set Enrichment Analysis) database. Through differential expression analysis and Pearson correlation analysis, lncRNAs about lung adenocarcinoma metabolism were identified. The samples were separated into the training and validation sets in the proportion of 2:1. The prognostic lncRNAs were determined by univariate Cox regression analysis and LASSO (Least absolute shrinkage and selection operator) regression. A risk model was built using Multivariate Cox regression analysis, evaluated by the internal validation data. The model prediction ability was assessed by subgroup analysis. The Nomogram was constructed by combining clinical indicators with independent prognostic significance and risk scores. C-index, calibration curve, DCA (Decision Curve Analysis) clinical decision and ROC (Receiver Operating Characteristic Curve) curves were obtained to assess the prediction ability of the model. Based on the CIBERSORT analysis, the correlation between lncRNAs and tumor infiltrating lymphocytes was obtained.

**Results:**

From 497 lung adenocarcinoma and 54 paracancerous samples, 233 metabolic-related and 11 prognostic-related lncRNAs were further screened. According to the findings of the survival study, the low-risk group had a greater OS (Overall survival) than the high-risk group. ROC analysis indicated AUC (Area Under Curve) value was 0.726. Then, a nomogram with T, N stage and risk ratings was developed according to COX regression analysis. The C-index was 0.743, and the AUC values of 3- and 5-year survival were 0.741 and 0.775, respectively. The above results suggested the nomogram had a good prediction ability. The results based on the CIBERSORT algorithm demonstrated the lncRNAs used to construct the model had a strong correlation with the polarization of immune cells.

**Conclusions:**

The study identified 11 metabolic-related lncRNAs for lung adenocarcinoma prognosis, on which basis a prognostic risk scoring model was created. This model may have a good predictive potential for lung adenocarcinoma.

**Supplementary Information:**

The online version contains supplementary material available at 10.1186/s12920-023-01764-9.

## Introduction

The incidence of lung cancer has been increasing these years, and its mortality even ranks top in various diseases [1–3]. Therefore, early diagnosis and accurately treatments are essential to improve the prognosis of patients. As high-throughput sequencing technology developing, many studies have developed various disease prognosis models based on high-throughput sequencing data [4, 5]. However, seldom studies have connected the clinical information with tissue sequencing data to establish a prediction model for patients with lung adenocarcinoma [6]. Our study plans to create a prediction model of lung adenocarcinoma prognosis based on metabolism-related lncRNAs and clinical information, which might be a valuable addition to the existing prognostic assessment of lung adenocarcinoma.

More and more studies pay attention to the role of metabolism in tumor prognosis. Chen et al. [7] found that TBC1D8 (TBC1 Domain Family Member 8) did not depend on the activity of GAP [4] in vitro and in vivo to promote OVCA(Ovarian cancer-associated gene) and aerobic glycolysis, TBC1D8 drove OVCA and metabolic reprogramming, and it can be a prognostic biomarker for OVCA patients. Previous studies have found that ZFP91 (Zinc finger protein 91) can be a tumor suppressor for liver cancer occurrence and metabolic reprogramming, which can be a potential new prognostic biomarker and HCC (Hepatocellular Carcinoma) treatment target [8]. Cancer-associated fibroblasts (CAFs) have been found to enhance glutamine metabolism in lung adenocarcinoma. Caf-specific long non-coding RNA LINC01614 was packaged by CAF-derived exosomes and mediated the enhanced glutamine uptake in LUAD cells. Finally, it was found that targeting caf specific lncrnas for treatment could inhibit glutamine utilization and LUAD cancer progression [9].

Long non-coding RNA (lncRNA) is kind of RNA with over 200 nucleotides, whose function is mainly achieved by regulating the expression of genes and interacting with specific proteins. A large amount of evidence has shown that lncRNAs are related to multiple biological activities, such like tumor cell proliferation and differentiation and so on. For example, LncRNA HOTAIR affects the growth, migration, invasion and apoptosis of breast cancer cells through the miR-20a-5p/HMGA2 axis [10]. The expression and function of lncRNA in lung adenocarcinoma has been gradually unveiled in recent years. According to research by Pan et al. [11],the lncRNA JPX is up-regulated in lung cancer tissues with metastasis, strongly correlated with the size and stage of the tumor, suggesting that it might be a possible target for targeted therapy. Peng et al. [12] proved that LINC00312 can induce lung cancer cell migration, invasion and angiogenesis, and affect the prognosis by directly binding the transcription factor YBX1 (Y-box binding protein 1).

In addition, metabolism-related lncRNAs have an prognostic impact on the patients with lung adenocarcinoma [13]. Qianet al. [14]revealed that lncRNA-AC020978 is up-regulated in non-small cell lung cancer, which is closely related to more advanced TNM staging and unsatisfying clinical results, and it can be used as a prognosis predictor. Functional analysis showed that lncRNA-AC020978 plays a key role in promoting cell growth and metabolic reprogramming. Yang et al. [15] first demonstrated that lnc-IGFBP4-1 was markedly increased in lung cancer, and played an active role in cell metastasis and proliferation by reprogramming the energy metabolism of tumor cells. Studies have reported that, LINC01703 promotes the malignant characteristics of non-small cell lung cancer by upregulating MACC1 through microrNA-605-3p [16].

In summary, metabolism-related lncRNAs may play important roles in lung adenocarcinoma and are potential therapeutic targets for the diagnosis, treatment and prognosis of lung adenocarcinoma. Therefore, the study intends to screen prognostic factors based on metabolism-related lncRNAs and create a prognostic risk assessment model for lung adenocarcinoma to assess the prognosis of lung adenocarcinoma.

## Methods

### Data and processing

Lung adenocarcinoma lncRNA and mRNA transcriptome data, corresponding annotation files and clinical information were downloaded in the Cancer Genome Atlas Database (TCGA). Metabolism-related genes were identified in Gene Set Enrichment Analysis database (GSEA, https://www.gsea-msigdb.org/gsea/index.jsp). To find metabolic-related genes and lncRNAs expressed differently in cancer and normal samples, we utilized |log_2_FC|>1 and false discovery rate (FDR) < 0.05 as criteria. The relationship between metabolism-related genes and lncRNAs was measured by Pearson correlation analysis coefficient (R^2^). Similarly, lncRNAs with R^2^ > 0.5 and *P* < 0.05 were connected with lung adenocarcinoma metabolism.

### Construction of the risk scoring model

Lung adenocarcinoma patients were separated into the training and the validation sets in the proportion of 2:1, then metabolic-related lncRNAs were analyzed by univariate Cox regression analysis, and *P* < 0.05 was set as the standard to identify prognostic-related lncRNAs. LASSO regression analysis was applied to determine the key lncRNAs closely related to OS, incorporate the selected key lncRNAs into the multivariate Cox regression analysis to calculate their risk coefficients, and construct a risk scoring model including the coefficients and the expression level of lncRNAs to obtain all risk scores. Patients were split into high-risk and low-risk groups based on a cutoff point determined by the median of the risk score. The prognosis was compared using the survival curve. The AUC value in ROC analysis evaluated the prediction effect of the model. The above analysis was verified through the verification set. Finally, the model was further evaluated by dividing the patients into different subgroups according to gender, age, tumor and TNM (tumor node metastasis classification) stages.

### The creation and assessment of the nomogram

Univariate and multivariate Cox regression analyses were performed on risk score and clinical factors to screen independent prognostic factors. We built a nomogram on the basis of multivariate Cox regression including risk scores. The 3-year and 5-year OS analyses for patients were predicted according to the nomogram. At the same time, the consistency index, ROC and calibration curves were calculated to assess the effect of the nomogram. All the findings were confirmed in the verification set to ensure the stability of the results.

### Analysis of the correlation between prognostic lncRNAs and tumor immunity

First, CIBERSORT [17] analysis was applied to determine the infiltration levels of 22 immune cells in lung adenocarcinoma patients, and then the correlation of 11 prognostic metabolism-related lncRNAs with immune cell infiltration was determined on the basis of correlation analysis, and the impact of identified lncRNAs on the progression of lung adenocarcinoma was further explored.

## Results

### Differential expression analysis and correlation analysis to obtain metabolism-related lncRNAs

**Fig. 1:**
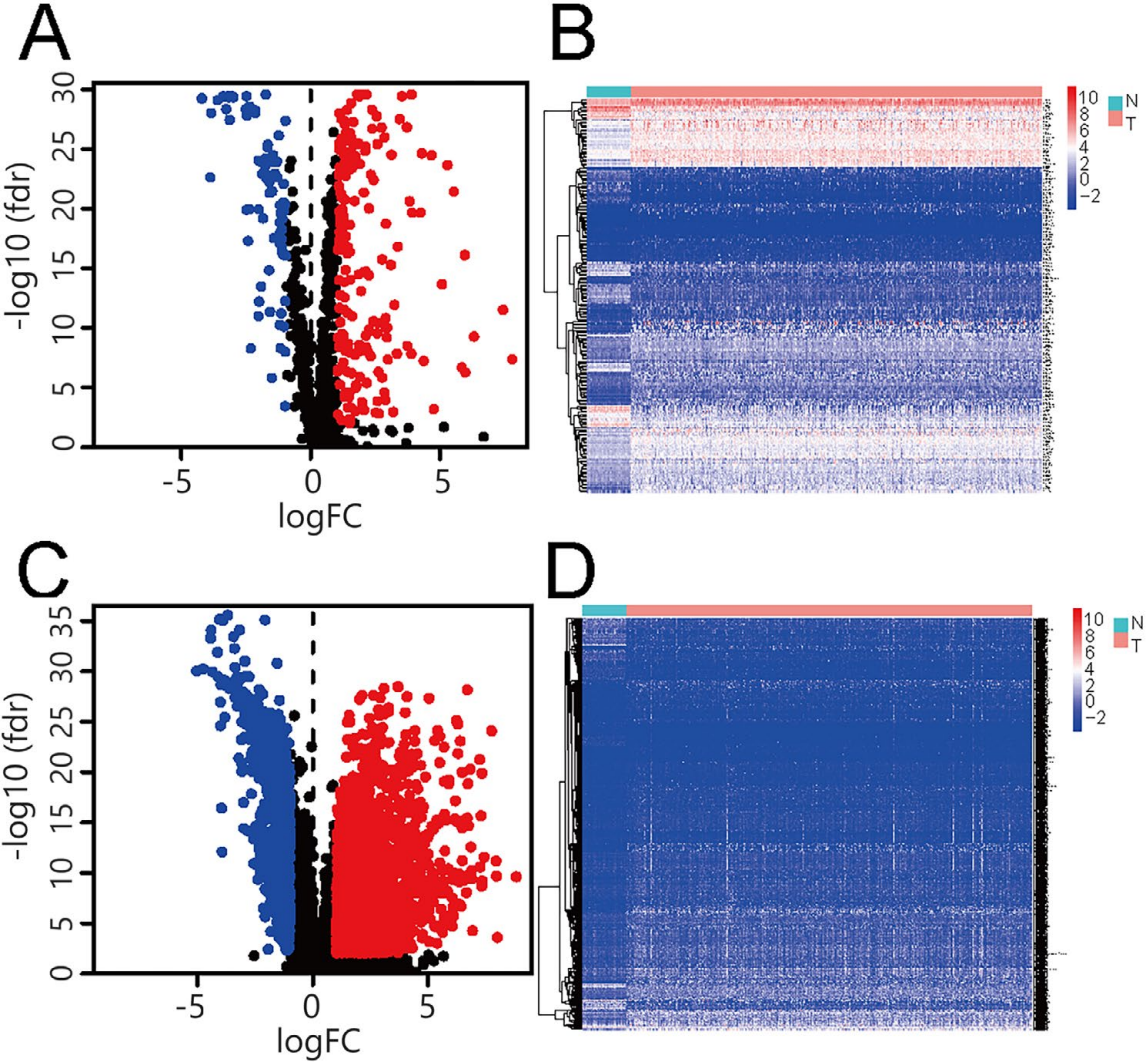
The volcano map reveals the differentially expressed metabolism-related genes and lncRNAs in lung adenocarcinoma and adjacent samples. Red represents a significant up-regulation of lncRNAs and metabolism-related genes, blue refers to a down-regulation of lncRNAs and metabolism-related genes, and black represents no significant difference in gene expression. (**A**) Volcano map of differentially expressed metabolism-related genes. (**B**) Heat maps of genes with differential expression. (**C**) Volcano map of differentially expressed metabolism-related lncRNAs. (**D**) Heat maps of lncRNAs with differential expression

497 tumor and 54 paracancerous samples were acquired from TCGA, and metabolism-related genes from GSEA. Between lung adenocarcinoma tumors and paracancerous tissues, 78 down-regulated and 175 up-regulated differentially expressed metabolism-related genes were identified (Fig. 1A, Supplementary materials Table 1), and their heat maps were also analyzed (Fig. 1B). In addition, we identified 650 down-regulated and 2345 up-regulated differentially expressed lncRNAs (Fig. 1C, Supplementary materials Table 2), and their heat maps were also analyzed (Fig. 1D). By Pearson correlation analysis, we obtained 233 metabolic-related lncRNAs (|R^2^|>0.5, *P* < 0.05, Supplementary materials Table 3).

### The creation of prognostic risk scoring model

**Fig. 2:**
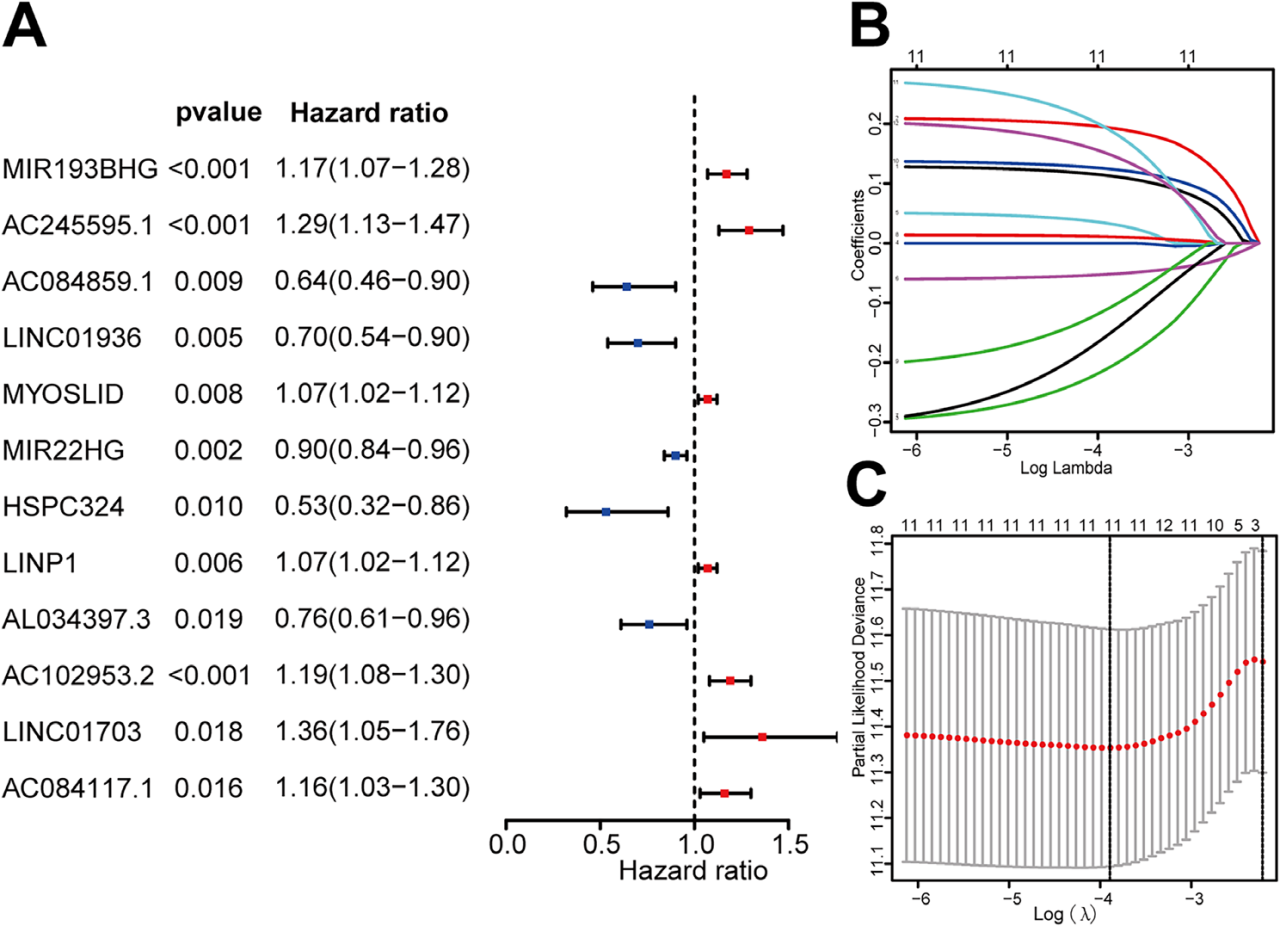
The identification of metabolic-related lncRNAs with clinical value for lung adenocarcinoma prognosis. (**A**) Twelve metabolic-related lncRNAs substantially correlate with OS. (**B**) LASSO regression model parameters. (**C**) LASSO coefficient spectrum of lncRNAs with predictive significance

**Fig. 3:**
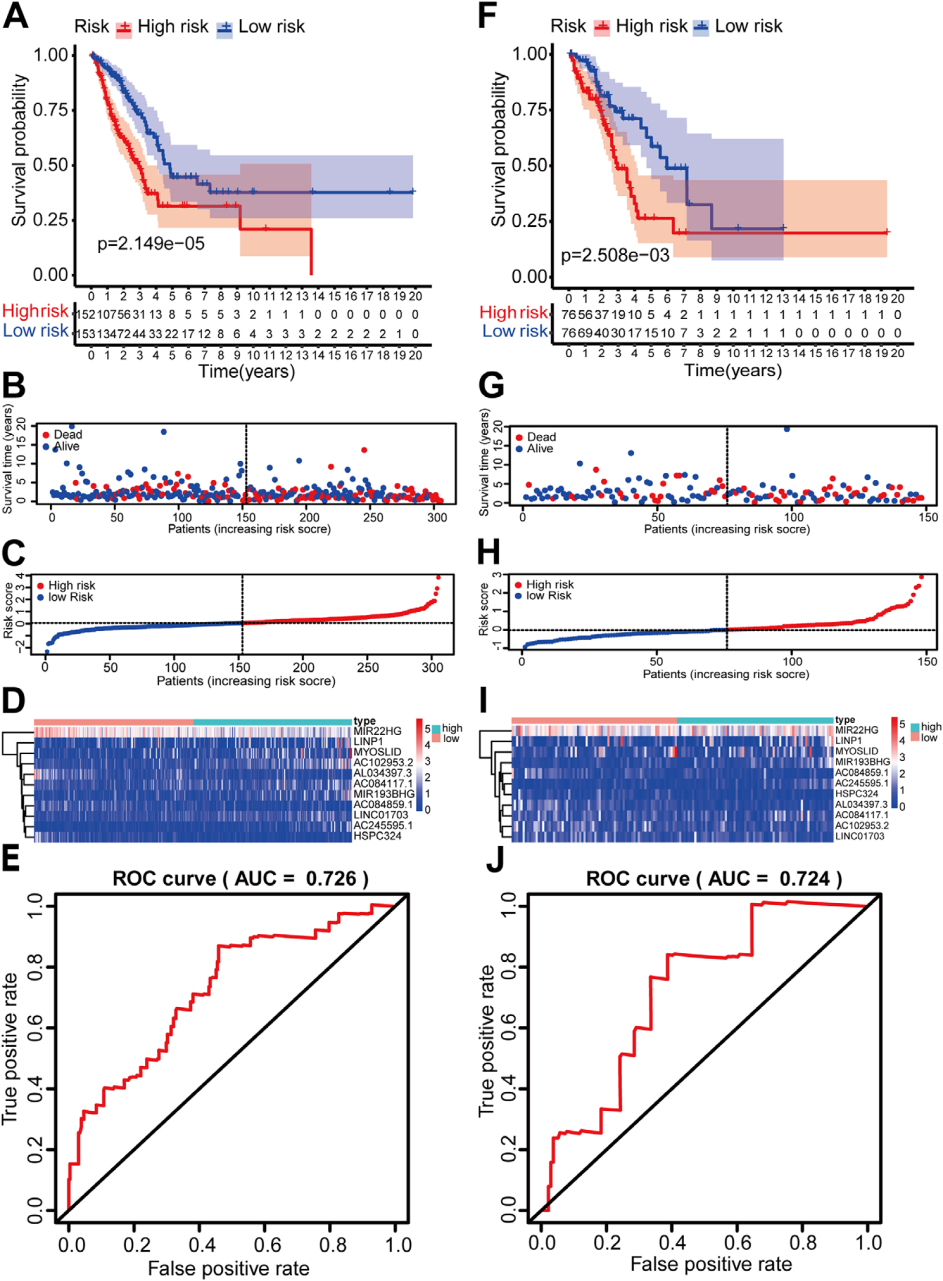
The risk scoring model construction. (**A, F**) Kaplan-Meier analysis on lung adenocarcinoma patients in the test and validation sets showed that the prognosis of the low-risk group was better. (**B, G**) The survival rate and survival status of patients with lung adenocarcinoma. (**C, H**) The distribution of 11 lncRNA risk scores for lung adenocarcinoma patients. (**D, I**) Heat maps of 11 lncRNAs in the high-risk and low-risk groups. (**E, J**) ROC curve analysis of lung adenocarcinoma patients

In a 2:1 ratio, the samples were split into test and validation sets. In order to investigate the prognostic importance of metabolism-related lncRNAs, patients with a follow-up period of more than 30 days were taken into account. 18 prognostic-related lncRNAs were obtained (*P* < 0.05, Fig. 2A). Through LASSO regression analysis, we further identified 11 core lncRNAs closely associated with prognosis (Fig. 2B, C, Supplementary materials Table 4). A model for risk score was created using multivariate Cox regression. The median of the risk score served as the dividing standard between the high-risk and low-risk groups of patients. The survival curve demonstrated the OS was much better in the low-risk group (*P* < 0.001, Fig. 3A). The mortality and coefficient of the low-risk group were lower than those of the high-risk group, according to the risk curve and scatter plot (Fig. 3B, C). The heat map showed the levels of 11 lncRNAs between the two groups (Fig. 3D). The AUC obtained in ROC analysis was 0.726 (Fig. 3E). Similar outcomes in the validation set were attained (Fig. 3F-J).

### Subgroup analysis

**Fig. 4:**
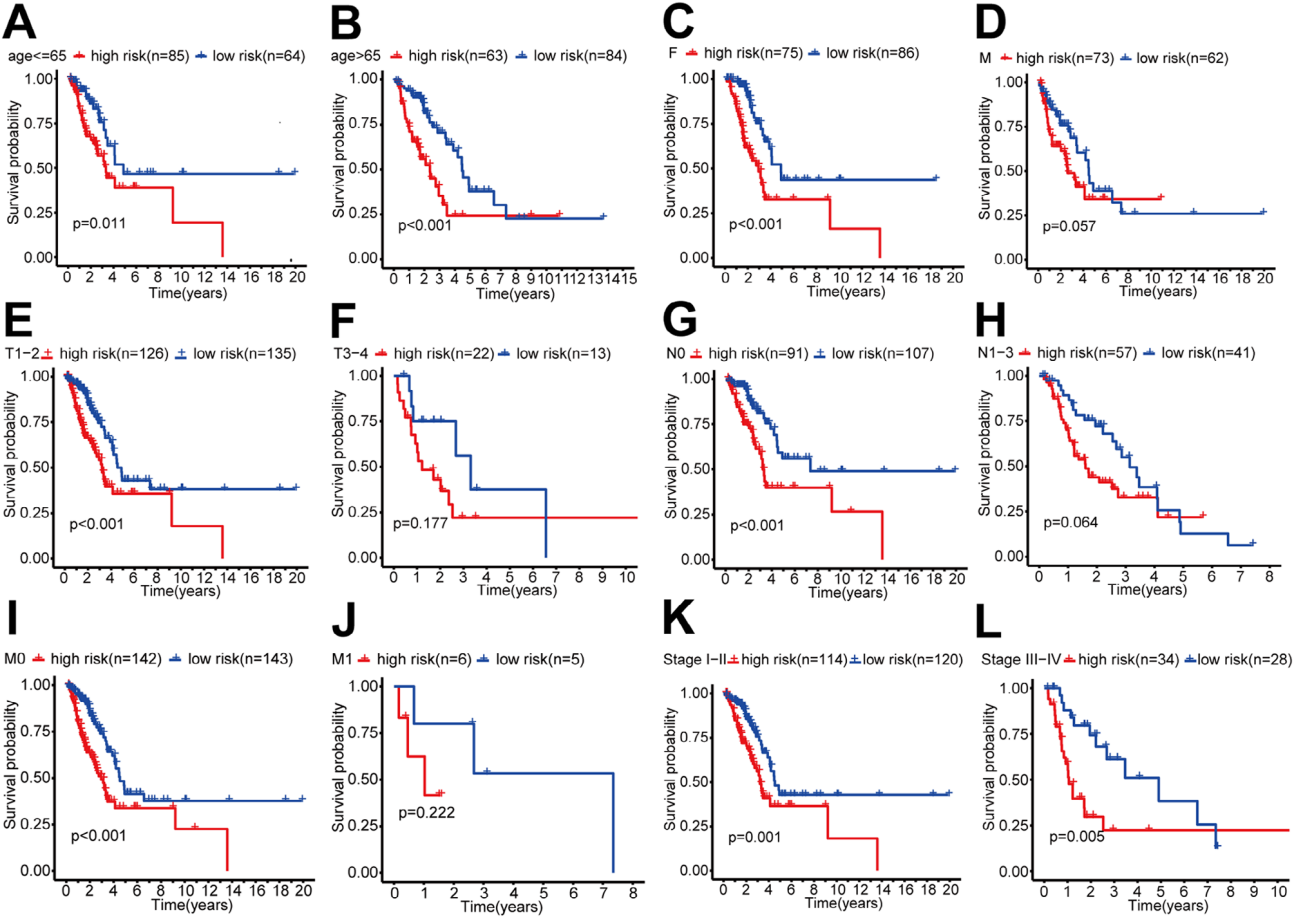
Survival curves of lung adenocarcinomapatients in each clinical subgroup

Results in subgroups indicated the risk score model had the ability to differentiate ages that greater than 65 years old, younger than 65 years old, female, T1-2, stage I-II, stage III-IV, N0 and M0 (*P* < 0.05). Because the sample of male, N1-3, T3-4, and M1 subgroups was relatively low, the survival curve indicated a possible identification tendency, even if there was no statistically significant difference (Fig. 4).

### Screening of independent prognostic factors

**Fig. 5:**
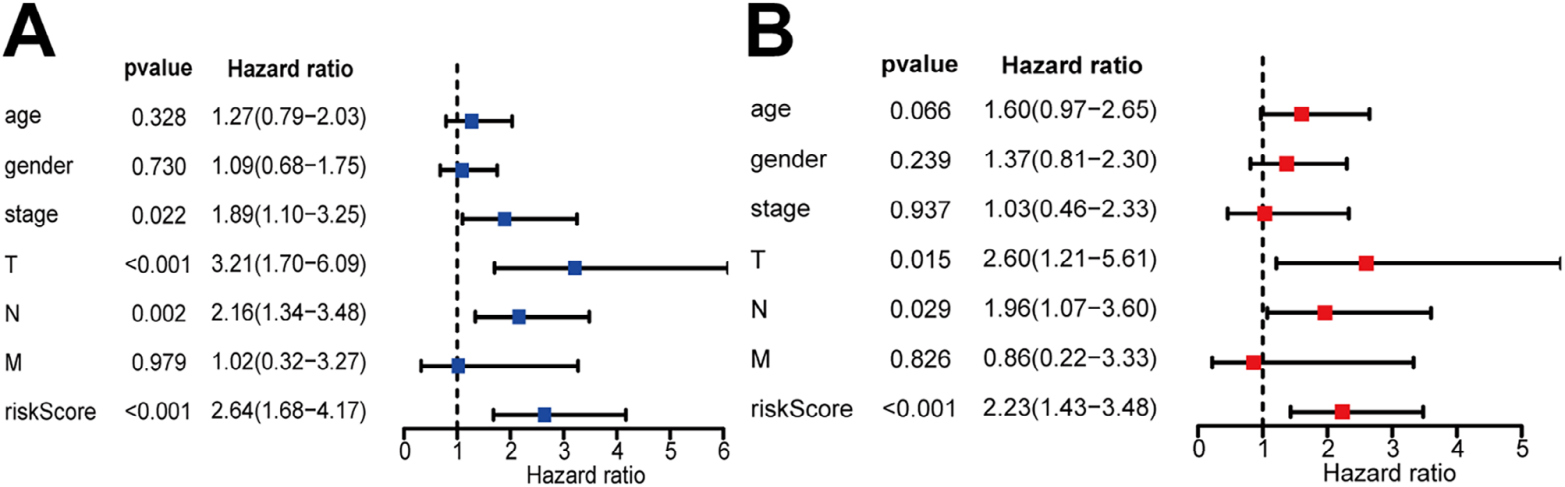
The prognostic value evaluation of clinicopathological characteristics and risk scores. (**A**) Univariate Cox regression analysis on lung adenocarcinoma patients. (**B**) Multivariate Cox regression analysis on lung adenocarcinoma patients

Results of univariate and multivariate Cox regression analysis demonstrated T, N stage and 11 lncRNAs risk scores were prognostic factors for patients with lung adenocarcinoma (Fig. 5A, B). The prognostic nomogram was then constructed by these factors.

### The prognostic nomogram construction

**Fig. 6:**
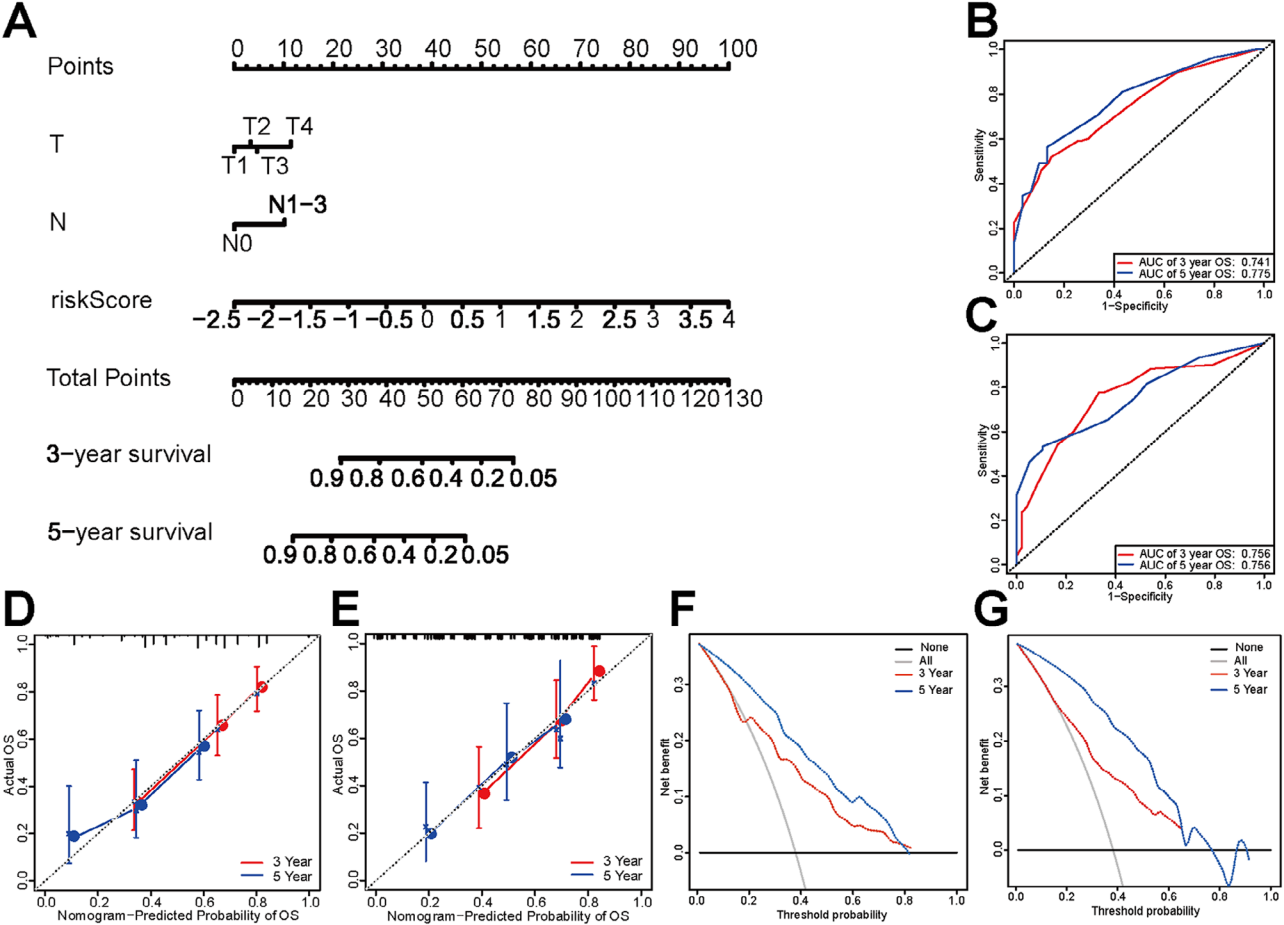
The prognostic nomogram construction and assessment. (**A**) The nomogram of the 3- and 5-year OS of the training and validation sets. (**B, C**) ROC curve of the test set and the validation set. (**D, E**) Calibration nomogram of the test set and the verification set. (**F, G**) DCA clinical decision curve of the test set and the validation set

According to the above findings, the nomogram was constructed, including T, N stage and risk scores (Fig. 6A). The C index of the nomogram was 0.743, the AUC values of the 3- and 5-year OS were 0.741 and 0.775, respectively (Fig. 6B). The calibration curve (Fig. 6D), the clinical decision curve DCA (Fig. 6F) all indicated the model possessed a good prediction ability and can bring benefits to patients. The above results were all verified in the internal verification set so that the stability of the results can be verified (Fig. 6C, E, G).

### Correlation between prognostic lncRNAs and tumor immunity

**Fig. 7:**
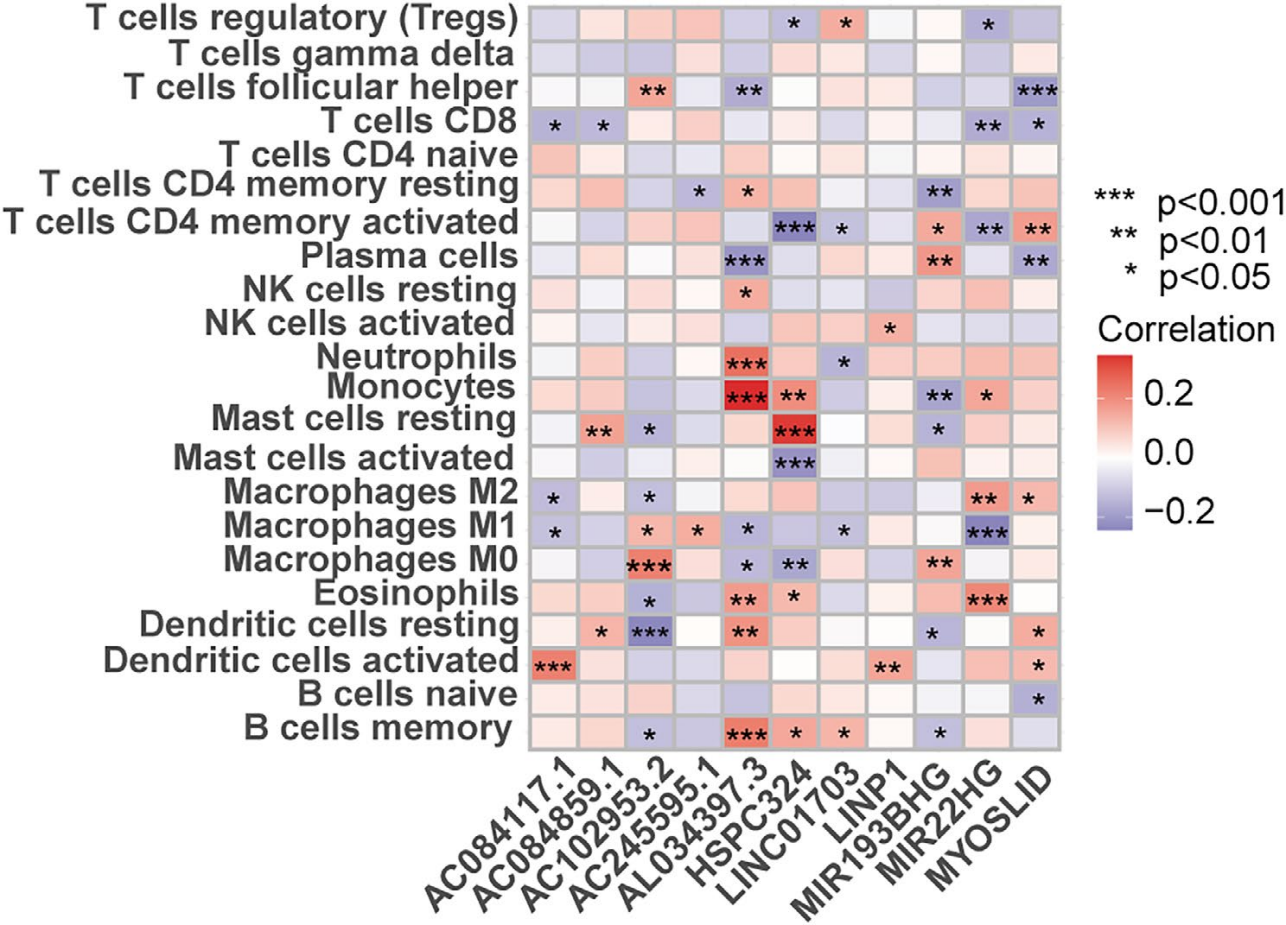
Correlation between the proportion of immune cells based on the cibersort algorithm and11 lncRNAs used expressions in the model

The CIBERSORT analysis clarified the infiltration level of 22 immune cells in patients with lung adenocarcinoma. The results showed that lncRNAs used to construct the model had a strong correlation with the polarization of monocytes, resting mast cells and M1 macrophages (Fig. 7), suggesting that these lncRNAs may regulate the growth and progression of tumors by affecting the microenvironment of tumor immune infiltration.

## Discussion

In the past decade, molecularly targeted drugs and immune checkpoint inhibitors has improved the survival of patients in advanced lung cancer [18, 19]. However, this disease remains the primary factor in cancer-related fatalities globally. In the last few years, many studies have shown that expression changes of metabolism-related genes are markedly associated with the occurrence and prognosis of lung adenocarcinoma [20, 21]. Studies have shown that [22]epidermal growth factor receptor (EGFR) binds to stearoyl-CoA desaturase 1 (SCD1) and phosphorylates it at Y55, thereby increasing the level of monounsaturated fatty acids (MUFA) and promoting the growth of lung cancer. Phosphorylated SCD1 Y55 can be the prognostic biomarker for patients with low survival rate. Given that tumor metabolism is markedly connected with lung adenocarcinoma [23],Finding trustworthy metabolic-related prognostic markers can aid in early risk assessment and wise treatment choices for lung cancer.

LncRNAs have a variety of strategies to control the metabolic process. In order to control the expression of associated genes, lncRNAs particularly adsorb metabolism-related miRNAs like molecular sponges. In the investigation on the part metabolism-related lncRNAs in lung cancer [24]. discovered NEAT1 (nuclear paraspeckle assembly transcript 1) was crucial to the development and spread of LUAD (Lung adenocarcinoma), and it may act as a ceRNA to regulate miR-193a-3p. However, the prognostic value of metabolism-related lncRNAs in lung adenocarcinoma is not assessed comprehensively before.

Herein, lung adenocarcinoma transcriptome data and clinical information were acquired from the TCGA. Bioinformatics analysis, 11 metabolic-related lncRNAs with prognostic value were identified, on which basis we created a risk score model. Subsequently, to predict the 3- and 5-year OS of patients, we developed a nomogram based on risk scores and clinical factors (T and N stages). The C-index, calibration and ROC curve showed that the model may have good predictive potential for lung adenocarcinoma.

We screened 11 prognostic-related lncRNAs, namely: MIR193BHG, AC245595.1, AC084859.1, MYOSLID, MIR22HG, HSPC324, LINP1, AL034397.3, AC102953.2, LINC01703, and AC084117.1. Previous research revealed that the chosen lncRNAs were crucial in the emergence and progression of lung cancer. [13, 25]. Xu et al. [26] found that MIR22HG (MIR22 host gene) has a tumor suppressor effect in colorectal cancer. By competing with SMAD2 (Recombinant Mothers Against Decapentaplegic Homolog 2) and modifying the TGF pathway’s activity, MIR22HG was able to exhibit its tumor suppressor effect. The epithelial-mesenchymal transition of CRC was aided by the decreased MIR22HG.Zhang et al. [27] exhibited TGF-β1 blocked lncRNA transcription in non-homologous end joining pathway 1 (LINP1) in a SMAD4-dependent way, and LINP1 prevented lung cancer cells from engaging in EMT (epithelial–mesenchymal transition), hence regulating cancer cell motility, invasion, and stemness.

In order to further reveal the role of 11 lncRNAs in lung adenocarcinoma, we investigated the relation between 11 prognostic-related lncRNAs in the expression matrix of lung adenocarcinoma and 22 kinds of immune cell infiltration by CIBERSORT algorithm. The results showed that these 11 lncRNAs showed a strong correlation with monocytes cells, resting mast cells, and polarized M1 macrophages, suggesting that these lncRNAs may affect the tumor microenvironment through immune infiltration, thereby affecting the overall outcome of lung adenocarcinoma. The specific mechanism remains to be further studied. Studies have shown that tumor-infiltrating immune cells are highly correlated with the prognosis of hepatocellular carcinoma (HCC) and the identification of immunotherapy targets. Activated mast cells, monocytes, and plasma cells were reduced in HCC compared to healthy liver, whereas resting mast cells, total B cells and naive B cells, CD4 + memory resting T cells, and CD8 + T cells were increased. Stronger total immune cell infiltration in HCC was associated with total B cells, memory B cells, follicular helper T cells, and M1-type macrophages, whereas weak infiltration was associated with resting NK cells, neutrophils, and resting mast cells. The frequency of mast cells was reduced in human HCC tumor tissues compared with adjacent tissues. The results of this study are consistent with the text section and further illustrate the relationship between tumor-infiltrating immune cells and tumor prognosis [28].

The study indicated that 11 lncRNAs were essential for metabolic regulation and the pathogenesis of lung adenocarcinoma. However, the specific mechanism of 11 metabolic-related lncRNAs in the progression of lung adenocarcinoma still need to be further elucidated in in vitro experiment. Besides, the study still has certain limitations. Since it is a retrospective study based on public database, there is a lack of information on individual treatment. In addition, the lung adenocarcinoma patient cohort is quite small, which has an impact on the model’s ability to predict outcomes in some subgroups. To validate our findings, more in vivo or in vitro investigations and prospective clinical trials are required.

## Conclusions

In summary, a lung adenocarcinoma prognostic risk score model including 11 metabolism-related lncRNAs was constructed, and the predictive stability of the model was fully verified in multiple datasets. The model can provide certain guidance for the prognosis of lung adenocarcinoma patients.

### Electronic supplementary material

Below is the link to the electronic supplementary material.

Supplementary Material 1

Supplementary Material 2

## Data Availability

The original contributions presented in the study are publicly available. This data can be found here: https://portal.gdc.cancer.gov/projects/TCGA-LUAD.

## Abbreviations

LncRNA: Long non-coding RNA
TCGA: The Cancer Genome Atlas Database
GSEA: The Gene Set Enrichment Analysis database
LASSO: Least Absolute Shrinkage and Selection Operator
DCA: Decision Curve Analysis
OS: Overall survival
ROC: Receiver operating characteristic curve
FDR: False discovery rate
EGFR: Epidermal growth factor receptor
SCD1: Stearoyl-CoA desaturase 1
MUFA: Monounsaturated fatty acids
LINP1: LncRNAs in non-homologous end joining pathway 1
