# Supplementary material for: Construction of 11 metabolic-related lncRNAs to predict the prognosis in lung adenocarcinoma
Source: BMC Med Genomics. 2023 Dec 18;16:330. doi: 10.1186/s12920-023-01764-9 (PMC10726503; doi:10.1186/s12920-023-01764-9)
Supplement: Supplementary file 1 — Supplementary Material 1 [file 12920_2023_1764_MOESM1_ESM.pdf]

Supplementary materials Table 1

gene	conMean	treatMean	logFC	pValue	fdr
PRIM1	1.981292	4.173344	1.074762	8.23E-13	2.10E-12
DAO	0.075084	0.01412	-2.41074	1.85E-21	1.09E-20
ADH1A	0.252589	0.074431	-1.76281	3.17E-24	2.48E-23
GGCT	10.07999	36.31863	1.849216	4.76E-32	3.65E-30
GLDC	0.186123	1.431987	2.94369	2.42E-08	4.45E-08
PNMT	1.214935	0.580306	-1.06599	3.36E-11	7.75E-11
CYP3A7	0.556541	0.147775	-1.91309	1.39E-24	1.21E-23
AOC3	67.20559	11.69627	-2.52253	3.38E-32	3.64E-30
ADSSL1	0.899895	3.174804	1.818839	1.03E-15	3.31E-15
LDHB	86.12069	179.085	1.056213	1.85E-09	3.76E-09
PRIM2	1.587571	3.362011	1.082504	3.45E-25	3.39E-24
CA2	31.95626	11.75212	-1.44318	1.84E-24	1.57E-23
DTYMK	5.28131	13.92916	1.39914	4.49E-25	4.20E-24
PDE8B	2.09482	0.833355	-1.32982	3.08E-24	2.44E-23
PTGDS	87.65013	28.54209	-1.61867	7.16E-23	4.86E-22
MGST1	20.05196	43.54529	1.118774	1.39E-11	3.35E-11
AK4	0.595046	3.925748	2.721894	5.04E-17	1.81E-16
PAH	0.010784	0.836447	6.277244	2.44E-10	5.29E-10
OPLAH	2.627307	7.297356	1.473789	1.09E-17	4.18E-17
ENTPD2	0.358122	1.647913	2.202119	6.34E-11	1.44E-10
PLA2G2F	0.001133	0.068431	5.915976	2.14E-17	7.89E-17
FMO3	7.115519	3.331032	-1.095	1.85E-17	6.92E-17
P4HA3	0.378323	1.994301	2.398194	5.16E-24	3.92E-23
UGDH	11.11011	45.11215	2.021642	6.26E-23	4.32E-22
GNPNAT1	3.653413	11.36811	1.637676	2.90E-27	4.44E-26
SRM	16.07002	35.739	1.153128	1.54E-22	1.02E-21
CAT	100.1085	29.37069	-1.76912	1.58E-32	2.61E-30
PAPSS2	55.91883	22.05491	-1.34223	1.01E-25	1.12E-24
G6PC2	0.04227	0.008296	-2.34912	2.57E-09	5.15E-09
ENTPD8	0.074407	1.856013	4.640619	2.66E-26	3.34E-25
IL4I1	1.798208	7.689022	2.09624	5.76E-16	1.88E-15
POLR2H	7.234796	17.67116	1.288373	2.02E-29	4.83E-28
MTHFD2	6.031852	19.05726	1.659668	3.45E-22	2.22E-21
PIP5K1B	7.731027	1.70966	-2.17695	3.10E-30	9.51E-29
AGMAT	0.237367	1.544106	2.701582	7.71E-29	1.64E-27
OAT	20.19376	43.52199	1.107835	6.16E-14	1.73E-13
FMO2	46.49827	5.219547	-3.15518	2.45E-32	3.13E-30
POLE2	0.424422	2.431185	2.51809	6.13E-30	1.74E-28
NMNAT2	0.439092	3.090996	2.815476	1.76E-05	2.69E-05
LYPLA1	11.61954	25.41358	1.129047	6.25E-18	2.46E-17
SGMS2	22.9437	10.17287	-1.17337	2.45E-12	6.12E-12
UCK2	1.777921	4.749837	1.417687	1.04E-09	2.18E-09

OGDHL	0.055035	0.716462	3.702475	1.58E-09	3.26E-09
PTGIS	12.30017	5.539555	-1.15084	4.21E-18	1.71E-17
GCAT	3.137662	7.498838	1.256977	4.36E-15	1.34E-14
DGKA	1.578806	3.28428	1.056743	3.18E-15	9.84E-15
CA9	0.281882	12.69819	5.493389	5.37E-23	3.75E-22
ACY3	0.405534	2.967306	2.87126	3.75E-20	1.86E-19
CYP2E1	0.094587	0.264887	1.485668	0.003246	0.004135
HAGHL	0.575599	1.511342	1.392694	2.22E-12	5.60E-12
PC	1.946121	7.100863	1.867393	1.79E-25	1.84E-24
ALOX15	9.771303	2.355191	-2.05271	4.25E-12	1.04E-11
MGAM	0.28335	0.139923	-1.01795	5.23E-09	1.01E-08
NME2	12.25682	26.65979	1.121081	2.20E-18	9.26E-18
TXNRD1	20.41002	67.0796	1.716596	6.34E-06	9.90E-06
GSR	19.6613	42.16629	1.100731	1.06E-09	2.20E-09
SORD	1.454341	4.282422	1.558061	3.86E-25	3.70E-24
CANT1	13.55119	28.54737	1.074939	2.07E-24	1.71E-23
ALDH2	50.96459	24.04908	-1.08351	1.11E-23	8.21E-23
CA7	0.01157	0.084203	2.863469	6.11E-12	1.50E-11
ASNS	2.845765	10.04325	1.819338	2.05E-27	3.42E-26
ALDH1B1	6.122095	14.50188	1.244142	9.09E-21	4.78E-20
GPD1	18.03507	1.220743	-3.88497	2.79E-30	9.32E-29
NT5E	4.455788	18.54388	2.05719	2.90E-09	5.79E-09
ACHE	0.862498	5.165416	2.582291	2.88E-07	4.99E-07
PAICS	4.651156	18.8617	2.019799	1.28E-32	2.61E-30
L2HGDH	0.815554	1.7787	1.124972	7.62E-23	5.13E-22
TSTA3	11.77304	33.45159	1.506588	1.18E-26	1.62E-25
ALOX5	46.25472	14.17334	-1.70642	4.96E-27	7.32E-26
DDO	3.127453	1.393097	-1.16669	7.74E-19	3.41E-18
ENPP6	0.21211	0.095272	-1.15469	3.94E-21	2.16E-20
DNMT3B	0.372217	1.737075	2.222443	1.59E-21	9.61E-21
GUCY1A2	2.955166	0.850718	-1.79649	5.16E-26	6.28E-25
GAMT	3.319312	7.802927	1.233131	1.92E-10	4.23E-10
NME1-NME2	0.292537	0.659072	1.171816	1.41E-10	3.13E-10
PDE1B	1.700407	0.629464	-1.43369	2.27E-24	1.85E-23
OTC	0.642895	0.051824	-3.6329	1.44E-31	7.87E-30
PLA2G4A	3.680481	21.7583	2.5636	1.58E-11	3.76E-11
CAD	3.722343	9.039393	1.280015	2.72E-22	1.77E-21
GMDS	2.596589	7.556456	1.541092	7.34E-28	1.28E-26
MAOB	18.10087	6.806286	-1.41112	1.31E-24	1.16E-23
NME4	9.120985	26.17819	1.521104	3.72E-25	3.61E-24
RDH16	0.125197	0.254347	1.022601	0.001374	0.001802
SGPL1	7.312836	16.39058	1.164364	7.13E-28	1.27E-26
CYP1A2	0.760882	0.077356	-3.29809	1.05E-30	4.52E-29

CDO1	5.110574	0.900199	-2.50517	5.25E-30	1.55E-28
GPX3	309.6442	68.40297	-2.17848	1.66E-30	5.79E-29
HK3	15.55438	4.999439	-1.63748	8.16E-24	6.08E-23
NPR1	15.98451	2.84166	-2.49187	1.29E-30	5.14E-29
B4GALT2	6.970077	17.75021	1.34859	1.12E-27	1.92E-26
SYNJ2	1.015	3.273264	1.68925	3.26E-26	4.03E-25
PKM	78.26624	163.1892	1.060084	1.30E-24	1.16E-23
AOX1	4.600314	2.276671	-1.01481	1.98E-17	7.39E-17
UGT8	0.695026	3.382405	2.282911	1.24E-08	2.32E-08
AK1	15.11649	7.186191	-1.07282	4.81E-22	3.03E-21
CBS	0.068678	0.192113	1.484028	0.000322	0.000447
AZIN2	0.543998	1.431692	1.396048	4.25E-18	1.71E-17
ADH1B	65.77864	7.174922	-3.19658	6.91E-32	4.42E-30
GOT1	11.77184	25.40319	1.10967	1.07E-20	5.58E-20
ACP6	0.91972	2.519866	1.45408	6.11E-25	5.52E-24
AANAT	0.062977	0.164643	1.386454	2.75E-07	4.80E-07
FMO1	0.388348	1.031506	1.409328	4.35E-09	8.49E-09
HPGDS	5.203569	2.215886	-1.23162	1.95E-18	8.26E-18
DGAT1	7.460956	15.10652	1.017739	7.03E-18	2.75E-17
ALAS2	1.418941	0.093998	-3.91604	2.94E-24	2.35E-23
CKMT1A	0.117164	0.837112	2.836891	3.46E-11	7.97E-11
CBR3	2.638131	9.202108	1.802448	5.15E-09	9.99E-09
PYGM	0.556649	0.267904	-1.05505	2.67E-21	1.49E-20
ARG2	1.24401	3.05455	1.295961	3.84E-09	7.53E-09
XDH	0.23118	4.425829	4.258859	1.72E-26	2.32E-25
IDO2	0.074673	0.166386	1.155883	0.004215	0.005309
PLA2G1B	29.70453	7.135132	-2.05767	7.63E-26	9.01E-25
BCO1	0.168468	0.9749	2.532776	1.27E-10	2.84E-10
OXCT2	0.073875	0.236058	1.675974	9.45E-09	1.79E-08
ATIC	10.37542	26.80955	1.369577	1.41E-30	5.14E-29
DNMT3A	2.297156	4.798356	1.062691	1.10E-18	4.78E-18
GMPPA	4.875923	10.74442	1.13984	1.03E-29	2.81E-28
BST1	4.971782	2.456348	-1.01725	6.43E-22	4.01E-21
ISYNA1	6.26707	15.16714	1.275086	1.03E-13	2.84E-13
BDH1	0.971584	2.700225	1.474669	4.82E-20	2.33E-19
POLR3G	0.612907	1.233502	1.00902	2.53E-07	4.43E-07
SULT1A3	0.016803	0.03913	1.219534	4.60E-08	8.35E-08
AHCY	22.26135	46.90591	1.075229	3.12E-21	1.72E-20
ITPKA	0.191167	7.28925	5.252861	2.13E-25	2.15E-24
ACMSD	0.073282	0.721089	3.298642	7.42E-09	1.41E-08
PDE2A	2.806664	0.901475	-1.6385	1.91E-24	1.61E-23
HAL	0.347448	2.285429	2.717595	8.42E-07	1.42E-06
ETNK2	1.058564	3.123499	1.561055	4.09E-12	1.01E-11
DMGDH	0.183331	0.706405	1.946046	0.000122	0.000176

G6PD	18.99821	52.44721	1.465003	1.58E-05	2.42E-05
TAT	0.071223	0.188067	1.400839	0.000349	0.000482
PLPP3	30.98097	12.88434	-1.26576	3.92E-28	7.34E-27
GCLM	4.878471	11.09823	1.185829	8.87E-10	1.86E-09
GPX2	1.210418	74.35104	5.940775	3.05E-07	5.27E-07
GPI	17.91683	42.96202	1.261746	1.06E-26	1.48E-25
CBR1	21.20808	77.41373	1.867975	7.37E-07	1.25E-06
GSTM5	2.610758	0.497459	-2.39182	5.43E-31	2.60E-29
PCYT1B	0.736102	0.200962	-1.87298	1.27E-20	6.56E-20
PYCR1	3.075311	44.82049	3.865354	1.18E-32	2.61E-30
ADCY8	0.713828	0.07939	-3.16855	1.37E-29	3.50E-28
HMBS	2.993156	7.429838	1.311663	6.11E-25	5.52E-24
PTGES	2.951446	25.11033	3.088787	2.30E-26	3.04E-25
PDE1C	0.931023	0.404479	-1.20275	4.29E-21	2.30E-20
CPT1B	0.186009	0.747218	2.006158	6.73E-16	2.19E-15
CEL	0.188049	1.677859	3.157441	0.000826	0.001102
MIF	17.81028	44.55402	1.322846	4.76E-19	2.16E-18
P4HA1	18.91614	40.10955	1.084328	3.57E-18	1.47E-17
PMM2	1.22495	3.000403	1.292433	3.72E-29	8.38E-28
PLA2G2C	0.01501	0.151089	3.331384	3.73E-18	1.53E-17
PLCE1	3.405438	1.448682	-1.2331	2.52E-19	1.16E-18
GART	4.83038	10.18169	1.075769	3.10E-30	9.51E-29
PSPH	2.925579	7.655782	1.387828	1.54E-17	5.83E-17
LPL	51.31269	13.22339	-1.95622	9.88E-26	1.11E-24
ACSM4	0.024818	0.10175	2.035562	0.000589	0.000798
TYMS	2.492057	10.49907	2.074852	3.74E-27	5.62E-26
CP	4.276879	58.89389	3.783488	3.67E-22	2.34E-21
GPT2	1.069293	12.05352	3.494725	4.27E-32	3.64E-30
QPRT	4.607712	15.2275	1.724556	4.04E-06	6.39E-06
ACACB	2.35728	1.008217	-1.22532	2.99E-20	1.49E-19
PLA2G2D	1.772855	4.273007	1.269177	4.08E-05	6.09E-05
TYRP1	1.471967	0.725511	-1.02067	2.05E-13	5.58E-13
CKM	0.116687	1.074732	3.203257	4.41E-13	1.16E-12
ACADL	6.603539	0.815548	-3.0174	6.69E-32	4.42E-30
PDE5A	6.311687	2.338649	-1.43235	1.09E-25	1.18E-24
LTC4S	0.09649	0.030889	-1.64328	4.48E-16	1.47E-15
MIOX	0.054551	1.009224	4.209504	4.02E-21	2.19E-20
GPT	0.293514	1.260494	2.102488	2.02E-10	4.42E-10
LPGAT1	5.6419	13.58678	1.267951	1.80E-25	1.84E-24
GAD1	0.036469	0.539918	3.887993	4.16E-21	2.25E-20
ACP5	132.7722	41.05215	-1.69342	2.76E-27	4.32E-26
PAFAH1B3	5.402157	27.44948	2.345171	9.34E-29	1.94E-27
CA4	19.03914	1.007447	-4.24019	9.60E-32	5.66E-30
HMOX1	81.32346	27.95556	-1.54054	9.19E-07	1.53E-06

FTCD	0.03249	0.242418	2.899423	2.85E-10	6.15E-10
IDH2	23.39706	58.19575	1.314587	2.32E-24	1.87E-23
PYCR3	2.724226	7.842162	1.525405	1.10E-25	1.18E-24
AK7	2.581035	1.095922	-1.2358	2.40E-11	5.61E-11
TDO2	0.331708	1.244674	1.907781	2.90E-14	8.39E-14
HGD	0.582679	8.431979	3.855098	7.74E-09	1.47E-08
ADCY10	0.019379	0.047851	1.304046	1.78E-05	2.73E-05
POLE	1.260238	2.751164	1.126346	7.72E-18	2.99E-17
CYP2D6	0.248828	1.070527	2.105098	2.01E-09	4.06E-09
ASAH2	0.064859	0.153057	1.238683	1.10E-12	2.81E-12
GFPT1	9.261466	23.06529	1.316411	2.68E-28	5.15E-27
TPI1	91.29131	218.31	1.257829	2.49E-26	3.23E-25
CNDP1	0.012887	0.035739	1.471624	0.008021	0.009875
DGAT2	1.021776	2.077008	1.023428	1.82E-07	3.20E-07
AWAT2	0.410375	0.074934	-2.45325	1.18E-18	5.04E-18
PLPP2	2.282388	13.31086	2.543988	2.43E-27	3.88E-26
RRM2	1.120227	11.8647	3.404813	1.17E-29	3.10E-28
CYP26A1	0.044925	0.32351	2.848223	7.58E-05	0.000111
PFKP	7.575957	36.97509	2.287054	1.61E-24	1.39E-23
PDE11A	0.024062	0.078069	1.697991	0.000664	0.000891
ALDH18A1	9.250779	31.39324	1.762807	7.09E-33	2.61E-30
PHOSPHO1	1.010166	0.367421	-1.45909	6.53E-19	2.90E-18
ACY1	0.763196	1.616246	1.082522	1.91E-19	8.92E-19
ADA	1.719954	3.477062	1.015499	2.92E-10	6.26E-10
NT5C1A	0.046354	0.282322	2.606587	0.001163	0.001533
KMO	1.922904	0.868783	-1.14622	1.23E-17	4.68E-17
PLA2G5	1.834756	0.82291	-1.15678	1.18E-19	5.60E-19
GUCY2C	0.229144	0.82302	1.844674	1.30E-09	2.69E-09
SULT1A1	6.111169	2.790438	-1.13096	1.39E-20	7.11E-20
GSTA3	1.549264	0.27052	-2.51778	2.15E-21	1.23E-20
SGPP2	9.505066	26.73751	1.492097	2.88E-15	8.96E-15
LDHA	51.30674	144.9287	1.498123	2.40E-28	4.73E-27
FBP1	182.6302	63.69138	-1.51976	4.87E-23	3.46E-22
CYP2F1	1.742859	0.426925	-2.0294	2.24E-13	6.08E-13
GALE	9.339842	21.1092	1.176402	1.11E-19	5.31E-19
AOC1	0.341036	11.1892	5.036037	7.25E-15	2.21E-14
MARS2	1.74322	3.61019	1.05032	1.71E-23	1.25E-22
TPO	0.036376	0.098712	1.440224	4.27E-05	6.35E-05
CA6	0.011205	0.295806	4.722459	0.000475	0.00065
ENPP2	20.88041	9.058114	-1.20487	2.45E-09	4.93E-09
UGT2B11	0.002737	0.592225	7.757568	2.32E-08	4.27E-08
NME1	3.856292	19.47312	2.336198	1.35E-30	5.14E-29
CKMT1B	0.124265	0.939737	2.918839	4.19E-11	9.63E-11
EPRS	16.5197	34.72756	1.071893	2.11E-27	3.45E-26

DGKI	0.022458	0.10403	2.211684	1.19E-15	3.80E-15
CDA	3.299273	19.26112	2.545472	9.47E-05	0.000138
PKLR	0.00589	0.032839	2.479139	0.000964	0.001279
UGT2B15	0.029279	1.637921	5.805857	1.17E-07	2.09E-07
HPRT1	10.95809	24.89861	1.184069	1.86E-21	1.09E-20
CA5A	0.00867	0.038836	2.163226	4.89E-09	9.52E-09
ACSS3	2.395515	0.858073	-1.48116	2.66E-26	3.34E-25
LRAT	0.171735	0.749572	2.125884	2.52E-06	4.07E-06
ASPA	1.173114	0.201983	-2.53804	1.06E-30	4.52E-29
SDS	1.593905	4.336223	1.443873	5.61E-09	1.08E-08
CPOX	3.604819	7.598325	1.075755	7.62E-27	1.10E-25
SCLY	0.135643	0.292157	1.106933	4.36E-20	2.12E-19
PGM2L1	1.338475	5.627084	2.071797	1.02E-26	1.45E-25
GCLC	2.79815	14.65564	2.388911	1.11E-11	2.69E-11
ADCY4	3.584324	1.099103	-1.70537	1.33E-25	1.41E-24
ACSM2A	0.011766	0.003041	-1.95185	1.09E-14	3.30E-14
CYP1A1	3.149282	1.034975	-1.60543	1.73E-12	4.36E-12
CA3	5.374487	1.309692	-2.0369	2.01E-21	1.16E-20
FLAD1	5.006479	14.45154	1.529355	2.71E-31	1.39E-29
INMT	85.88339	8.09145	-3.40791	3.92E-32	3.64E-30
CYP26B1	2.486412	1.227255	-1.01863	0.000262	0.000366
LCT	0.011732	0.235511	4.327242	3.24E-08	5.90E-08
PLA2G3	1.981445	0.853581	-1.21495	8.47E-22	5.24E-21
AKR1B10	0.289059	48.66904	7.395498	1.21E-12	3.08E-12
SHMT2	8.022795	24.42929	1.606435	2.21E-29	5.14E-28
NOS1	0.915885	0.2658	-1.78483	2.65E-25	2.64E-24
INPP4B	1.147797	2.445732	1.091398	3.56E-06	5.68E-06
PSAT1	1.117012	14.35338	3.683673	6.69E-29	1.47E-27
CTH	1.373128	3.411248	1.312834	4.16E-16	1.38E-15
RDH10	8.644265	25.29016	1.548761	3.56E-07	6.14E-07
PPAT	0.994408	4.311563	2.116301	1.70E-32	2.61E-30
INPP5K	15.61732	7.770189	-1.00713	1.69E-29	4.19E-28
ALDH3B2	0.525746	4.383546	3.059661	1.50E-16	5.22E-16
TK1	5.027055	35.35409	2.814092	5.79E-28	1.06E-26
ALDOA	95.59709	239.0571	1.322316	5.12E-25	4.73E-24
ACSM5	0.548713	0.27399	-1.00193	2.46E-17	9.04E-17

gene	conMean	treatMean	logFC	pValue	fdr
AL513548.3	0.00616	0.012508	1.021908	0.014457	0.019379
AC106882.1	0.021996	0.171364	2.961758	5.23E-13	3.13E-12
AC037198.1	0.441846	2.778472	2.652675	0.010683	0.014627
AL355482.1	0.01112	0.045803	2.04235	0.004574	0.006643
AC007785.3	0.041422	0.111191	1.42456	0.000151	0.000274
AC011511.5	3.244186	0.606559	-2.41914	1.06E-10	4.64E-10
AL138789.1	0.003422	0.186203	5.765884	5.55E-17	5.96E-16
AC130650.2	0.213649	0.629466	1.558886	0.000243	0.000427
AC005034.2	0.067594	0.219027	1.696136	0.03233	0.041121
AC087741.2	0.827657	1.880058	1.183673	6.87E-10	2.67E-09
AC091053.2	0.036088	0.14404	1.996881	0.007146	0.010072
LINC01816	0.427541	1.273491	1.574653	4.23E-11	1.97E-10
AC005220.1	0.011989	0.032552	1.441046	0.000302	0.000523
AC011294.1	0.015767	0.608152	5.269407	1.92E-25	7.77E-24
RNF157-AS1	0.461306	1.161545	1.332249	7.48E-10	2.89E-09
AP001351.1	0.084526	0.20709	1.292798	9.28E-08	2.71E-07
AL390778.2	0.414264	0.167029	-1.31045	3.81E-11	1.79E-10
AC005064.1	0.02092	0.147755	2.820277	0.00659	0.009339
C10orf25	1.654547	0.758914	-1.12443	5.31E-25	1.88E-23
AC100797.1	0.095081	0.193571	1.025644	0.039425	0.049416
LINC01398	0.022124	0.045396	1.03693	0.005871	0.008377
AL138760.1	0.012839	0.758186	5.883906	3.55E-21	6.56E-20
AL109761.1	0.074411	0.577189	2.955452	3.10E-11	1.48E-10
P3H2-AS1	0.614302	0.210468	-1.54535	1.23E-20	2.07E-19
AC073529.1	0.039461	0.145981	1.887276	4.22E-20	6.81E-19
AC243972.1	0.010591	0.067042	2.662292	3.75E-08	1.16E-07
DDN-AS1	0.077907	0.502445	2.689148	4.26E-26	1.95E-24
AC244502.3	0.018879	0.065995	1.805576	2.29E-05	4.67E-05
AC016747.2	0.129156	0.331954	1.361864	0.000234	0.000412
AL049794.1	0.041436	0.149786	1.853959	1.32E-10	5.68E-10
AC024132.3	0.11016	0.006653	-4.04948	4.79E-27	2.61E-25
AC239803.2	0.051525	0.120201	1.222108	0.000241	0.000425
AP002992.1	0.050739	0.185593	1.870981	1.98E-06	4.75E-06
AL109924.2	0.00442	0.046715	3.401648	6.99E-10	2.71E-09
AC037459.2	0.713056	0.317738	-1.16618	1.06E-26	5.32E-25
AC020663.3	0.096913	0.305522	1.656515	2.77E-05	5.57E-05
RSF1-IT1	0.039012	0.176477	2.177502	0.00013	0.000238
AL132655.2	0.06454	0.173735	1.428631	0.010428	0.014299
AC125603.2	0.067574	2.163818	5.000966	0.011159	0.015241
AC092078.2	0.110433	0.007196	-3.93974	6.35E-39	9.87E-36
AL121790.1	0.062864	0.173937	1.468259	0.033666	0.04268
AC131254.1	0.014719	0.038164	1.374552	7.77E-10	2.99E-09
AC069431.1	0.006134	0.019339	1.656473	0.003044	0.004553

AL031658.1	0.237695	0.07457	-1.67244	2.78E-16	2.66E-15
AC012236.1	0.594461	2.234327	1.910186	2.75E-17	3.06E-16
AC103764.1	0.006623	0.018534	1.484583	0.006366	0.009051
AL033519.3	2.337277	1.016631	-1.20103	5.22E-17	5.62E-16
AC078850.2	0.017358	0.173849	3.324196	2.06E-05	4.24E-05
AC127070.1	0.145487	0.379031	1.381431	1.54E-09	5.68E-09
AC246787.2	0.070318	0.362136	2.364564	2.23E-09	7.97E-09
AL117190.1	0.013854	0.126712	3.193156	0.004475	0.006503
SLC12A5-AS1	0.066044	0.241124	1.868266	1.58E-09	5.81E-09
LINC00970	0.01867	0.07568	2.019172	1.22E-06	3.02E-06
AC068620.2	0.249859	0.501154	1.004138	2.27E-05	4.65E-05
AL096865.1	0.104267	0.568431	2.446706	7.22E-21	1.27E-19
AL450992.1	0.017776	0.045403	1.352871	0.000566	0.000949
THORLNC	0.165634	0.374929	1.178619	3.75E-07	9.98E-07
AC022509.2	2.176976	0.626674	-1.79654	4.89E-15	3.96E-14
LINC01395	0.008245	0.425619	5.689869	1.43E-17	1.68E-16
AL049830.3	0.015879	0.119529	2.91213	1.18E-10	5.11E-10
AC010320.4	0.021163	0.108228	2.354432	0.038624	0.048481
AC096719.1	0.014345	0.051511	1.844297	4.50E-07	1.19E-06
HOTAIR	0.00275	0.442803	7.331306	1.56E-10	6.65E-10
AC002076.1	0.021092	0.468775	4.474113	7.00E-15	5.52E-14
AP003721.1	0.014437	0.082503	2.514637	8.69E-09	2.90E-08
AL031963.2	0.041943	0.160367	1.934878	6.28E-11	2.84E-10
AC074044.1	0.048762	0.147948	1.601256	3.87E-09	1.34E-08
AC105450.1	0.013247	0.169526	3.677816	1.06E-11	5.36E-11
AC008870.3	0.013148	0.105472	3.003975	4.35E-12	2.33E-11
DLEU1	0.323421	0.726056	1.166666	2.87E-11	1.38E-10
AC137932.3	0.25833	0.648461	1.327802	1.23E-11	6.20E-11
SFTA1P	83.99271	25.41695	-1.72447	2.16E-23	5.53E-22
AC112722.1	1.608584	0.456926	-1.81576	9.54E-20	1.49E-18
LINC01093	0.132201	0.059901	-1.14207	1.74E-12	9.81E-12
AC108463.3	0.054638	0.184055	1.752167	5.78E-05	0.000112
AC010913.1	0.022894	0.054249	1.244601	6.69E-07	1.71E-06
LINC01982	0.217343	0.065949	-1.72056	2.46E-12	1.35E-11
AC068196.1	0.015951	0.052492	1.718489	7.03E-06	1.55E-05
AC008897.3	0.626017	0.221423	-1.4994	1.92E-18	2.52E-17
MIR2052HG	0.006415	0.109661	4.095426	2.11E-07	5.82E-07
AC092279.1	1.070097	2.359633	1.140821	0.000385	0.000658
SATB2-AS1	0.003959	0.04054	3.35602	1.39E-15	1.21E-14
AL670729.1	0.481976	1.491099	1.629342	8.00E-08	2.36E-07
FAM95B1	0.016521	0.006234	-1.40619	6.71E-10	2.61E-09
AP003393.1	0.026887	0.01147	-1.22903	2.08E-06	4.96E-06
AL390067.1	0.165832	0.355049	1.098295	0.001046	0.001683
LENG8-AS1	1.015285	2.118077	1.06087	2.05E-12	1.14E-11

AC114316.2	0.005785	0.087003	3.910593	1.01E-05	2.17E-05
AC009226.1	0.068844	0.301439	2.130468	1.45E-22	3.22E-21
AC127024.4	0.687244	1.54404	1.167817	5.10E-07	1.33E-06
AL662791.1	0.270053	0.870644	1.688838	1.20E-13	7.78E-13
LINC00621	0.036426	0.10976	1.59132	3.08E-05	6.15E-05
SLC12A9-AS1	0.266176	1.316483	2.306234	1.57E-23	4.11E-22
AC106772.1	0.003114	0.051324	4.04282	2.42E-09	8.62E-09
AC117383.1	0.046597	0.136468	1.550263	0.003442	0.005106
AL008718.2	0.022554	0.049987	1.148146	0.000295	0.000513
AC003092.1	0.011595	0.596836	5.685732	1.27E-06	3.14E-06
AC008268.1	21.51801	5.706635	-1.91483	4.68E-23	1.13E-21
AC099811.4	0.041658	0.377154	3.17849	0.00777	0.010884
AL034345.2	0.00506	0.05491	3.439798	1.94E-10	8.10E-10
LINC01991	0.015231	0.033082	1.119002	4.42E-05	8.69E-05
AC138466.1	0.07376	0.28077	1.928477	1.27E-07	3.61E-07
LINC00887	0.00854	0.139693	4.031932	2.06E-18	2.70E-17
AC245100.7	0.18011	0.526279	1.546946	0.000256	0.000449
NAALADL2-AS2	0.00849	0.688572	6.341736	3.11E-11	1.48E-10
AC103740.2	0.005856	0.106325	4.182528	1.96E-14	1.44E-13
Z99943.1	0.045137	0.262516	2.540035	3.64E-08	1.13E-07
AC104458.1	0.094506	0.027036	-1.80552	2.99E-13	1.84E-12
AJ239322.1	0.024099	0.006755	-1.83484	9.05E-13	5.28E-12
AL136298.1	0.005432	0.096781	4.15511	6.09E-07	1.57E-06
AC068724.1	0.025334	0.164787	2.701467	9.23E-14	6.10E-13
AC010255.2	1.194753	0.328287	-1.86368	2.32E-08	7.37E-08
AC010542.4	0.010705	0.023341	1.124657	2.65E-05	5.36E-05
AC093909.4	0.017615	0.004852	-1.86	1.60E-09	5.86E-09
AC087667.1	0.013978	0.156276	3.48287	2.00E-09	7.22E-09
RASGRF2-AS1	0.04227	0.090952	1.105482	0.000633	0.001051
AC097634.1	0.113027	0.309121	1.451503	0.000371	0.000636
LINC01232	0.298787	0.901025	1.59245	1.35E-14	1.01E-13
AC011700.1	0.005677	0.071909	3.662961	2.25E-05	4.61E-05
AC073195.1	0.206744	0.554596	1.423593	2.73E-15	2.30E-14
FARP1-AS1	0.098435	0.21055	1.096922	0.011854	0.016098
LINC02555	3.078868	0.907414	-1.76257	8.75E-23	2.04E-21
LINC02579	0.010479	0.026863	1.358081	4.68E-06	1.06E-05
AC024267.5	0.052497	0.252976	2.268705	5.46E-05	0.000106
DUXAP8	0.056403	0.383932	2.767	3.09E-12	1.67E-11
AC073326.1	0.056389	0.213766	1.922551	0.003257	0.004846
AC130651.1	0.044708	0.217903	2.285092	5.22E-06	1.17E-05
AL121845.4	0.21594	0.503459	1.221245	1.32E-05	2.80E-05
AL121917.2	0.133887	0.338516	1.338203	1.99E-05	4.10E-05
FAM30A	0.20716	0.686697	1.728931	8.28E-10	3.17E-09
AP005264.1	0.309617	0.088814	-1.80162	1.43E-21	2.78E-20

MIR193BHG	0.282511	0.860439	1.606767	1.21E-08	3.96E-08
AP002360.2	0.050517	0.144947	1.520674	2.39E-06	5.63E-06
AC068831.1	0.056489	0.155282	1.458845	2.18E-17	2.47E-16
HOXC13-AS	0.003469	0.33507	6.593993	5.20E-11	2.39E-10
AL669970.3	0.129002	0.05904	-1.12762	1.27E-11	6.36E-11
AC007671.1	0.485075	0.136581	-1.82845	5.14E-21	9.18E-20
AC073842.1	0.035405	0.092763	1.389616	0.002258	0.003441
AC007221.1	0.112047	0.028386	-1.98085	2.28E-09	8.14E-09
AC147651.2	0.07915	0.025373	-1.6413	8.89E-08	2.60E-07
AC015909.1	0.01978	0.129875	2.71499	5.97E-11	2.71E-10
AL139231.1	0.084548	0.028691	-1.5592	6.93E-07	1.77E-06
AC090709.1	0.01247	0.151781	3.605459	2.65E-07	7.21E-07
AC010976.2	2.284019	0.285992	-2.99753	2.43E-28	1.93E-26
GAS6-DT	1.025992	0.501885	-1.03159	5.79E-22	1.16E-20
AC092802.1	0.268526	0.130904	-1.03655	2.00E-09	7.22E-09
AC092168.2	0.048257	0.384655	2.994754	0.001422	0.002238
LINC01348	0.107849	0.770724	2.837201	9.85E-17	1.01E-15
AP000593.3	0.093008	0.356862	1.939935	4.13E-11	1.93E-10
CYP4A22-AS1	0.061892	0.400028	2.692272	3.80E-21	6.95E-20
LINC02206	0.067479	0.023721	-1.5083	1.27E-09	4.71E-09
LINC02474	0.060166	1.907301	4.986441	2.43E-13	1.51E-12
AL035416.1	0.056036	0.138055	1.300806	0.015419	0.020557
NFYC-AS1	0.377671	0.89496	1.244695	1.13E-10	4.91E-10
AC022819.1	0.021872	0.048891	1.160498	6.58E-05	0.000126
AL080317.2	0.158256	0.391823	1.307946	6.03E-05	0.000116
AL512343.2	0.183662	0.508804	1.470058	7.91E-08	2.34E-07
AC010998.1	0.926104	0.196784	-2.23456	8.57E-28	5.49E-26
LINC02365	0.024138	0.300546	3.638211	3.28E-07	8.77E-07
AL049871.1	0.230135	0.080637	-1.51297	7.45E-15	5.84E-14
AC018682.2	0.023241	0.052592	1.178171	0.000974	0.001574
AC245595.1	0.253597	0.530752	1.0655	0.000874	0.001422
AC108727.1	0.228159	0.619223	1.44042	0.005568	0.007976
AC093484.4	0.500153	1.283728	1.359897	1.00E-09	3.79E-09
LINC00470	0.0157	0.190932	3.604212	0.021633	0.028272
AC092143.3	0.099146	0.34189	1.785905	2.54E-10	1.04E-09
AC091132.2	0.104163	0.269154	1.369593	5.72E-07	1.48E-06
AC069257.1	0.064576	0.20316	1.653538	0.000149	0.00027
AL451050.2	0.088629	0.256206	1.531454	2.81E-10	1.15E-09
AC104809.1	0.238464	0.113177	-1.07519	1.24E-07	3.54E-07
LINC01361	0.009857	0.051329	2.380476	0.00104	0.001675
AC027277.2	1.950671	0.610201	-1.67661	6.63E-20	1.05E-18
LINC01235	0.145888	0.328456	1.17084	2.12E-08	6.76E-08
LINC02126	0.426569	0.203252	-1.06951	6.06E-12	3.18E-11
MCM8-AS1	0.020137	0.057938	1.524673	0.003274	0.004868

AL355490.2	0.038679	0.082617	1.094898	8.67E-05	0.000163
AL162412.1	0.03166	0.06893	1.122461	0.029359	0.037596
AC128709.3	1.168588	0.064669	-4.17556	2.17E-35	1.35E-32
AP000526.1	0.003559	0.061554	4.112152	1.46E-10	6.25E-10
AP4B1-AS1	0.193107	0.466028	1.271016	2.85E-07	7.70E-07
AC117498.2	0.233209	0.653223	1.485953	1.67E-05	3.48E-05
LINC01412	0.418907	0.099461	-2.07442	2.49E-24	7.74E-23
AP001469.2	0.249477	0.630251	1.33702	3.15E-09	1.10E-08
AC002116.2	0.290285	0.71775	1.306012	6.63E-16	5.98E-15
Z99289.2	0.013257	0.083953	2.662803	0.000926	0.0015
AC073172.1	0.061154	0.021819	-1.48685	8.14E-07	2.06E-06
AC026785.3	0.004204	1.887512	8.810451	5.70E-11	2.59E-10
LINC01010	0.491447	0.219896	-1.16022	0.003665	0.005408
AC133785.1	0.001372	0.204707	7.221546	2.40E-23	6.10E-22
LINC01695	0.006129	0.03666	2.580553	0.029014	0.037184
AL583810.1	0.094111	0.212164	1.172746	1.23E-05	2.63E-05
AL132822.1	0.180906	0.66472	1.87751	0.001996	0.003074
AC022144.1	0.422034	1.382472	1.71182	5.98E-12	3.14E-11
LINC02065	0.006594	0.122489	4.215449	1.64E-11	8.14E-11
AL590729.1	0.091605	0.263726	1.525546	3.95E-07	1.05E-06
AL157904.1	0.051134	0.106775	1.06222	0.000412	0.000702
AL035446.1	3.477663	1.453734	-1.25835	9.23E-16	8.17E-15
AC005162.2	0.021847	0.112614	2.365877	0.017011	0.022521
LINC01700	0.047744	0.020595	-1.21306	3.23E-08	1.01E-07
AC087289.2	0.190779	0.408273	1.097633	6.75E-10	2.63E-09
FLJ33534	0.018059	0.075758	2.06868	9.88E-14	6.50E-13
AC023796.2	0.015068	0.267408	4.149446	1.18E-13	7.64E-13
LINC01765	3.13556	0.548604	-2.51489	2.63E-16	2.53E-15
LINC00494	0.051221	0.131028	1.35506	0.001119	0.001791
AC108516.2	0.041012	0.112596	1.457038	0.000143	0.000261
AC008496.2	0.063785	1.80783	4.824887	2.69E-15	2.27E-14
AC023855.1	0.022839	0.095277	2.060619	0.000768	0.001258
AL080312.2	0.02673	0.093308	1.803527	0.000145	0.000263
PGM5P4-AS1	0.240432	0.068122	-1.81943	1.11E-23	2.99E-22
PCAT19	6.668614	1.296384	-2.36289	3.57E-31	6.72E-29
AC068870.2	6.548311	2.896404	-1.17686	1.13E-22	2.57E-21
GDNF-AS1	0.007429	0.03841	2.370281	2.91E-05	5.84E-05
LINC01090	0.01636	0.059313	1.858188	1.95E-05	4.02E-05
AC023908.3	0.081372	0.21013	1.368672	1.38E-10	5.92E-10
AC103923.1	0.025877	0.088763	1.778274	3.51E-08	1.09E-07
LINC01114	0.013401	0.005201	-1.36557	2.50E-08	7.89E-08
AP000424.2	0.048528	0.307106	2.661835	3.17E-10	1.28E-09
AL359853.1	0.023717	0.189118	2.995309	7.56E-06	1.66E-05
AP002478.1	0.0265	0.26707	3.333151	0.003627	0.005356

AL357153.1	0.063657	0.142723	1.164824	0.000586	0.000982
AL109945.1	0.012146	0.03679	1.598797	2.55E-05	5.17E-05
AC027228.1	0.034235	0.185848	2.440579	0.023479	0.030506
AC010531.5	0.119919	0.285757	1.25273	2.59E-05	5.24E-05
AC124068.2	0.134923	0.27378	1.020882	5.24E-08	1.58E-07
LINC00908	0.617497	0.2142	-1.52747	9.03E-18	1.09E-16
AC022217.2	0.232788	0.073771	-1.65789	4.39E-10	1.74E-09
AC010894.2	0.349284	0.843816	1.272527	9.35E-12	4.78E-11
LINC00609	0.026899	0.237028	3.139462	0.018854	0.02485
AC092115.3	0.071617	0.573291	3.000887	9.77E-23	2.27E-21
AC021851.1	0.199294	0.560917	1.492888	3.81E-15	3.12E-14
AL161668.4	0.076929	0.032211	-1.25599	3.15E-11	1.50E-10
AP002852.1	0.045095	0.118908	1.398808	7.72E-06	1.70E-05
AC084859.1	0.325422	0.721513	1.148714	0.000102	0.00019
AC106712.1	0.020246	0.087106	2.105158	0.000172	0.000309
AL163952.1	0.01581	0.070023	2.146983	2.29E-05	4.67E-05
AP001619.2	0.012618	0.088834	2.81568	1.03E-08	3.41E-08
CYTOR	1.740607	4.547679	1.38554	1.51E-20	2.53E-19
AC108868.1	0.204364	0.054519	-1.9063	2.04E-18	2.68E-17
AL139353.2	0.166398	0.342516	1.041536	8.58E-07	2.17E-06
AL590705.3	0.525508	1.494421	1.507803	7.25E-06	1.60E-05
DLG3-AS1	0.118679	0.379225	1.675988	6.90E-14	4.65E-13
AC022960.2	0.030396	0.223564	2.878748	0.009983	0.013737
AC002519.1	0.019937	0.057265	1.522226	0.000351	0.000603
AC008149.1	0.046461	0.170107	1.872362	0.016771	0.022246
AC015813.1	1.157419	2.661786	1.201484	8.88E-06	1.93E-05
AC079630.1	12.20104	3.61905	-1.75332	1.05E-23	2.85E-22
AC061992.1	0.476478	1.312203	1.46151	3.57E-11	1.68E-10
AL355574.1	1.070581	2.209734	1.045479	9.61E-14	6.33E-13
LINC01612	2.369043	1.059123	-1.16143	8.40E-18	1.02E-16
LINC01303	0.019346	0.123288	2.671917	2.45E-16	2.36E-15
SAMMSON	0.003896	0.041118	3.399829	5.03E-08	1.53E-07
AL121759.1	0.010195	0.060736	2.574751	0.012108	0.016424
SLC7A11-AS1	0.029073	0.11548	1.989896	9.92E-05	0.000185
AC079834.2	0.128955	0.470947	1.868702	2.60E-15	2.19E-14
AC138474.1	0.133523	0.059703	-1.16121	1.05E-22	2.41E-21
VPS33B-DT	0.033933	0.068087	1.004697	2.42E-06	5.71E-06
AC087588.2	0.0763	0.308349	2.014815	6.43E-06	1.43E-05
LINC01513	1.032521	0.279106	-1.88729	3.10E-13	1.90E-12
AC005865.1	0.014977	0.140218	3.226842	2.75E-15	2.31E-14
AL139424.1	0.013227	0.04628	1.806929	0.000876	0.001424
AC005695.3	0.086122	0.18861	1.130951	0.000615	0.001024
AC104248.1	0.037859	0.303543	3.003212	5.43E-15	4.35E-14
C5orf66	0.087198	0.175114	1.005922	1.11E-09	4.17E-09

AC022431.1	0.052467	0.021691	-1.27433	5.55E-06	1.24E-05
AC079354.3	0.021695	0.122923	2.502299	1.85E-09	6.72E-09
AC024257.1	0.509907	0.14286	-1.83563	9.26E-23	2.16E-21
AC027449.1	0.329896	0.158481	-1.0577	6.26E-13	3.71E-12
CLLU1	0.002169	0.008536	1.976521	0.008109	0.011327
AC243830.2	0.074608	0.282168	1.919154	2.26E-10	9.35E-10
NAV2-AS2	0.935264	0.183481	-2.34974	1.10E-15	9.61E-15
HOXA11-AS	0.005324	0.143449	4.75175	3.37E-08	1.05E-07
LINC02012	0.081586	0.247728	1.60237	1.94E-08	6.22E-08
AC010275.1	0.008744	0.394492	5.495602	3.18E-09	1.11E-08
PRMT5-AS1	0.050373	0.134129	1.412905	1.57E-12	8.89E-12
AL163953.1	0.002467	0.201102	6.34912	7.55E-17	7.94E-16
CASC8	0.044205	0.43524	3.299545	5.51E-11	2.51E-10
AP000695.2	0.158433	0.533113	1.750568	2.97E-10	1.21E-09
AC092809.2	0.602311	0.298596	-1.01232	1.29E-16	1.30E-15
LINC01645	1.000352	0.174333	-2.52059	1.07E-27	6.75E-26
AL356234.2	0.017272	0.487126	4.817755	8.42E-10	3.21E-09
AL391832.1	0.038711	0.083199	1.103816	0.002832	0.004251
LINC01564	0.174367	1.426612	3.032391	3.19E-10	1.29E-09
AC007663.4	0.236978	0.527689	1.154938	7.46E-11	3.33E-10
AC073115.1	0.050315	0.173627	1.786924	4.90E-05	9.58E-05
IDH1-AS1	0.355999	0.76755	1.108389	4.56E-09	1.57E-08
AL033397.1	0.035207	1.804618	5.679708	6.44E-07	1.65E-06
AL353803.1	0.006153	0.04496	2.869371	8.42E-07	2.13E-06
AC073257.2	0.229914	0.520445	1.17865	0.000494	0.000834
AL049651.2	0.196308	0.011579	-4.0836	2.42E-39	7.51E-36
AL359922.2	0.075829	0.388054	2.355441	1.30E-08	4.24E-08
AL353708.3	0.117916	0.411082	1.801667	2.99E-17	3.31E-16
AC020612.3	0.075133	0.243425	1.695951	2.84E-06	6.62E-06
LINC02571	0.057681	0.203663	1.820011	0.024674	0.031924
LINC01121	0.026591	0.085313	1.681837	1.42E-05	3.01E-05
AL390115.1	0.01799	0.107882	2.584212	1.32E-09	4.91E-09
FOCAD-AS1	0.061698	0.172495	1.48327	0.000165	0.000296
AC010175.1	0.710394	0.319509	-1.15276	3.54E-15	2.93E-14
NARF-IT1	0.366314	0.832226	1.183894	1.90E-06	4.60E-06
AC114947.2	0.51103	0.170758	-1.58146	4.01E-25	1.45E-23
AC003005.2	0.019123	0.07029	1.878002	0.00142	0.002236
AC004034.1	0.123742	0.806212	2.703819	1.06E-13	6.95E-13
AC008870.4	0.071086	0.197346	1.473096	6.22E-10	2.43E-09
AC084757.2	0.030571	0.086074	1.493435	0.002933	0.004396
AC022613.2	0.877448	0.325155	-1.43219	9.79E-28	6.21E-26
AC025575.2	0.044278	1.715006	5.275487	1.09E-05	2.34E-05
AC100802.1	0.028791	0.010672	-1.43181	7.68E-14	5.13E-13
LINC01936	4.803697	0.971281	-2.30618	1.56E-26	7.54E-25

AC037487.4	0.037906	0.121453	1.679918	1.06E-07	3.07E-07
Z69666.1	0.09272	0.226052	1.2857	0.001007	0.001625
AC105219.3	0.09876	0.744814	2.91488	9.78E-26	4.14E-24
LINC01624	0.116142	0.025952	-2.16197	5.80E-27	3.06E-25
LINC02300	0.115842	0.784194	2.759047	0.025201	0.032565
AC092295.1	0.016336	0.065958	2.0135	4.86E-06	1.10E-05
AC010327.4	0.064476	0.167564	1.377866	3.72E-06	8.52E-06
AC005586.2	0.207104	0.521728	1.332943	0.003062	0.004574
AL136221.1	0.061989	0.405241	2.708694	7.65E-14	5.12E-13
SAMD12-AS1	0.270864	0.75383	1.476669	2.06E-15	1.75E-14
AP001189.5	0.203854	0.046766	-2.124	2.55E-23	6.35E-22
AC093326.1	0.075816	0.036678	-1.04758	7.21E-14	4.86E-13
AC145146.1	0.030046	0.345812	3.524758	8.30E-08	2.44E-07
C21orf91-OT1	0.013306	0.130967	3.299099	0.000619	0.00103
AL391056.1	0.067627	1.423011	4.395203	2.34E-25	9.16E-24
AC004895.1	0.031618	0.091997	1.54086	3.95E-08	1.22E-07
AL354714.3	1.068737	0.152262	-2.81128	1.11E-32	3.58E-30
SNHG25	0.744137	2.328596	1.64582	7.21E-14	4.86E-13
AP002812.3	0.130758	0.289207	1.145204	0.003922	0.00576
AC084346.2	0.02221	0.108169	2.283994	4.92E-10	1.94E-09
HAGLROS	0.128014	0.805582	2.653735	2.64E-10	1.08E-09
AC069222.1	0.021986	0.083304	1.921776	7.30E-13	4.30E-12
AC005841.1	0.039469	0.163394	2.049556	1.48E-07	4.18E-07
LINC02208	0.001547	0.024873	4.006784	7.57E-08	2.24E-07
AC012645.4	0.118726	0.337219	1.506048	1.84E-09	6.68E-09
AC025431.1	0.09166	0.024036	-1.93109	1.65E-19	2.47E-18
AC137932.2	0.106823	0.401323	1.909543	4.87E-08	1.48E-07
AC021087.2	0.075085	0.301258	2.004403	1.88E-17	2.16E-16
AC099850.1	0.947164	0.466694	-1.02114	4.42E-19	6.41E-18
AC008649.1	0.040749	0.247666	2.603571	2.44E-05	4.95E-05
AC021546.1	0.05519	0.024088	-1.19607	1.12E-08	3.69E-08
ITGB1-DT	0.044163	0.597739	3.758591	6.47E-16	5.87E-15
AL139819.1	0.118438	0.04371	-1.4381	0.000215	0.00038
MIR4713HG	0.012239	0.303804	4.633574	1.92E-10	8.03E-10
AC020931.1	0.087022	0.174716	1.005562	6.64E-05	0.000127
AC004832.1	1.192209	0.270801	-2.13833	3.48E-14	2.48E-13
BX664727.3	0.124467	0.043349	-1.52169	2.78E-14	2.01E-13
TMEM92-AS1	0.454809	1.448647	1.671373	5.19E-13	3.10E-12
AC004241.4	0.033869	0.076023	1.166476	0.021217	0.027781
AL035409.1	1.665258	0.383768	-2.11744	4.12E-26	1.91E-24
AC104134.1	0.012584	0.043895	1.802424	2.18E-06	5.17E-06
AL031770.1	0.039085	0.194928	2.318259	0.002095	0.003211
AL365199.1	0.090341	0.022342	-2.01561	4.79E-20	7.71E-19
AL078644.1	0.217333	0.551628	1.34379	2.39E-17	2.69E-16

LINC01502	0.013672	0.103995	2.927188	0.008423	0.01172
AC004223.2	0.062919	0.254096	2.013793	0.004991	0.007206
AC007285.1	0.099808	0.37883	1.92433	8.02E-19	1.12E-17
AL032819.1	0.036055	0.16282	2.175029	1.81E-07	5.02E-07
AL121672.3	0.107375	0.237344	1.144326	0.000107	0.000198
AC087627.1	0.026832	0.065843	1.295057	1.29E-05	2.75E-05
AC069200.1	0.46227	1.477701	1.676545	2.88E-08	9.03E-08
AL606469.1	4.329132	0.128163	-5.07803	2.32E-33	9.60E-31
AL390719.2	0.876383	2.979791	1.765578	1.56E-10	6.64E-10
MNX1-AS1	0.041135	1.722938	5.388372	1.56E-26	7.54E-25
AC068700.1	0.332776	0.075623	-2.13764	5.47E-28	3.74E-26
AL138733.1	0.014724	0.066591	2.177137	3.87E-05	7.64E-05
AL355340.1	0.023695	0.071331	1.589954	0.000148	0.000268
AL160314.2	0.060618	0.153632	1.341669	1.18E-08	3.87E-08
AC026369.3	8.838213	0.920836	-3.26274	1.20E-29	1.24E-27
AC006159.1	0.410879	0.158921	-1.3704	1.08E-17	1.29E-16
LINC01176	0.892056	1.926244	1.110584	9.33E-09	3.10E-08
AC106900.2	0.032102	0.922747	4.845209	1.68E-14	1.24E-13
AL451060.1	0.409453	0.86065	1.071731	0.00083	0.001355
AC010624.3	0.363222	0.149727	-1.27852	6.27E-07	1.61E-06
AC141002.1	0.288005	0.622556	1.11211	9.35E-07	2.35E-06
C11orf72	0.018568	0.058711	1.660805	2.65E-09	9.40E-09
AC025470.2	0.142728	0.029012	-2.29855	2.43E-17	2.73E-16
Z98257.1	0.061727	4.77663	6.273938	4.98E-24	1.47E-22
AC006159.2	0.161735	0.041503	-1.96235	6.73E-15	5.33E-14
MIR4527HG	0.00812	0.113929	3.810584	0.00026	0.000455
AC135012.3	0.405148	0.024849	-4.02717	9.42E-32	2.25E-29
DLEU2	0.507964	1.20014	1.240405	3.22E-11	1.53E-10
AC009041.2	0.201591	0.47168	1.226379	0.018574	0.024491
DNMBP-AS1	0.299519	0.63724	1.089192	1.52E-11	7.56E-11
AC004947.1	0.708718	0.094813	-2.90205	3.72E-27	2.08E-25
LINC01585	0.029681	0.07017	1.241287	3.94E-06	9.01E-06
CSTF3-DT	0.019936	0.05313	1.414141	1.40E-06	3.42E-06
LINC01070	0.045447	0.004058	-3.48519	4.17E-37	3.70E-34
AL008723.3	0.093359	0.276641	1.567146	0.002376	0.003612
AC009974.1	0.957167	0.444102	-1.10788	5.19E-15	4.17E-14
AL390860.1	0.014618	0.006976	-1.06731	3.23E-05	6.42E-05
PARAL1	5.862907	0.793397	-2.8855	7.45E-29	6.81E-27
AL590560.1	0.893112	2.151211	1.268237	0.0003	0.000521
AC092484.1	0.009283	0.45563	5.6171	1.43E-10	6.14E-10
AF230666.1	0.138472	0.341163	1.300863	8.52E-10	3.25E-09
AL353801.2	0.01405	0.063853	2.184163	0.016937	0.022437
AL136115.2	0.162426	0.684552	2.075377	0.000448	0.00076
AC113139.1	0.218608	0.617824	1.49885	1.27E-08	4.16E-08

LINC01138	0.456288	1.173869	1.363255	2.49E-14	1.81E-13
LINC01727	0.010607	0.038217	1.849246	1.22E-07	3.48E-07
AC005730.3	0.096708	0.369868	1.935302	4.08E-06	9.32E-06
AC093788.1	0.295118	0.837084	1.504081	5.75E-10	2.25E-09
AC109361.1	0.013817	0.033085	1.259723	0.000158	0.000286
LINC00944	0.111465	0.453874	2.025701	6.75E-09	2.29E-08
AL590491.2	0.320967	0.067515	-2.24913	1.09E-14	8.28E-14
MRGPRF-AS1	0.068014	0.02671	-1.34843	1.57E-13	1.00E-12
AL357140.2	0.268334	0.540034	1.009019	8.86E-10	3.37E-09
AC105384.1	0.415591	0.108627	-1.93578	9.13E-22	1.80E-20
AL390036.1	0.893736	0.237387	-1.91261	1.28E-22	2.88E-21
LINC01970	0.03058	0.141445	2.20959	1.88E-15	1.61E-14
AC027801.3	0.021808	0.115137	2.400397	5.04E-05	9.83E-05
LINC01132	0.200419	0.457354	1.190294	3.58E-07	9.55E-07
SYNGAP1-AS1	0.029322	0.09706	1.726901	5.12E-10	2.01E-09
AC104051.2	0.029694	0.011751	-1.33733	7.85E-15	6.10E-14
LINC01655	0.441492	2.123952	2.266291	1.00E-05	2.17E-05
AL121894.1	0.074158	0.017078	-2.1185	1.12E-15	9.80E-15
AC005083.1	2.26421	5.382653	1.24931	1.27E-08	4.18E-08
AC091488.1	0.093695	0.270646	1.53036	5.50E-09	1.88E-08
LINC01993	0.024497	0.209388	3.095507	2.03E-15	1.72E-14
AC092920.1	0.002601	0.083646	5.007364	3.38E-12	1.83E-11
AC239798.4	0.078136	0.159549	1.029936	0.008213	0.011457
LINC01600	0.007813	0.037838	2.275792	1.70E-09	6.21E-09
AC106052.1	0.051086	0.143246	1.487492	2.72E-07	7.37E-07
AC007497.1	0.117292	0.335353	1.51558	3.82E-08	1.18E-07
HHIP-AS1	6.338976	2.313574	-1.45413	2.19E-20	3.61E-19
AC092683.1	0.033242	0.077722	1.225322	1.82E-06	4.40E-06
AC021092.1	0.020282	0.059155	1.54429	6.65E-09	2.26E-08
LINC02340	0.104643	0.21024	1.006563	7.92E-06	1.74E-05
AC097641.2	0.28111	0.735325	1.387249	2.68E-11	1.29E-10
AL136038.3	0.042975	0.088357	1.03984	5.25E-05	0.000102
AC024267.4	0.053463	0.226797	2.084791	0.000346	0.000596
AC132219.1	0.039927	0.110522	1.468912	0.008067	0.01128
SAMSN1-AS1	0.016522	0.189492	3.519653	2.05E-07	5.65E-07
AC254629.1	0.005926	0.121317	4.355583	2.94E-13	1.81E-12
MYHAS	0.009805	0.033495	1.772381	1.63E-05	3.40E-05
AC091132.1	0.026277	0.068722	1.386988	0.0044	0.006402
LINC01738	0.11158	0.011702	-3.25324	1.66E-23	4.35E-22
AC091729.3	1.095668	2.51859	1.200806	1.51E-15	1.31E-14
AL021707.4	0.263774	0.653934	1.30984	2.23E-07	6.11E-07
EGOT	0.083127	0.585898	2.817255	2.20E-08	7.02E-08
LINC00622	0.65359	0.299062	-1.12794	1.20E-16	1.22E-15
AC092171.4	0.171464	0.701392	2.032312	1.39E-16	1.40E-15

LINC01775	0.02869	0.177928	2.632661	1.72E-13	1.10E-12
LINC01929	0.083093	0.322017	1.954336	0.00018	0.000322
AL033528.2	0.014586	0.033502	1.199628	0.000116	0.000214
AL356272.1	0.01694	0.049992	1.561231	0.035798	0.045171
LINC01460	0.028251	0.510853	4.176551	1.37E-19	2.07E-18
AC010655.2	0.108033	0.266743	1.303982	1.15E-05	2.46E-05
AC055854.1	0.133196	0.058123	-1.19637	7.57E-15	5.92E-14
CR936218.1	0.722647	1.556274	1.106732	0.00112	0.001792
MBNL1-AS1	1.593753	0.518785	-1.61922	3.18E-28	2.35E-26
PRKCA-AS1	0.004477	0.224561	5.648397	0.014898	0.019936
AP003419.2	0.163592	0.368195	1.170368	0.008696	0.012086
AC018755.4	3.649522	1.26623	-1.52717	6.04E-22	1.20E-20
AC016027.2	0.005863	0.031282	2.415657	0.000673	0.001111
LINC01354	0.326187	0.10495	-1.636	2.67E-25	1.02E-23
AC119424.1	1.296608	0.149216	-3.11926	4.25E-22	8.78E-21
Z85994.1	0.006734	0.116856	4.117231	7.84E-10	3.02E-09
AC022509.4	0.171487	0.053338	-1.68485	9.49E-10	3.59E-09
PRC1-AS1	0.03901	0.129896	1.735429	8.00E-12	4.14E-11
AC064807.2	0.03455	0.181559	2.393681	1.01E-07	2.93E-07
AL135787.1	0.027162	0.190397	2.809356	6.58E-06	1.46E-05
AP002761.3	0.694973	0.288532	-1.26822	4.34E-24	1.30E-22
AC009065.2	0.225695	3.059685	3.760933	1.36E-22	3.05E-21
AP001160.4	0.361221	0.822461	1.187064	0.000106	0.000197
AC011603.2	0.042519	0.086787	1.029387	6.46E-05	0.000124
HOXC-AS3	0.001954	0.165701	6.405927	7.25E-08	2.15E-07
AC254562.2	0.02934	0.065028	1.148187	0.003737	0.005501
AL133243.1	0.053916	0.220015	2.028824	0.000656	0.001087
AL353804.2	0.153508	0.674826	2.136204	0.004432	0.006445
AC027601.3	0.178512	0.603058	1.756275	3.97E-12	2.13E-11
AC022398.1	0.059874	0.165456	1.466446	0.004125	0.006035
AC025031.4	0.033874	0.300372	3.148481	1.93E-16	1.89E-15
AP000439.3	0.008676	0.036335	2.066244	0.004326	0.006301
AC104958.2	1.257768	4.219102	1.74607	9.11E-07	2.29E-06
AP005432.1	0.014645	0.08778	2.583433	0.009488	0.013111
LINC02398	0.045593	0.673356	3.884494	1.69E-08	5.44E-08
EDNRB-AS1	0.013014	0.058035	2.156852	0.000627	0.001042
LINC01473	0.17896	0.390471	1.125581	8.19E-05	0.000155
AF038458.2	0.026578	0.057387	1.11051	0.007512	0.010556
LINC00942	0.038718	4.061868	6.712979	8.86E-21	1.53E-19
AC022784.5	0.168319	0.535049	1.668475	5.01E-12	2.65E-11
MYOSLID	0.13775	1.578689	3.518607	3.07E-21	5.73E-20
AC024361.2	0.08935	0.196245	1.135116	4.68E-07	1.23E-06
AC013731.1	0.088577	0.478859	2.434598	5.69E-20	9.14E-19
EHMT2-AS1	0.053598	0.184047	1.779833	7.28E-09	2.46E-08

AC244205.1	0.091155	0.426148	2.224965	3.63E-10	1.45E-09
ZNF346-IT1	0.200977	0.43626	1.118163	0.000657	0.001088
AL133346.1	0.260527	0.124662	-1.06341	9.82E-10	3.71E-09
AC008752.1	0.040887	0.088183	1.108845	0.000734	0.001205
AC012213.3	0.108187	0.324679	1.585487	1.45E-08	4.70E-08
LINC01352	0.902788	0.189448	-2.25258	1.45E-26	7.16E-25
AC006273.1	1.069522	0.1904	-2.48986	5.06E-29	4.84E-27
MEG3	0.613015	2.580373	2.073586	0.009629	0.013291
AC107909.2	0.021874	0.159964	2.870477	7.27E-05	0.000138
AC084816.1	0.001647	0.026268	3.995379	1.92E-06	4.63E-06
AL358334.3	0.055269	0.135044	1.288904	6.82E-05	0.00013
AC010547.2	0.086097	0.494492	2.521915	0.000175	0.000313
LANCL1-AS1	3.83613	0.373828	-3.3592	1.23E-31	2.72E-29
MIR22HG	19.22886	6.667804	-1.52799	2.53E-28	1.99E-26
AL391244.3	0.502701	1.755558	1.804157	3.95E-24	1.21E-22
AC022098.2	0.18497	1.392533	2.91235	2.85E-07	7.70E-07
SNHG17	2.457714	5.356457	1.123962	1.65E-16	1.63E-15
C10orf91	0.069476	0.347798	2.32367	2.88E-13	1.78E-12
LINC01096	0.007704	0.064795	3.072274	0.000231	0.000408
AC027601.2	0.723801	0.20454	-1.82321	2.50E-23	6.30E-22
AC009237.14	1.647207	4.454622	1.435281	1.29E-23	3.42E-22
FLJ45513	0.314024	1.106196	1.816659	2.39E-10	9.87E-10
AC027514.2	0.160508	0.399171	1.31436	0.008384	0.011671
AP003119.2	0.227453	0.936792	2.042159	3.29E-12	1.78E-11
EP300-AS1	4.042441	1.362172	-1.56932	2.65E-25	1.02E-23
AC100778.3	0.071657	0.145825	1.025058	5.27E-05	0.000102
AL157373.2	0.10737	0.507149	2.239817	1.48E-07	4.18E-07
AP003559.1	0.036575	0.485821	3.731482	1.03E-23	2.80E-22
AP003472.1	0.025652	0.165448	2.689223	8.15E-10	3.12E-09
AC012073.1	0.458314	1.266735	1.466708	1.19E-18	1.61E-17
HSPC324	6.405087	0.507818	-3.65683	2.68E-31	5.27E-29
LINC01267	0.407858	0.134156	-1.60416	3.42E-06	7.85E-06
AC139720.1	0.062368	0.239855	1.943292	7.18E-05	0.000137
AC011921.1	0.148957	0.375174	1.332665	1.35E-10	5.81E-10
AC138356.1	0.174659	0.085048	-1.03819	4.11E-21	7.49E-20
PVT1	0.412965	3.060577	2.889712	3.73E-30	4.52E-28
LINC00626	0.006962	0.121917	4.130215	1.86E-12	1.05E-11
AC034229.4	0.133906	0.462481	1.788175	2.26E-08	7.18E-08
AL117328.2	0.00484	0.017423	1.847986	2.08E-05	4.27E-05
AC015849.5	0.007655	0.111058	3.858764	3.74E-23	9.24E-22
AL031848.1	0.054789	0.171922	1.649787	3.32E-06	7.66E-06
AC027243.1	0.007586	0.05942	2.969543	5.22E-07	1.36E-06
AC005856.1	1.553548	0.529211	-1.55365	2.21E-25	8.79E-24
AL162632.1	0.022736	0.059828	1.395857	0.000172	0.000309

AL135786.2	0.15817	0.058332	-1.43911	8.31E-17	8.68E-16
FLJ12825	0.022715	0.104177	2.197328	2.47E-11	1.20E-10
AL662860.1	0.351725	0.131852	-1.41553	5.59E-07	1.45E-06
AC253576.2	0.290266	0.906854	1.643493	2.21E-06	5.24E-06
LINC01105	0.172142	0.068225	-1.33523	8.70E-18	1.05E-16
AC084048.1	0.02719	0.094433	1.796207	6.68E-07	1.71E-06
AC090912.3	0.053618	0.145501	1.440238	0.00666	0.009427
AC007342.5	0.318582	1.097272	1.784185	2.98E-14	2.14E-13
AC079921.1	0.174297	0.423521	1.280889	9.10E-06	1.98E-05
CERNA3	0.010956	0.070003	2.67575	6.08E-08	1.82E-07
AL022313.2	0.110438	0.879089	2.992774	1.52E-05	3.19E-05
AC020594.1	0.25725	0.648358	1.333619	1.44E-08	4.68E-08
AL590006.1	0.027042	0.211075	2.964488	1.25E-06	3.09E-06
LINC01948	0.146152	0.337587	1.20779	1.42E-05	3.00E-05
AC116407.1	5.224849	1.452359	-1.84699	5.11E-27	2.74E-25
AC016737.2	0.057658	0.179787	1.64069	0.001243	0.001977
AC131009.3	0.601984	1.668518	1.470769	1.97E-16	1.93E-15
LINP1	0.073101	1.345486	4.202093	3.89E-07	1.03E-06
AC009704.2	0.14699	0.459724	1.645045	1.14E-05	2.45E-05
LINC00582	0.142651	0.610775	2.098151	5.45E-11	2.49E-10
AC010327.3	0.008407	0.036866	2.132578	2.51E-05	5.10E-05
AC108676.1	0.034479	0.26292	2.930813	1.38E-12	7.84E-12
AC007879.4	0.052354	0.176865	1.756263	2.70E-07	7.34E-07
AL031058.1	0.415543	1.949259	2.229855	8.72E-17	9.05E-16
AP000844.2	0.122541	0.438331	1.838752	0.024197	0.031367
AC245123.1	0.019416	0.003866	-2.32827	1.77E-11	8.71E-11
AC005224.1	0.112992	0.229848	1.024466	1.44E-10	6.14E-10
LINC01908	0.510097	0.156805	-1.7018	1.15E-09	4.31E-09
AC090061.1	0.108242	0.217044	1.003719	4.99E-06	1.12E-05
AC093620.1	0.239327	0.480087	1.004316	0.000556	0.000933
AC027117.2	0.344301	1.349831	1.971035	0.000436	0.000741
AC116312.1	0.701803	0.100127	-2.80923	9.66E-30	1.03E-27
PITPNA-AS1	2.509244	5.216288	1.055771	6.20E-14	4.22E-13
AC104984.4	4.000637	0.269485	-3.89195	1.00E-31	2.30E-29
AL139130.1	0.002511	0.012807	2.350566	1.62E-06	3.93E-06
LINC01719	0.207049	0.668125	1.690147	2.88E-18	3.70E-17
AC016027.1	0.128852	0.326498	1.34136	4.40E-21	7.97E-20
AL031600.1	0.418961	1.274474	1.605014	3.46E-11	1.63E-10
FGF14-AS2	2.908657	1.060732	-1.45529	1.40E-24	4.59E-23
ARNTL2-AS1	0.008115	0.116262	3.840663	2.88E-08	9.03E-08
C2orf48	0.042191	0.368544	3.126827	2.69E-22	5.79E-21
AL034397.3	2.747314	1.039021	-1.4028	1.33E-18	1.78E-17
LINC01436	0.890804	9.146822	3.36009	3.46E-07	9.24E-07
AC017083.1	0.079318	0.34532	2.122219	1.82E-17	2.10E-16

FAM222A-AS1	0.054469	0.379987	2.80244	5.65E-12	2.98E-11
AP000873.3	0.022097	0.068033	1.622385	1.26E-05	2.70E-05
AC117386.2	0.0064	0.241118	5.235627	7.09E-11	3.17E-10
AC106782.6	0.707188	1.502879	1.087563	1.34E-10	5.80E-10
AL356752.1	0.064935	0.275262	2.083734	0.00327	0.004864
AL591686.1	0.399729	0.061544	-2.69933	2.37E-22	5.14E-21
DGCR10	0.091992	0.444082	2.271245	8.81E-08	2.58E-07
LINC00525	0.028132	0.362792	3.688876	2.88E-18	3.70E-17
AC009137.2	0.050551	0.185399	1.874826	9.46E-05	0.000177
AP000692.1	0.331748	0.697178	1.071439	1.01E-07	2.92E-07
AL133551.1	0.030806	0.125275	2.023807	4.38E-15	3.55E-14
AC114684.1	0.123375	0.05837	-1.07976	1.35E-05	2.85E-05
LINC01028	0.122006	0.007625	-4.00007	3.14E-18	4.01E-17
LINC02598	0.038649	0.18068	2.224924	5.95E-05	0.000115
FEZF1-AS1	0.03221	2.669751	6.373059	2.19E-25	8.79E-24
AC102953.2	0.529966	1.248519	1.236248	1.03E-05	2.23E-05
PLCB2-AS1	0.016974	0.056988	1.747351	2.05E-05	4.23E-05
AC011773.1	0.04376	0.197337	2.172963	4.32E-08	1.33E-07
AC005726.5	0.017777	0.044559	1.325743	0.000185	0.000331
AL354707.1	0.089787	0.809551	3.172543	2.42E-20	3.96E-19
SALRNA1	0.025996	0.232936	3.163567	2.89E-15	2.42E-14
AL355974.3	0.085408	0.031671	-1.43121	2.37E-08	7.50E-08
LINC01963	3.183377	1.506741	-1.07913	3.43E-21	6.37E-20
AL355596.1	0.00505	0.264428	5.710589	3.10E-09	1.09E-08
AP003555.2	0.120099	0.392507	1.708487	0.037214	0.046853
NALCN-AS1	0.222889	0.071564	-1.63902	9.64E-16	8.47E-15
AC084030.1	0.26288	0.01179	-4.47874	7.05E-37	5.48E-34
AL606489.1	0.250049	0.990132	1.985409	2.72E-13	1.68E-12
AP000866.2	2.004413	0.829359	-1.27311	1.13E-20	1.93E-19
AC016683.1	0.025308	0.083426	1.720909	0.0138	0.018594
AC069544.1	0.288058	0.838603	1.541631	1.23E-17	1.45E-16
AC107021.2	0.162686	0.459662	1.498483	0.000766	0.001255
AL355304.1	0.626148	0.148596	-2.07511	5.44E-07	1.41E-06
AC087269.1	0.037976	0.666746	4.133978	1.46E-13	9.37E-13
AL158042.1	0.052123	0.023166	-1.16989	6.00E-06	1.34E-05
LINC01095	0.007591	0.003413	-1.15319	2.46E-07	6.71E-07
LINC01638	0.10556	0.219814	1.058225	6.30E-08	1.88E-07
AL136982.1	0.065115	0.020509	-1.66673	1.44E-16	1.44E-15
AL033397.2	0.061869	1.308015	4.402009	1.56E-16	1.56E-15
LINC00491	0.007492	0.503928	6.071693	8.12E-07	2.06E-06
AL928742.1	0.19532	0.589424	1.593467	2.84E-09	1.00E-08
MEIS1-AS3	0.02255	0.06842	1.601295	0.0097	0.013383
AL442125.1	0.179194	0.417836	1.221414	3.87E-05	7.64E-05
AL133264.2	0.297669	0.131944	-1.17378	4.84E-12	2.58E-11

AL138900.3	1.925693	0.622712	-1.62874	2.43E-17	2.73E-16
AC113404.1	0.319896	0.053401	-2.58266	0.013647	0.018404
LINC01703	0.165389	0.968299	2.549589	7.33E-27	3.80E-25
IGFL2-AS1	0.015964	0.31503	4.302586	5.91E-08	1.77E-07
AL355810.1	0.021912	0.07422	1.760073	0.004388	0.006389
AL132712.2	0.571039	2.21559	1.956029	4.91E-19	7.08E-18
AP001107.9	3.681754	1.628897	-1.1765	3.73E-22	7.78E-21
AC026250.1	0.070577	0.325409	2.204992	5.76E-24	1.67E-22
LINC02324	0.007355	0.06718	3.191181	2.58E-13	1.60E-12
LINC01711	0.019198	0.335967	4.129299	6.71E-24	1.87E-22
LINC00670	0.039401	0.013792	-1.51437	1.56E-18	2.07E-17
AL109613.1	0.308905	1.312019	2.086553	1.15E-13	7.45E-13
AC116345.1	0.007839	0.335284	5.418509	9.00E-06	1.96E-05
LINC01708	0.154216	0.074919	-1.04154	2.76E-05	5.56E-05
AL359697.1	0.24188	0.554689	1.19739	0.006621	0.009381
AL078587.1	0.058344	0.429022	2.878402	4.52E-16	4.17E-15
LINC00211	0.089613	0.020268	-2.14447	1.21E-24	4.04E-23
AC104534.1	0.403626	0.834928	1.048631	0.033108	0.042023
AC079160.1	0.001267	0.14683	6.857077	1.33E-11	6.63E-11
ARLNC1	0.031243	0.143365	2.198071	0.000282	0.000491
AC011481.1	0.206987	0.587584	1.505254	1.94E-11	9.54E-11
EDRF1-AS1	0.092947	0.202162	1.121025	0.00916	0.012689
LINC02163	0.004409	0.518183	6.876983	8.96E-11	3.96E-10
MEF2C-AS2	0.056477	0.011365	-2.31307	3.60E-13	2.19E-12
AL391422.2	0.14283	0.050185	-1.50896	6.89E-07	1.76E-06
RNF144A-AS1	0.081481	0.401076	2.299343	3.55E-13	2.16E-12
LINC00265	0.80757	1.748608	1.114547	3.16E-11	1.50E-10
BEAN1-AS1	0.068918	0.167214	1.278741	0.001899	0.002933
AC090772.3	0.265688	0.855552	1.687124	3.38E-06	7.78E-06
AC098869.2	0.221952	0.510361	1.201269	4.61E-05	9.03E-05
LINC02313	0.021145	0.530248	4.648308	3.89E-12	2.09E-11
AC005670.1	0.029907	0.12855	2.103769	2.92E-10	1.19E-09
AC084026.1	0.008442	0.101667	3.590197	1.24E-07	3.54E-07
LINC00698	0.00125	0.013947	3.479577	5.10E-08	1.54E-07
AC010931.2	0.081043	0.165539	1.030419	3.37E-05	6.69E-05
STK32A-AS1	0.137227	1.516428	3.466045	4.89E-17	5.29E-16
AL139125.1	0.025318	0.097513	1.945404	1.86E-07	5.17E-07
AC097347.1	0.040782	0.173886	2.092147	7.58E-07	1.93E-06
AC023794.4	0.039342	0.123373	1.6489	0.003593	0.005311
AC117402.1	0.088729	0.727246	3.034973	0.004092	0.00599
AP003354.1	0.057433	0.206043	1.84298	0.00102	0.001643
AC068722.2	0.549139	0.241692	-1.184	3.53E-11	1.66E-10
LINC01447	0.142433	0.020062	-2.82773	4.54E-28	3.17E-26
AC236972.3	2.806766	0.555268	-2.33765	3.46E-27	1.96E-25

AC003965.1	0.194545	0.420669	1.11258	9.84E-07	2.47E-06
AC037487.1	0.025186	0.147089	2.545995	3.34E-07	8.93E-07
AC100791.2	0.004159	0.082908	4.31723	1.68E-10	7.09E-10
AC004771.2	0.071628	0.227926	1.669971	4.62E-08	1.41E-07
AC135782.1	0.002976	0.043576	3.872038	1.44E-07	4.06E-07
AC008406.3	0.002795	0.03765	3.751692	8.73E-10	3.33E-09
COL4A2-AS1	0.370293	0.156594	-1.24164	8.17E-10	3.13E-09
AL356489.2	0.163521	0.070829	-1.20706	1.13E-17	1.35E-16
AC092834.1	0.550096	0.075828	-2.85889	7.94E-16	7.12E-15
AC025166.1	0.043234	0.355797	3.040805	1.08E-13	7.09E-13
AC083843.1	0.018918	0.050485	1.416085	0.000298	0.000516
AC005884.1	0.829213	0.204816	-2.01742	7.55E-24	2.09E-22
AC027307.3	0.529309	0.228806	-1.20999	1.68E-21	3.20E-20
AC092535.4	1.229268	3.383566	1.460744	9.22E-06	2.00E-05
AL022344.2	0.248674	0.103549	-1.26395	3.02E-14	2.17E-13
AC105914.2	1.573797	0.245502	-2.68044	9.65E-29	8.69E-27
VWA8-AS1	0.190721	0.066119	-1.52832	7.81E-09	2.63E-08
AC004990.1	0.107058	0.333416	1.638935	0.027131	0.034879
AC231981.1	0.164192	0.357097	1.120929	8.96E-09	2.99E-08
AL354993.2	0.034325	0.414365	3.59359	3.12E-07	8.38E-07
AC095050.1	0.795575	0.03636	-4.45157	8.60E-38	8.91E-35
AL158835.1	0.015451	0.05777	1.90263	7.56E-11	3.36E-10
AL121917.1	0.186186	0.438958	1.237334	1.21E-10	5.24E-10
MYO16-AS1	4.29599	0.776684	-2.46759	2.42E-25	9.36E-24
AC004596.1	0.315594	0.647221	1.036186	8.35E-12	4.30E-11
AC040169.3	0.204072	0.490429	1.264962	3.50E-08	1.09E-07
AC016877.3	0.054456	0.583717	3.422101	1.82E-16	1.79E-15
F11-AS1	0.464176	0.108142	-2.10175	4.49E-23	1.09E-21
LINC01515	0.069727	0.287368	2.043108	6.19E-17	6.60E-16
AC069503.1	0.011182	0.046011	2.040814	2.43E-07	6.63E-07
AC009065.6	0.015093	0.075941	2.331022	4.00E-07	1.06E-06
AC114488.1	0.105248	0.52896	2.329367	3.16E-10	1.28E-09
AL049612.1	0.053109	0.123818	1.221192	0.000929	0.001503
AC084117.1	0.589365	1.316996	1.160017	5.54E-05	0.000107
AC005534.1	0.107282	0.31331	1.546176	1.11E-06	2.76E-06
AL353616.2	0.041183	0.011914	-1.78934	1.25E-07	3.56E-07
AC004921.1	1.509821	0.570327	-1.40452	7.29E-21	1.28E-19
AC021321.1	0.150455	0.392419	1.383063	4.74E-10	1.87E-09
SRRM2-AS1	0.096657	0.236554	1.291228	3.36E-11	1.59E-10
CASC11	0.008398	0.025443	1.599217	6.14E-07	1.58E-06
AC023794.2	0.018277	0.133731	2.871261	1.99E-06	4.78E-06
AL353747.2	0.563685	0.190827	-1.56262	6.20E-07	1.59E-06
FGF12-AS2	0.014083	0.115088	3.030699	3.22E-05	6.40E-05
AC106028.4	0.701393	0.336154	-1.0611	2.14E-05	4.40E-05

JAZF1-AS1	0.038587	0.01791	-1.10733	7.91E-13	4.64E-12
AL121899.1	1.375161	0.587228	-1.22761	6.68E-05	0.000128
AL096677.1	0.040289	0.080687	1.001937	0.000309	0.000535
AC244034.2	0.098236	0.215683	1.134581	0.000229	0.000405
LINC02100	0.308895	0.887874	1.523237	1.55E-05	3.25E-05
AC040904.1	0.071924	0.50014	2.797781	1.84E-08	5.93E-08
LINC01968	0.002017	0.016284	3.013445	1.24E-11	6.24E-11
AC093752.2	0.029914	0.146899	2.295922	6.41E-07	1.64E-06
LINC00323	0.055693	0.130943	1.233373	0.024696	0.031939
AC005291.1	0.022926	0.195711	3.09368	2.63E-06	6.18E-06
TESC-AS1	0.016092	0.093183	2.533739	0.010025	0.013783
LINC01214	0.012156	1.308456	6.750031	4.33E-11	2.01E-10
LINC02468	0.030265	0.230401	2.928432	0.000127	0.000233
ALMS1-IT1	0.226164	0.609572	1.430425	1.51E-09	5.57E-09
AC004906.1	0.01892	0.070395	1.895534	6.40E-06	1.42E-05
AC026310.2	0.027101	0.09309	1.780276	0.00011	0.000205
LINC01126	0.104051	0.271319	1.38269	2.47E-07	6.72E-07
AC004221.1	0.009646	0.10318	3.419088	1.28E-16	1.29E-15
LINC02198	0.26951	0.542887	1.01031	0.000747	0.001225
THCAT158	0.259013	0.648741	1.32462	0.000176	0.000316
AL022097.1	0.032339	0.244653	2.919387	0.004253	0.006204
AC025176.1	0.072941	0.339306	2.217778	4.85E-12	2.58E-11
AC007569.1	0.14252	0.397462	1.479648	1.40E-07	3.97E-07
LINC01526	0.222054	0.096899	-1.19636	1.81E-09	6.60E-09
AC006058.1	0.833776	0.382918	-1.12263	1.27E-13	8.20E-13
AC090912.2	0.042315	0.154181	1.865374	4.53E-09	1.57E-08
AC009171.2	0.268032	0.62256	1.215809	1.71E-09	6.24E-09
KLHL7-DT	0.140096	0.542922	1.954324	3.11E-13	1.91E-12
AL512604.3	0.009752	0.037125	1.928663	1.29E-05	2.74E-05
AL353770.4	0.256268	0.057277	-2.16162	5.33E-14	3.68E-13
AC004816.1	0.772865	6.48718	3.069303	2.71E-31	5.27E-29
AC016708.1	0.10806	0.436084	2.012773	1.81E-14	1.34E-13
LACTB2-AS1	0.051477	0.226863	2.139819	1.80E-13	1.14E-12
LINC01614	0.217566	3.299927	3.922911	6.12E-24	1.74E-22
AP006545.2	0.082556	0.270936	1.714514	6.97E-08	2.07E-07
AC010973.2	0.424873	0.953086	1.165575	1.42E-08	4.61E-08
AC109446.3	0.190717	1.031489	2.435225	1.10E-06	2.74E-06
AC092794.2	0.042439	0.184219	2.117965	0.000407	0.000694
AC068025.2	0.036293	0.245436	2.757597	0.003499	0.005186
AL157838.1	0.139752	0.901301	2.689139	4.26E-24	1.28E-22
AC053503.4	0.190311	0.058188	-1.70955	4.60E-11	2.13E-10
AC078778.1	0.182177	0.854998	2.230576	5.61E-17	6.01E-16
LINC00511	0.320201	3.054288	3.253787	1.21E-28	1.05E-26
LINC02156	0.015644	0.259131	4.050017	8.03E-15	6.22E-14

AL133355.1	4.680814	2.019931	-1.21245	5.82E-24	1.67E-22
AC073641.1	0.034809	0.12914	1.891393	2.27E-09	8.11E-09
AF230666.2	0.022695	0.150652	2.730747	4.79E-08	1.46E-07
AC004461.2	0.122141	0.28797	1.237368	0.006718	0.009503
AC113346.1	0.010891	0.405659	5.219003	5.41E-16	4.95E-15
AC093585.1	0.083263	0.274605	1.721607	0.003572	0.005283
AC009097.1	0.028235	0.058375	1.047894	0.003762	0.005538
AC109479.1	0.016538	0.057902	1.807785	4.36E-05	8.58E-05
AL645608.1	0.103984	0.354336	1.768761	1.89E-10	7.94E-10
AC068858.1	0.004554	0.025256	2.471571	1.67E-07	4.66E-07
LINC00638	0.499552	1.131614	1.179676	3.38E-11	1.59E-10
LINC02552	0.041795	0.008404	-2.31411	1.52E-17	1.77E-16
AC010524.1	0.128153	0.270758	1.079135	0.004068	0.005959
AC024560.1	0.023082	0.150497	2.704892	2.39E-09	8.51E-09
LINC00629	0.018769	0.245531	3.709498	4.81E-19	6.95E-18
AC090826.1	0.009219	0.042706	2.211805	1.70E-05	3.55E-05
AL157400.2	0.005201	0.076919	3.886394	1.36E-12	7.73E-12
AL591441.1	0.016684	0.007256	-1.20125	2.71E-06	6.34E-06
AC128709.2	0.395943	0.087947	-2.17058	1.19E-28	1.04E-26
AC009121.2	0.053104	0.204555	1.945608	1.34E-15	1.16E-14
AL024498.1	0.124508	0.376193	1.595239	1.05E-19	1.63E-18
MORC2-AS1	0.0572	0.115583	1.014831	1.82E-05	3.77E-05
AC004112.1	0.670723	0.262763	-1.35196	1.61E-21	3.09E-20
AC020661.3	0.043893	0.254824	2.537435	0.032545	0.041351
AL157823.2	0.072883	0.22938	1.654077	4.03E-06	9.20E-06
AC104237.2	0.794452	0.073345	-3.43719	8.19E-36	5.66E-33
AL928970.1	0.004455	0.013085	1.554277	0.000679	0.001119
AL136369.1	2.242598	0.290847	-2.94684	8.88E-33	3.25E-30
MGC12916	0.192979	0.445344	1.206473	7.76E-06	1.70E-05
AL358115.1	0.035561	0.191911	2.432082	3.55E-11	1.67E-10
LINC02539	0.020603	0.077204	1.905822	1.16E-05	2.49E-05
AC087683.2	0.164291	0.447256	1.444842	0.005327	0.007666
CAMTA1-DT	0.227091	0.527714	1.216484	2.05E-08	6.56E-08
AP002907.1	0.26495	0.899346	1.763157	9.12E-12	4.67E-11
U52111.1	0.12166	0.648066	2.413286	5.83E-19	8.31E-18
AC124854.1	0.169836	0.076853	-1.14397	3.31E-16	3.14E-15
AC048337.1	0.024075	0.080654	1.744226	1.36E-06	3.32E-06
AP002807.1	0.711986	1.501893	1.07686	4.03E-07	1.06E-06
LINC00313	0.016248	0.039679	1.28807	0.007205	0.01015
AC127024.3	0.077492	0.384961	2.31259	4.09E-06	9.33E-06
LINC01842	0.056182	0.720542	3.680907	9.27E-14	6.12E-13
AC127024.2	0.081804	0.243319	1.572606	2.14E-06	5.10E-06
LINC02006	0.301668	0.129395	-1.22119	3.96E-11	1.85E-10
AC092155.1	0.037046	0.012248	-1.59673	3.83E-10	1.53E-09

THRA1/BTR	0.005688	0.286595	5.654863	1.08E-11	5.49E-11
AC011479.2	0.141935	0.325749	1.198527	6.04E-05	0.000116
AC044810.3	0.042719	0.002946	-3.85827	5.89E-28	3.94E-26
AL138720.1	0.015199	0.078022	2.359948	1.93E-10	8.06E-10
LINC00551	1.467295	0.179066	-3.03459	4.35E-16	4.02E-15
AC020978.1	0.064296	0.172953	1.427575	0.000515	0.000868
KCTD21-AS1	0.221736	0.49448	1.15707	7.81E-13	4.59E-12
AC084064.1	0.089697	0.027319	-1.71513	3.09E-15	2.57E-14
AC120498.4	0.01155	1.01533	6.457899	3.83E-16	3.60E-15
LINC02014	0.085765	1.436347	4.065865	2.62E-30	3.55E-28
AC009148.1	0.036044	0.168147	2.221878	9.12E-16	8.10E-15
AC019155.3	0.013284	0.615838	5.534783	1.43E-07	4.04E-07
AL133215.2	0.091719	0.4396	2.260901	4.56E-26	2.04E-24
AC106779.1	0.014318	0.058445	2.02926	5.75E-11	2.62E-10
AL353801.3	0.087788	0.29728	1.759732	2.14E-10	8.90E-10
AC004158.1	0.00084	0.018327	4.447693	1.92E-06	4.63E-06
AP003086.2	0.080328	0.258004	1.683423	0.000126	0.000232
AF131215.7	0.724851	0.170668	-2.0865	5.31E-26	2.31E-24
AL928654.1	0.440701	1.102225	1.322548	4.43E-15	3.58E-14
AC110285.6	0.095554	0.517061	2.435954	4.39E-13	2.65E-12
AL162414.1	0.048496	0.132832	1.453651	0.00572	0.008175
AL449106.1	0.079014	0.187617	1.247606	1.96E-12	1.09E-11
AL023803.2	0.077287	0.249727	1.692046	2.30E-12	1.27E-11
MAFG-DT	1.513366	3.836775	1.342133	1.65E-17	1.91E-16
AC024257.3	0.109971	0.3473	1.659055	1.30E-10	5.63E-10
AL031123.2	0.157884	0.45483	1.526461	4.51E-13	2.72E-12
LINC01547	0.654506	2.151546	1.716895	6.61E-15	5.23E-14
AC098828.3	0.029549	0.109315	1.887312	0.006013	0.008567
AL592164.1	0.010589	0.030386	1.520896	9.74E-05	0.000182
AL731684.1	0.008475	0.256416	4.919069	2.88E-12	1.57E-11
AL357992.1	0.173912	0.620673	1.835473	1.63E-08	5.28E-08
MIR2117HG	0.022393	0.087859	1.972138	0.000282	0.00049
SYNPR-AS1	0.400518	1.755819	2.132206	1.70E-10	7.18E-10
LINC02601	0.063435	0.134194	1.080966	3.08E-05	6.15E-05
AC068051.1	0.028211	0.094923	1.750504	0.007704	0.0108
AC006441.1	0.168679	0.4037	1.259001	0.002606	0.003935
LINC01836	3.771993	1.286343	-1.55205	6.32E-19	8.97E-18
AL391807.1	0.235263	0.097695	-1.26791	9.53E-17	9.83E-16
WWC2-AS2	0.609505	0.213501	-1.5134	2.52E-23	6.32E-22
TRIM31-AS1	0.226158	1.218547	2.429759	2.00E-15	1.70E-14
AC074117.1	0.78352	1.841647	1.232955	4.96E-19	7.12E-18
AL022341.2	0.034985	0.164096	2.229745	3.80E-08	1.17E-07
AP000977.1	0.007762	0.054573	2.813746	5.14E-11	2.37E-10
AL137796.1	0.039605	0.088467	1.159447	0.001122	0.001794

AC012531.1	0.007047	0.107937	3.937088	5.95E-08	1.79E-07
AL450992.2	2.53578	10.79013	2.08921	1.40E-20	2.35E-19
LGALS8-AS1	0.088438	0.255497	1.530568	3.09E-10	1.25E-09
AP002498.1	0.110561	2.008066	4.182895	3.90E-16	3.66E-15
AC092828.1	0.034723	0.290301	3.0636	4.88E-05	9.54E-05
AC013457.1	5.148034	0.625563	-3.04079	7.81E-19	1.09E-17
AC005532.1	0.047705	0.187725	1.976403	1.14E-05	2.45E-05
AC016773.1	0.208586	0.897461	2.105208	7.69E-20	1.21E-18
AC009509.4	0.655946	0.2732	-1.26362	2.21E-17	2.50E-16
SATB1-AS1	0.028001	0.074004	1.402108	2.33E-06	5.50E-06
LINC02284	0.083232	0.03739	-1.15448	5.14E-11	2.37E-10
LINC00896	0.040517	0.440741	3.443345	3.21E-22	6.86E-21
AC112484.3	0.197052	0.506917	1.363169	0.000418	0.000712
AC136475.3	3.57386	15.99053	2.161663	1.48E-06	3.61E-06
LINC02416	0.042084	0.889525	4.401699	1.68E-26	8.03E-25
AC048382.1	0.041418	0.111873	1.433544	9.43E-08	2.75E-07
AL359636.2	0.043075	0.29442	2.772967	1.08E-05	2.33E-05
ATP2B1-AS1	0.794382	0.389715	-1.02741	1.16E-19	1.78E-18
AC138696.2	0.76965	1.760374	1.193607	4.54E-11	2.10E-10
AC027796.1	0.081912	0.285297	1.800315	4.72E-06	1.07E-05
AF127936.2	0.017664	0.170286	3.26907	2.12E-17	2.41E-16
AL158211.1	0.451509	0.170124	-1.40817	3.06E-21	5.73E-20
AC135012.2	0.04958	0.015998	-1.63182	4.84E-08	1.47E-07
LINC01485	0.021464	0.110152	2.359502	5.00E-08	1.52E-07
AC003991.2	0.089503	0.215223	1.265824	0.009572	0.013219
Z97055.2	0.079385	0.166761	1.070846	5.68E-07	1.47E-06
AP000786.1	0.320716	0.839721	1.388613	0.000195	0.000349
AL445426.1	0.366746	0.12107	-1.59894	1.21E-18	1.64E-17
AC078905.1	0.167092	0.026457	-2.6589	2.80E-24	8.66E-23
AC103706.1	0.540772	1.387889	1.359799	1.37E-08	4.47E-08
AC012625.1	0.03324	0.144271	2.117772	0.00082	0.001339
AL391427.1	0.122071	1.962976	4.007253	6.54E-07	1.67E-06
LINC01559	0.007321	0.646746	6.464994	1.11E-12	6.40E-12
AL109615.3	0.248236	2.292623	3.207217	3.59E-17	3.96E-16
ELFN1-AS1	0.058779	0.909911	3.952359	3.18E-10	1.29E-09
AL079307.1	0.001703	0.015926	3.225451	9.00E-06	1.96E-05
LINC02600	0.240293	0.114822	-1.0654	1.18E-20	1.99E-19
AC027627.1	0.001196	0.078265	6.031563	1.04E-10	4.57E-10
AC004832.5	0.175827	0.534942	1.605229	1.57E-05	3.28E-05
AC025524.2	0.002875	0.023097	3.00592	1.97E-06	4.73E-06
CA3-AS1	0.532804	0.25434	-1.06685	7.90E-18	9.63E-17
SLC9A3-AS1	0.791059	2.822462	1.835096	2.10E-10	8.75E-10
AC007750.1	0.022378	0.120875	2.433356	8.56E-19	1.19E-17
AC109322.1	0.406108	1.101105	1.439016	2.83E-19	4.18E-18

AP006621.2	0.726461	1.729214	1.251159	3.07E-05	6.13E-05
LINC02321	0.104981	0.748217	2.833322	7.59E-14	5.09E-13
AC009163.3	0.050788	0.173881	1.775534	0.002101	0.00322
AC022973.4	0.187768	0.464384	1.306368	1.31E-07	3.72E-07
AC211433.1	0.037694	0.426685	3.500752	1.13E-07	3.25E-07
AL031710.1	0.031692	0.159134	2.328031	0.003677	0.005424
AC104938.1	0.466225	0.181729	-1.35924	4.63E-21	8.32E-20
AC097468.1	0.081923	0.342979	2.065781	4.57E-10	1.81E-09
AC015921.1	0.026474	0.255773	3.272241	3.10E-25	1.17E-23
LINC00471	0.176338	0.446832	1.341387	4.24E-15	3.45E-14
AL160313.1	0.143427	0.05919	-1.27691	1.52E-13	9.74E-13
CTD-3080P12.3	0.330936	0.08464	-1.96714	0.002678	0.00404
LINC01106	0.293074	0.782188	1.416252	3.98E-13	2.41E-12
AC096536.1	0.056878	0.19584	1.783737	1.08E-06	2.68E-06
AC120114.1	0.127253	0.461346	1.858146	4.49E-21	8.10E-20
AC110285.3	0.146517	0.418063	1.512648	0.000162	0.000292
MIR503HG	0.344965	1.281536	1.893351	1.88E-11	9.22E-11
AP001453.3	0.665146	1.426662	1.1009	4.33E-15	3.52E-14
AC104260.1	0.134093	0.034971	-1.939	1.89E-18	2.50E-17
LINC00336	0.039722	0.105159	1.404554	0.000921	0.001492
LINC01012	0.057933	0.256868	2.148572	5.00E-19	7.16E-18
LINC01081	0.359039	0.038285	-3.22931	2.73E-32	8.08E-30
LINC00930	0.842237	0.40827	-1.0447	4.64E-14	3.23E-13
AL121832.2	0.969335	2.37045	1.290094	4.45E-10	1.77E-09
AL139397.1	0.085827	0.222847	1.376554	2.00E-09	7.22E-09
AC133644.2	0.153834	0.696658	2.179077	2.40E-13	1.49E-12
AC020928.2	0.007698	0.025512	1.728657	0.002054	0.003155
AC239584.1	0.011311	0.264913	4.549749	5.42E-07	1.41E-06
AC020911.1	0.089909	0.191706	1.092365	2.20E-07	6.06E-07
AC131011.1	0.147024	0.575136	1.96785	2.43E-14	1.78E-13
AC111170.1	0.037706	0.17217	2.190977	0.009321	0.0129
AC068189.1	0.008184	0.086656	3.404366	7.86E-14	5.24E-13
AC104561.1	0.052513	0.141953	1.434663	1.19E-08	3.90E-08
AC087379.1	0.006201	0.151507	4.610799	7.56E-08	2.24E-07
AL590666.2	0.199338	2.812761	3.8187	6.04E-16	5.49E-15
AL109811.2	0.273128	0.670926	1.296576	1.26E-14	9.48E-14
MIAT	0.574489	2.241792	1.964301	6.94E-07	1.77E-06
AC092535.1	0.026293	0.240887	3.195601	5.51E-07	1.43E-06
LINC02265	0.667461	0.180946	-1.88313	9.94E-08	2.88E-07
AL669831.5	0.070747	0.260248	1.879144	3.54E-14	2.52E-13
LINC02029	0.005476	0.125275	4.515811	4.29E-14	2.99E-13
AC091057.4	0.063814	0.487502	2.933467	8.82E-21	1.53E-19
AP001347.1	0.10704	0.23833	1.154803	2.44E-07	6.67E-07
AC079336.1	0.030864	0.099605	1.690286	0.000184	0.000328

AC007128.1	0.009568	0.55845	5.867109	9.15E-21	1.57E-19
FIRRE	0.060169	0.381551	2.66479	1.78E-08	5.73E-08
AL513314.2	0.072846	0.191626	1.395373	9.58E-09	3.18E-08
UCA1	0.052673	2.875255	5.770493	1.13E-11	5.70E-11
AL606970.3	0.005652	0.067189	3.571435	0.000876	0.001424
AC093390.1	0.038034	0.009714	-1.96909	1.51E-17	1.76E-16
AC024901.1	0.064588	0.549154	3.087878	4.53E-07	1.19E-06
AC105001.1	0.399931	0.152431	-1.39159	7.66E-17	8.04E-16
AC108860.2	0.634879	1.359649	1.09868	1.80E-10	7.55E-10
AC087289.1	0.079458	0.188198	1.243989	1.27E-07	3.62E-07
ADIRF-AS1	2.09611	1.040745	-1.0101	1.54E-15	1.33E-14
LINC01996	3.138186	0.148401	-4.40236	2.92E-33	1.13E-30
AC010761.4	0.12598	0.446732	1.826208	7.96E-15	6.18E-14
AL591368.1	0.012802	0.033061	1.368699	0.000354	0.000608
LINC01561	0.008408	0.092667	3.462201	5.18E-13	3.10E-12
AC015853.1	0.052278	0.18538	1.826215	5.36E-08	1.62E-07
AP000251.1	0.035771	0.479385	3.744322	2.44E-15	2.07E-14
AC024884.2	0.028358	0.082991	1.549188	4.90E-07	1.28E-06
LINC00867	0.012113	0.074761	2.62578	0.025801	0.0333
LINC01237	0.117199	0.282643	1.270018	5.57E-07	1.44E-06
AL357093.2	4.514498	1.354025	-1.73731	2.97E-10	1.21E-09
AL133153.2	0.037496	0.286686	2.934655	3.82E-19	5.59E-18
AP003385.3	0.089129	0.042036	-1.08426	7.38E-08	2.19E-07
LINC00184	0.025625	0.124431	2.279741	2.68E-07	7.29E-07
AC002310.2	0.010193	0.02656	1.381658	7.82E-05	0.000148
AC011005.4	0.051948	0.183668	1.821947	1.20E-05	2.58E-05
AC145124.1	0.458393	0.191961	-1.25577	1.39E-17	1.63E-16
AC132192.2	0.271942	0.862596	1.665386	2.71E-15	2.29E-14
FP671120.4	0.320403	0.760495	1.247048	0.001056	0.001698
AC011899.1	0.084588	0.040483	-1.06316	1.96E-09	7.10E-09
AC104024.2	0.028084	0.291046	3.373441	2.65E-18	3.42E-17
Z69720.1	0.019735	0.05223	1.404112	0.000625	0.001039
AC107884.2	0.144727	0.330346	1.190648	0.000531	0.000892
AC099343.2	0.246351	0.699036	1.504653	5.25E-08	1.58E-07
AL162632.3	0.014409	0.059422	2.043973	6.63E-12	3.45E-11
AL160191.1	0.057587	0.016951	-1.7644	4.19E-12	2.25E-11
LINC01863	1.96973	0.224119	-3.13566	6.79E-31	1.17E-28
AC104257.1	0.351281	0.03523	-3.31775	1.15E-32	3.58E-30
AC006557.1	0.037577	0.089263	1.248232	0.000128	0.000235
AL355974.2	0.752078	0.112779	-2.73738	1.53E-25	6.27E-24
AC009237.15	1.159336	2.42488	1.064615	0.000627	0.001042
ACTN1-AS1	0.040374	0.092974	1.203404	1.87E-05	3.87E-05
LINC01374	0.069514	0.142208	1.032617	0.028306	0.036337
AL139089.1	0.282736	0.738627	1.38539	6.39E-12	3.33E-11

AC083837.1	1.001179	0.362689	-1.4649	3.69E-08	1.14E-07
LINC01705	0.038431	0.665227	4.113519	9.72E-21	1.66E-19
LINC00567	0.008458	0.143938	4.088988	7.06E-06	1.56E-05
Z95331.1	0.022361	0.106072	2.245971	1.39E-10	5.96E-10
ZKSCAN2-DT	0.275935	0.862503	1.644204	3.35E-14	2.40E-13
BX284668.6	0.033407	0.16068	2.265984	1.89E-08	6.08E-08
AL023803.1	0.07474	0.270448	1.855395	1.28E-06	3.14E-06
AC009754.1	0.077867	0.537652	2.787594	0.00372	0.005481
LINC01124	0.513452	1.131296	1.139674	0.000443	0.000752
AP003472.2	0.010457	0.07524	2.84701	3.60E-09	1.26E-08
AC103809.1	0.112164	0.041116	-1.44783	2.54E-08	8.00E-08
SMCR5	0.013061	0.098075	2.908625	0.008129	0.011352
AL135902.1	0.207633	0.043769	-2.24604	1.11E-16	1.14E-15
AL591806.1	0.060787	0.191687	1.656926	1.00E-07	2.91E-07
AC064805.1	0.113711	0.034865	-1.70553	1.71E-19	2.57E-18
AC139491.2	0.038014	0.168694	2.149803	0.002008	0.003089
AL691482.3	0.866787	3.665672	2.080328	2.82E-12	1.54E-11
AL355312.3	1.352201	9.167241	2.761178	4.99E-18	6.22E-17
AP001160.1	0.326672	0.770837	1.238581	1.58E-13	1.01E-12
AL139123.1	0.142501	0.416097	1.545953	2.30E-10	9.53E-10
AC090970.2	0.140965	0.3148	1.159093	3.73E-06	8.54E-06
NKAIN3-IT1	0.004237	0.108453	4.67789	7.87E-08	2.33E-07
AL135960.1	0.274147	0.054077	-2.34187	6.60E-25	2.28E-23
AC002128.1	0.381885	0.781136	1.032434	1.21E-05	2.58E-05
AL356056.2	0.51664	0.164731	-1.64905	2.31E-21	4.35E-20
AC010754.1	0.990341	0.352957	-1.48843	5.42E-18	6.74E-17
AC091182.2	0.040002	0.189251	2.242158	0.011186	0.015274
AC129926.1	0.094998	0.523177	2.461336	0.000621	0.001034
AC130371.2	2.587134	0.9879	-1.38892	3.70E-22	7.74E-21
DGCR9	0.092894	0.857617	3.206684	1.67E-18	2.22E-17
AC005618.1	0.146092	0.385901	1.401357	1.02E-06	2.55E-06
AL161452.1	0.1034	0.265647	1.361275	1.98E-11	9.68E-11
AP000821.1	0.071334	0.021058	-1.7602	5.05E-11	2.33E-10
AL035420.1	0.02467	0.07462	1.596829	0.000672	0.001109
AC093904.4	0.023883	0.356678	3.900592	1.20E-15	1.05E-14
AC093772.1	0.354242	0.12369	-1.518	1.04E-19	1.62E-18
AL161781.2	0.036638	0.083739	1.192554	1.20E-06	2.97E-06
AC007861.1	0.057185	0.009067	-2.65691	4.89E-22	9.93E-21
AL033527.3	0.042178	0.183975	2.124949	7.31E-15	5.75E-14
AC063919.1	0.282658	0.122753	-1.2033	4.41E-10	1.75E-09
AC009743.1	0.009597	0.16611	4.11346	2.40E-06	5.65E-06
LINC01779	0.055276	0.013864	-1.99527	9.39E-13	5.47E-12
AL671710.1	0.156119	0.315398	1.014528	1.66E-13	1.06E-12
AC044802.2	0.051752	0.172422	1.736259	3.06E-06	7.09E-06

AL049875.1	0.045051	0.18367	2.027488	2.34E-06	5.52E-06
AC103591.3	0.315	1.069126	1.763009	2.39E-05	4.87E-05
AL136162.1	0.109154	0.462805	2.084043	1.06E-19	1.64E-18
AC093734.1	0.040648	0.108355	1.4145	0.005615	0.008034
MELTF-AS1	0.570993	1.849425	1.695531	1.03E-16	1.06E-15
PTPRD-AS1	0.544879	0.125584	-2.11728	3.80E-27	2.11E-25
AC079336.2	0.079381	0.204145	1.362733	4.99E-07	1.30E-06
AC090192.2	0.006301	0.24065	5.255235	1.95E-10	8.13E-10
LUCAT1	0.196535	2.14012	3.444832	3.18E-15	2.65E-14
AC010973.1	0.201437	0.405231	1.008414	9.50E-06	2.06E-05
LINC00628	0.005807	0.100311	4.110584	6.74E-19	9.51E-18
MIR3150BHG	0.015081	0.088902	2.559521	1.11E-11	5.63E-11
LINC02223	0.004347	0.080932	4.218775	1.90E-07	5.26E-07
AC007879.3	0.08492	0.266559	1.650285	1.99E-15	1.70E-14
AC107214.1	0.189309	0.398955	1.075485	3.63E-10	1.46E-09
AL132639.3	0.153553	0.311303	1.019581	5.55E-07	1.44E-06
AC004771.1	0.353919	0.719779	1.024136	4.66E-08	1.42E-07
LINC02323	0.050542	0.42819	3.082701	4.96E-14	3.43E-13
AL683807.1	0.259302	0.723233	1.479829	2.28E-06	5.39E-06
AL158825.2	0.169965	0.372629	1.132499	0.000102	0.00019
AL158168.1	0.143902	0.039516	-1.86457	3.60E-05	7.14E-05
AL157788.1	0.053118	0.206443	1.958471	3.82E-07	1.01E-06
AC007368.1	0.015361	0.391904	4.673125	3.60E-06	8.25E-06
AL356740.2	0.062263	0.224042	1.847332	0.001829	0.002836
ATP2A1-AS1	0.304132	1.028492	1.757763	1.17E-15	1.02E-14
AC092746.1	0.09656	0.040588	-1.25036	1.46E-09	5.40E-09
LINC01907	0.019521	0.045882	1.232926	4.99E-05	9.74E-05
CASK-AS1	0.045116	0.128564	1.510766	5.03E-07	1.31E-06
AC023794.5	0.026271	0.096536	1.877582	2.29E-07	6.28E-07
LINC02471	5.253083	1.267949	-2.05067	5.73E-24	1.67E-22
AC093865.1	0.036281	0.203424	2.487223	6.57E-05	0.000126
AL122008.3	0.014483	0.077007	2.410631	9.42E-08	2.75E-07
LINC01136	0.03065	0.123036	2.005119	2.37E-13	1.48E-12
UMODL1-AS1	0.915254	0.251538	-1.86339	1.23E-07	3.50E-07
ASMTL-AS1	1.285859	3.098389	1.268786	3.00E-05	6.00E-05
AL158055.1	0.156964	0.066477	-1.2395	6.90E-17	7.29E-16
AC109779.1	0.077344	0.032944	-1.23128	5.45E-12	2.87E-11
AC002401.1	0.020558	0.084681	2.042346	5.40E-07	1.40E-06
AC015712.1	0.138727	0.613406	2.144589	7.88E-14	5.25E-13
AL161937.2	0.072369	0.261561	1.853715	3.10E-13	1.90E-12
VAC14-AS1	0.019574	0.141122	2.849964	1.16E-17	1.38E-16
AC133528.1	0.28108	0.56647	1.011017	0.00025	0.000438
AC026765.2	0.036155	0.010376	-1.80091	9.94E-12	5.07E-11
LINC01206	0.000997	0.017113	4.101777	3.38E-06	7.78E-06

AC145343.1	0.221444	2.065506	3.22148	5.19E-29	4.89E-27
AC010186.3	0.966851	2.492182	1.366044	4.16E-11	1.94E-10
AC114489.1	0.013609	0.311029	4.514398	1.70E-10	7.19E-10
AL360091.1	0.164279	0.358539	1.125979	0.002309	0.003512
AC073115.2	0.030242	0.140734	2.218325	3.11E-05	6.20E-05
LY6E-DT	0.221208	0.497126	1.16821	0.005548	0.007951
AC067945.3	0.191078	0.415948	1.122239	3.04E-05	6.09E-05
LINC01016	0.024092	0.010992	-1.13214	1.84E-10	7.72E-10
AC011373.1	0.050429	0.217517	2.108804	0.004074	0.005967
LINC02346	0.022959	0.003517	-2.70659	1.00E-19	1.57E-18
AC122710.2	0.036665	0.178752	2.285496	6.88E-14	4.65E-13
AC245014.1	0.058099	0.116347	1.001864	0.003562	0.005269
AC003986.2	0.002942	0.030867	3.39137	9.51E-08	2.77E-07
AC007773.1	0.277986	0.894943	1.686783	1.49E-17	1.74E-16
AC011330.2	0.160342	0.353079	1.138839	1.18E-07	3.39E-07
AL353611.1	0.051065	0.008595	-2.57077	8.14E-16	7.28E-15
FBXL19-AS1	0.479569	1.111157	1.212253	1.19E-10	5.16E-10
AL355974.1	0.045175	0.007445	-2.6011	2.28E-12	1.26E-11
AC090559.1	4.404392	1.617631	-1.44506	2.12E-23	5.45E-22
AC005363.2	0.05032	0.128987	1.358015	0.000327	0.000565
AC116914.2	0.741293	1.498221	1.015134	4.98E-09	1.71E-08
AC008147.2	0.047485	0.144032	1.600851	2.94E-05	5.89E-05
LINC00656	0.393582	0.038	-3.37259	1.81E-30	2.64E-28
AC147067.2	1.822763	0.477954	-1.93118	1.80E-24	5.75E-23
STAG3L5P-PVRIG2P-PILRB	0.339149	0.694518	1.034092	1.35E-08	4.41E-08
AL590666.3	0.001769	0.021446	3.599578	2.06E-13	1.30E-12
AC026391.1	0.096933	0.014824	-2.70906	6.70E-24	1.87E-22
AP001046.1	0.007104	0.023919	1.751415	3.53E-06	8.10E-06
AC099509.1	0.118578	1.860224	3.971573	1.10E-15	9.61E-15
KTN1-AS1	0.275314	0.589441	1.098271	6.69E-14	4.52E-13
AC016245.1	0.004934	0.028502	2.530361	0.001927	0.002974
AL596223.1	0.007042	0.107013	3.925663	6.83E-09	2.32E-08
AC010999.2	0.07147	0.155898	1.125185	0.000643	0.001066
LINC02016	3.561789	0.127354	-4.80569	1.38E-33	6.11E-31
ZNF337-AS1	0.220618	0.486592	1.141158	5.20E-12	2.75E-11
LINC00706	0.018493	0.111304	2.589487	5.76E-05	0.000111
LINC00968	2.726315	0.257438	-3.40466	3.20E-32	8.65E-30
AC244093.5	0.09621	0.264955	1.461492	3.08E-06	7.12E-06
AC090833.1	0.004924	0.062766	3.672005	7.01E-08	2.08E-07
HOXC-AS1	0.041661	0.4648	3.479842	1.95E-07	5.38E-07
AC008875.1	0.134059	0.613975	2.195313	2.66E-13	1.65E-12
LINC00574	0.037722	0.087609	1.215676	0.00017	0.000305
ABCA9-AS1	0.00768	0.097284	3.663047	0.005941	0.008469

AC104237.3	1.161525	0.109297	-3.4097	8.85E-34	4.23E-31
AC010149.1	0.105713	0.341754	1.692805	2.07E-08	6.61E-08
AP006545.1	0.052635	0.152292	1.532751	7.38E-06	1.62E-05
AP000851.1	0.025727	0.124501	2.274801	0.001257	0.001998
LINC00472	0.769774	0.270069	-1.51111	1.17E-26	5.82E-25
AC105046.1	0.149978	0.032818	-2.19217	1.12E-07	3.23E-07
AC090739.1	0.39116	1.1974	1.614074	0.000724	0.001189
AP000873.2	0.306497	0.748152	1.287461	2.20E-12	1.22E-11
AC015726.1	2.451545	1.092461	-1.16611	9.24E-19	1.28E-17
AC055713.1	0.151563	0.481732	1.668317	2.89E-20	4.70E-19
AP000864.1	0.021492	0.070733	1.718554	0.000279	0.000486
AC074212.1	0.255897	0.514324	1.007115	9.53E-06	2.06E-05
AC015923.1	0.024466	0.353643	3.85346	3.84E-05	7.59E-05
AC011893.1	0.013978	0.049923	1.836588	3.00E-10	1.22E-09
AP001596.2	0.089254	0.030409	-1.55343	5.61E-08	1.69E-07
LINC02086	0.018452	0.302892	4.036969	2.16E-10	8.98E-10
AL353593.3	0.114384	0.367698	1.68464	0.015117	0.020199
HLX-AS1	0.411783	0.08126	-2.34126	3.69E-24	1.13E-22
FAM198B-AS1	0.21566	0.457517	1.085065	0.026428	0.034038
USP3-AS1	0.221785	0.476908	1.104547	1.09E-07	3.14E-07
AC063965.1	0.197937	1.106291	2.482617	0.00673	0.009518
AL365181.2	0.039389	1.620899	5.362847	5.48E-17	5.89E-16
AC107081.2	0.207473	0.485756	1.227306	1.54E-08	4.99E-08
AC123023.1	0.537165	0.126212	-2.08951	2.23E-14	1.63E-13
AC093110.1	10.04202	1.1877	-3.07981	6.99E-31	1.17E-28
AC011444.3	0.324713	0.100381	-1.69369	6.74E-18	8.29E-17
LINC01535	0.115415	0.549705	2.251833	1.11E-08	3.68E-08
AL607028.1	0.034391	0.102031	1.568912	0.005046	0.007279
SOX21-AS1	0.150239	0.751365	2.322251	0.006075	0.008651
MAGEA10-MAGEA5	0.004797	0.067363	3.811617	1.42E-07	4.02E-07
AC021016.2	2.681199	0.960927	-1.48038	7.38E-30	8.19E-28
USP30-AS1	4.131418	1.873172	-1.14115	4.77E-17	5.18E-16
AC087742.1	0.082572	0.197999	1.261765	1.46E-05	3.08E-05
AC134312.1	0.139438	0.026751	-2.38194	2.37E-21	4.45E-20
AC104791.1	0.023182	0.098229	2.083146	0.002982	0.004464
AC003070.2	0.399846	0.115694	-1.78913	4.83E-22	9.85E-21
AP000487.1	0.157733	0.350186	1.150632	6.33E-15	5.02E-14
AL122019.1	0.004422	0.128809	4.864378	4.11E-09	1.43E-08
AC006483.2	0.030634	0.07599	1.310661	0.008073	0.011283
GS1-24F4.2	0.012327	0.061947	2.329175	0.000227	0.000402
AC022150.4	0.423199	0.938663	1.149271	0.00293	0.004393
AC005726.3	0.740426	1.731723	1.22578	2.16E-09	7.73E-09
AL109910.2	0.020469	0.008123	-1.33325	6.34E-12	3.31E-11
PGM5-AS1	0.525044	0.117342	-2.16172	3.03E-25	1.15E-23

LINC01397	0.018518	0.09673	2.38502	3.20E-07	8.59E-07
LINC02407	0.108105	0.328537	1.603629	1.47E-09	5.41E-09
AC068987.4	0.451786	1.170624	1.373567	0.019919	0.026203
AC116021.1	0.03305	0.118636	1.843833	1.60E-05	3.34E-05
AC009630.2	0.00334	0.02637	2.98083	5.87E-08	1.76E-07
AC084125.4	0.151708	0.449985	1.568576	9.00E-11	3.97E-10
AL365356.1	0.024037	0.323663	3.751167	0.003327	0.004943
AL365356.5	0.016779	0.851753	5.665724	1.01E-24	3.40E-23
AL354892.2	1.607149	3.36425	1.065781	3.46E-13	2.11E-12
AC006213.3	0.108174	0.415918	1.94295	4.86E-07	1.28E-06
AC004466.3	0.167897	0.529302	1.656512	1.08E-06	2.70E-06
AL596218.1	0.063444	0.629407	3.310437	0.000186	0.000332
FAM181A-AS1	0.317286	0.084784	-1.90393	2.48E-17	2.77E-16
AL445524.1	1.114097	7.902802	2.82649	7.04E-29	6.53E-27
AC011445.2	0.322213	0.921374	1.51577	2.18E-08	6.95E-08
AC068722.1	0.002319	0.039861	4.103324	8.18E-09	2.74E-08
AC084262.1	0.010442	0.222609	4.41404	6.21E-14	4.23E-13
NHEG1	0.039965	0.009131	-2.12998	1.27E-11	6.38E-11
AC120193.1	0.022149	0.132063	2.575925	4.06E-07	1.07E-06
AL161618.1	1.138955	0.237956	-2.25895	1.53E-12	8.70E-12
HOXB-AS4	0.011591	0.279101	4.589674	1.40E-07	3.95E-07
AC097658.2	0.040754	0.010649	-1.93626	3.74E-12	2.02E-11
AC104241.2	0.089185	0.028233	-1.65944	2.83E-16	2.71E-15
AC004884.2	0.097195	0.968177	3.316313	2.05E-12	1.14E-11
AC011447.3	0.105527	0.395348	1.905511	0.008645	0.012019
LINC01629	0.033712	0.43229	3.680661	9.74E-07	2.44E-06
BLACAT1	0.223393	1.873126	3.06779	4.41E-22	9.08E-21
AL359694.2	0.036777	0.097586	1.407866	0.019192	0.025263
AC011503.2	0.240332	1.117157	2.216731	3.58E-19	5.26E-18
GAS1RR	0.187003	0.076448	-1.2905	1.28E-19	1.95E-18
AC090921.1	0.027783	0.085193	1.616515	0.000212	0.000377
C5orf64	0.051578	0.014146	-1.86642	2.05E-23	5.31E-22
AC079684.1	0.239144	0.806481	1.753761	6.17E-12	3.23E-11
AL591178.1	1.164289	0.48987	-1.24898	2.49E-10	1.03E-09
AC067930.1	0.035955	0.09102	1.339992	0.001405	0.002215
AC245884.11	0.110401	0.042295	-1.38421	1.34E-14	1.00E-13
AL034399.2	0.003171	0.102269	5.011512	1.56E-15	1.35E-14
MGAT3-AS1	0.609082	0.107599	-2.50098	5.69E-27	3.02E-25
AF131215.5	2.481672	0.871447	-1.50983	1.18E-22	2.68E-21
SIRPG-AS1	0.015543	0.075806	2.286086	5.62E-07	1.45E-06
AC006329.1	0.648086	3.087104	2.251996	6.50E-14	4.40E-13
PRR7-AS1	0.125166	0.317012	1.340691	1.09E-06	2.70E-06
ARHGEF7-AS2	0.020006	0.009875	-1.0186	3.05E-06	7.08E-06
AL354714.1	0.969028	0.067237	-3.8492	2.91E-32	8.22E-30

L29074.1	0.014493	0.066276	2.193177	1.34E-05	2.84E-05
AC026401.2	0.048471	0.165117	1.768301	0.00147	0.002306
AL022324.3	0.003579	0.090218	4.655906	1.88E-11	9.24E-11
AC006449.3	0.040233	0.156874	1.963171	5.09E-10	2.00E-09
AL139099.2	0.167613	0.402517	1.263915	1.21E-08	3.98E-08
AC004540.2	2.313609	0.646502	-1.83942	2.32E-25	9.13E-24
AL390037.1	0.041376	0.085448	1.046247	0.003926	0.005765
AL132780.1	0.281996	0.579554	1.03927	2.53E-09	9.00E-09
ANKRD10-IT1	5.091996	10.36725	1.02573	1.60E-06	3.88E-06
LINC00173	0.123922	0.369831	1.577438	9.90E-08	2.87E-07
AC005993.1	0.029602	0.990397	5.064257	1.58E-06	3.85E-06
AL356108.1	0.019459	0.079893	2.037595	0.000324	0.00056
ALG1L9P	0.158529	0.351838	1.150161	2.88E-15	2.41E-14
AL513320.1	0.214248	0.630724	1.557725	8.06E-11	3.58E-10
AC107959.1	0.827923	0.400357	-1.04821	1.82E-22	3.99E-21
AL359513.1	0.255402	0.667264	1.385487	8.67E-12	4.45E-11
LINC00513	0.189588	1.142262	2.590951	0.000199	0.000355
AL133370.1	0.026134	1.753739	6.068365	9.21E-08	2.69E-07
LINC01269	0.179791	0.864434	2.265437	9.85E-12	5.03E-11
AL139339.1	0.294135	0.146072	-1.0098	5.23E-13	3.13E-12
AC004672.1	0.006847	0.06425	3.230123	0.010895	0.01489
AC104785.1	0.170783	0.346109	1.01906	8.95E-05	0.000168
AC104984.1	0.734334	0.044191	-4.05461	1.15E-29	1.21E-27
AP000569.1	0.052132	0.151459	1.538672	1.36E-07	3.86E-07
AL590723.1	0.262469	0.732295	1.480278	1.69E-06	4.09E-06
LINC01597	0.040827	0.637357	3.964523	7.35E-10	2.84E-09
AC083973.1	0.012219	0.092213	2.915868	7.88E-12	4.08E-11
AP003170.3	0.342567	0.757673	1.145187	0.001539	0.002407
LINC01166	0.082771	0.017926	-2.20707	3.47E-21	6.42E-20
AL355388.2	0.167731	0.487206	1.538378	7.56E-15	5.92E-14
LINC02475	0.012784	0.257439	4.331861	9.58E-11	4.22E-10
AL590004.3	0.047614	0.331748	2.800639	4.44E-08	1.36E-07
LNX1-AS2	0.022659	0.123538	2.446818	2.10E-11	1.03E-10
AQP4-AS1	0.162674	0.074249	-1.13154	2.98E-10	1.21E-09
STEAP2-AS1	0.008246	0.112442	3.769342	4.79E-14	3.32E-13
CASC16	0.006397	0.160449	4.648517	5.83E-15	4.64E-14
AL365259.1	0.052771	0.021891	-1.26944	8.24E-13	4.82E-12
DENND5B-AS1	0.027423	0.09431	1.782055	1.43E-05	3.02E-05
AC126323.6	0.008122	0.095329	3.552979	1.69E-07	4.72E-07
AC048383.1	0.013588	0.108068	2.991573	8.64E-06	1.88E-05
AC072062.1	0.004987	0.045491	3.189223	6.27E-10	2.44E-09
PGM5P3-AS1	0.176847	0.064762	-1.44928	1.24E-19	1.88E-18
AC008115.3	0.535431	1.547125	1.530817	1.38E-13	8.87E-13
AC016590.2	0.214896	0.66624	1.632405	0.001071	0.001721

AL022322.1	0.266689	1.091306	2.032826	2.73E-12	1.50E-11
AL158063.1	0.118833	0.243563	1.03536	0.000178	0.000319
AC087241.3	0.016961	0.037288	1.13653	0.021258	0.027823
AC010320.3	0.140606	0.312823	1.153693	8.01E-05	0.000152
MIR31HG	0.053372	0.583939	3.451654	2.44E-05	4.96E-05
AL139420.1	0.005385	0.170349	4.983338	3.50E-15	2.89E-14
AC009121.1	0.032824	0.198142	2.593717	3.42E-17	3.78E-16
LINC00853	0.475149	1.207062	1.345048	2.77E-10	1.13E-09
LINC00637	0.022459	0.063619	1.502131	8.39E-07	2.12E-06
AC044810.2	0.414604	0.048014	-3.1102	2.03E-30	2.81E-28
AC008040.1	0.006494	0.017506	1.430597	1.16E-05	2.48E-05
MAP3K14-AS1	0.576634	1.3313	1.207107	3.77E-18	4.78E-17
AC093248.1	0.031406	0.088425	1.493438	0.019489	0.025648
AL136084.2	0.018014	0.058432	1.697685	2.02E-10	8.42E-10
AC024560.3	0.264449	0.551473	1.060302	1.55E-05	3.25E-05
AL591846.2	0.151394	0.370107	1.289634	5.52E-08	1.66E-07
AL121772.1	0.122552	0.531053	2.115466	4.57E-13	2.75E-12
AC092546.1	0.015799	0.047607	1.59135	0.000673	0.001111
AC245884.1	0.016766	0.049765	1.569617	0.000866	0.001411
AC089983.1	0.039103	0.9418	4.590069	1.54E-14	1.14E-13
AC079089.1	0.018366	0.205816	3.486241	1.90E-20	3.15E-19
C22orf34	0.612608	0.297464	-1.04225	1.30E-18	1.75E-17
AC096921.2	1.887184	0.607197	-1.636	4.92E-27	2.66E-25
AC244100.3	0.047413	0.019283	-1.29797	0.000559	0.000938
AL359880.1	0.043963	0.253865	2.529695	0.014358	0.019259
PACRG-AS3	0.142184	0.016765	-3.08421	3.36E-30	4.32E-28
AC040174.1	0.002264	0.061356	4.760362	4.01E-07	1.06E-06
AC113146.1	0.009599	0.10809	3.493222	0.00271	0.004082
SPRY4-AS1	0.248242	0.522587	1.073926	1.47E-05	3.10E-05
AC253536.6	0.28747	0.638146	1.150474	4.17E-09	1.44E-08
POT1-AS1	0.079103	0.236701	1.581265	6.66E-09	2.26E-08
AL589765.5	0.175071	0.354985	1.019819	1.06E-06	2.65E-06
STAU2-AS1	0.062621	0.305864	2.288164	1.76E-15	1.51E-14
AC128707.1	0.046099	0.007628	-2.59532	6.17E-15	4.90E-14
AC008403.3	0.045817	0.164271	1.842113	8.98E-08	2.62E-07
AC083806.2	0.030175	0.147052	2.28492	8.39E-06	1.83E-05
TARID	0.391447	0.167372	-1.22576	4.36E-21	7.93E-20
SPANXA2-OT1	0.014375	0.053185	1.887406	0.000386	0.00066
LINC01977	0.030719	0.994141	5.016249	4.20E-30	4.92E-28
AC012157.1	0.076589	0.176434	1.203916	0.002773	0.00417
AC034238.1	0.015257	0.040744	1.417108	0.001344	0.002125
AC005165.1	0.522807	0.25823	-1.01762	1.01E-14	7.66E-14
SH3RF3-AS1	0.881971	0.397112	-1.15119	6.49E-16	5.87E-15
AC022075.1	0.258923	0.76906	1.570571	8.35E-07	2.11E-06

AC100774.1	0.012619	0.160275	3.666829	0.000129	0.000237
AP001062.2	0.061345	0.213631	1.800108	9.96E-05	0.000186
AC024361.1	0.365441	1.042916	1.512912	1.55E-10	6.61E-10
AC078923.1	0.020664	0.38402	4.21596	3.19E-15	2.65E-14
LINC01449	0.006263	0.035254	2.492962	4.36E-08	1.34E-07
AL161740.1	0.271085	0.030951	-3.13068	6.08E-23	1.45E-21
AC021127.1	0.019076	0.106719	2.484011	7.18E-06	1.58E-05
AP001646.3	0.120564	0.049896	-1.27279	8.53E-08	2.50E-07
LINC02245	0.007465	0.029111	1.963298	3.34E-08	1.04E-07
AP002754.1	0.031736	0.199565	2.652665	2.70E-06	6.32E-06
AC026369.1	0.085754	0.026509	-1.69372	7.97E-22	1.57E-20
LINC02397	0.020442	0.055291	1.435552	1.57E-07	4.41E-07
LINC01927	0.232966	0.068459	-1.76679	2.37E-08	7.51E-08
AC093334.1	0.04577	0.022734	-1.00952	1.57E-07	4.40E-07
AC011352.1	0.002232	0.07916	5.148584	2.05E-11	1.00E-10
ZNF571-AS1	0.09104	0.359625	1.981918	1.44E-05	3.04E-05
AC078906.1	0.014955	0.073998	2.30685	0.000233	0.000412
AP005230.1	0.014058	0.237937	4.081149	5.89E-14	4.02E-13
VIPR1-AS1	0.517456	0.237303	-1.1247	2.69E-09	9.53E-09
AC025043.1	0.103997	0.24018	1.207583	0.010904	0.014898
LINC01290	1.941611	0.476029	-2.02813	3.55E-25	1.31E-23
AC010148.1	0.03505	0.132723	1.920916	7.33E-09	2.48E-08
LEMD1-AS1	0.017265	0.105807	2.615492	6.49E-09	2.21E-08
AP003973.2	0.227956	0.098005	-1.21783	9.68E-11	4.27E-10
AC027288.3	5.220423	0.620427	-3.07283	3.40E-30	4.32E-28
AL121904.1	0.017812	0.284024	3.995102	7.50E-05	0.000142
AC027801.4	0.079649	0.263667	1.726986	4.76E-07	1.25E-06
AC024581.1	0.00688	0.067201	3.287942	3.06E-07	8.22E-07
FUT8-AS1	0.208004	0.628031	1.594224	5.60E-16	5.09E-15
LINC02594	0.014503	0.17154	3.564103	2.64E-09	9.37E-09
AC016987.2	0.140275	0.028575	-2.29541	8.58E-17	8.95E-16
AC048382.2	0.355159	0.781257	1.137332	5.06E-06	1.14E-05
LINC00427	0.050523	0.173922	1.783427	0.007671	0.010756
SMC2-AS1	0.113519	0.053325	-1.09005	6.25E-12	3.27E-11
AC007546.1	0.303273	0.631856	1.05898	0.000946	0.00153
AL109614.1	0.311598	0.706297	1.180588	0.003897	0.005726
AL160291.1	0.01426	0.046326	1.699824	2.98E-07	8.04E-07
AC010776.3	0.481655	0.036127	-3.73685	4.54E-40	2.82E-36
TMEM78	0.001653	0.014938	3.175538	1.87E-09	6.78E-09
AC090772.1	0.075399	0.349867	2.214189	1.05E-09	3.95E-09
AC108174.1	0.009752	0.069243	2.827858	2.62E-05	5.30E-05
AL352984.1	0.016868	0.362205	4.424474	1.68E-15	1.45E-14
WASHC5-AS1	0.132204	0.409262	1.630261	7.90E-08	2.34E-07
AL162574.1	0.042997	0.01663	-1.37042	1.37E-08	4.47E-08

AC008667.1	0.020482	0.096667	2.238674	6.85E-05	0.000131
AC107032.2	0.226501	0.478987	1.08047	1.62E-07	4.54E-07
AC073333.1	0.147857	0.445157	1.590113	1.03E-07	2.99E-07
LINC00592	0.059524	0.255928	2.104199	2.23E-07	6.11E-07
AP001429.1	0.077535	0.81593	3.395526	1.03E-07	2.97E-07
AP002957.1	0.010034	0.223585	4.477791	7.95E-18	9.68E-17
AC025766.1	0.12733	0.51696	2.021483	4.78E-14	3.32E-13
AC026740.1	0.512995	1.457895	1.506872	2.09E-09	7.49E-09
LINC02387	0.027318	0.325293	3.573805	2.47E-09	8.77E-09
LINC01607	0.191782	1.186732	2.629456	1.86E-21	3.52E-20
LINC02213	0.033981	0.010907	-1.63939	2.82E-12	1.54E-11
AL136526.1	0.020436	0.048344	1.242236	0.019189	0.025263
LINC01827	0.611485	0.162826	-1.90899	2.54E-18	3.29E-17
AC087477.5	0.109784	0.04615	-1.25026	4.96E-12	2.63E-11
AC006504.1	0.112646	0.30728	1.447754	0.005034	0.007263
LINC01511	0.012033	0.643462	5.740814	2.99E-06	6.95E-06
AC105137.2	0.051702	0.215979	2.062589	3.63E-16	3.43E-15
AL034550.1	0.169342	0.387932	1.195865	1.31E-06	3.21E-06
AC004253.1	0.4523	1.046341	1.210001	8.94E-09	2.98E-08
FAM242C	0.018444	0.069325	1.910234	4.89E-07	1.28E-06
LINC02449	0.362794	0.739154	1.026724	0.000131	0.00024
CR559946.1	0.022821	0.072743	1.672457	1.70E-06	4.11E-06
FENDRR	9.354303	0.648816	-3.84975	7.85E-32	1.95E-29
LINC00866	0.047358	0.378316	2.997918	3.89E-15	3.18E-14
AL391845.2	0.017497	0.084863	2.278059	9.55E-16	8.42E-15
AC092436.4	0.064294	0.385217	2.582904	2.73E-05	5.51E-05
AC009093.1	0.071698	0.200866	1.48624	3.24E-07	8.68E-07
AC018647.1	0.553424	0.094286	-2.55326	1.41E-29	1.44E-27
AC090912.1	0.134036	0.37572	1.487034	2.99E-10	1.22E-09
AC118755.2	0.100032	0.204327	1.030411	0.0006	0.001002
AL355472.4	0.077836	0.308681	1.987603	1.74E-09	6.36E-09
AL731557.1	0.324325	0.091801	-1.82086	1.86E-22	4.06E-21
UTAT33	0.195325	0.497592	1.349084	2.46E-10	1.02E-09
AP001605.1	0.004479	0.014082	1.652502	0.00829	0.011553
AC025048.1	0.270559	0.047974	-2.49561	3.99E-23	9.81E-22
AP002989.1	0.02203	0.055571	1.334837	0.013979	0.018807
AC245041.2	4.209781	0.761215	-2.46737	3.93E-28	2.81E-26
AC010894.3	0.024605	0.255555	3.376615	0.003922	0.00576
AL158847.1	0.209818	0.051581	-2.02423	5.39E-24	1.58E-22
AC063944.1	0.10161	0.042816	-1.24684	6.50E-24	1.83E-22
AC006480.2	0.223275	0.468316	1.068663	8.69E-05	0.000164
AC027796.4	0.492855	1.287118	1.384909	2.63E-08	8.28E-08
AC025259.1	0.030051	0.011649	-1.36716	3.60E-07	9.61E-07
LINC02330	0.054396	0.006276	-3.11547	1.84E-27	1.10E-25

LINC01389	0.244021	0.780146	1.676738	3.37E-13	2.06E-12
KCNQ1OT1	0.047336	0.264635	2.482988	3.06E-06	7.09E-06
GSEC	0.49524	1.321187	1.415635	1.91E-16	1.88E-15
AC005332.5	1.504278	3.098266	1.042389	4.37E-06	9.92E-06
AC020658.4	0.006539	0.054154	3.049946	9.06E-10	3.44E-09
LINC01031	0.222853	0.070973	-1.65075	1.46E-18	1.95E-17
AL034417.2	0.098576	0.35203	1.836391	5.55E-14	3.81E-13
LINC01833	0.004265	0.451799	6.726961	2.37E-16	2.29E-15
AC006252.1	0.144243	0.345408	1.259805	4.77E-14	3.31E-13
AC245014.3	0.181356	0.586575	1.693489	0.00561	0.008029
AC048341.2	0.688969	2.548901	1.887365	1.34E-14	1.00E-13
ADAMTS9-AS1	0.71696	0.194687	-1.88074	7.06E-28	4.67E-26
AL354993.1	0.018442	0.108637	2.558418	0.002114	0.003236
AL118511.1	0.29756	0.640561	1.106153	1.80E-09	6.55E-09
AC009097.2	0.039072	0.190084	2.282444	1.39E-10	5.98E-10
AL645608.8	0.029804	0.579867	4.282147	1.56E-22	3.46E-21
AL080317.1	1.277974	2.834727	1.149351	1.63E-12	9.24E-12
LINC01985	0.43136	0.075095	-2.5221	8.35E-28	5.41E-26
AL596442.2	0.81158	0.393914	-1.04285	3.86E-15	3.16E-14
AC243547.1	0.033679	0.131247	1.962384	8.86E-08	2.59E-07
LINC00479	0.023979	0.066971	1.481734	0.002899	0.004347
LINC00707	0.021755	0.513633	4.561287	1.45E-09	5.34E-09
AL596223.2	0.052005	0.194415	1.902424	9.70E-07	2.43E-06
AC092681.3	0.015924	0.062895	1.981736	0.003596	0.005314
AC137767.1	0.165494	0.344156	1.056282	2.47E-07	6.72E-07
AC008894.1	0.077642	0.023295	-1.73684	1.42E-10	6.07E-10
AC138625.1	0.042863	0.122278	1.512357	0.006472	0.009191
FOXD2-AS1	0.551077	1.909435	1.79282	1.08E-25	4.52E-24
AL096828.3	0.237988	1.08768	2.19229	1.20E-23	3.21E-22
AC005697.2	0.009455	0.01898	1.005348	0.016133	0.021459
SLC5A4-AS1	0.07752	0.181064	1.223863	0.005251	0.007563
AC025580.3	0.009266	0.021544	1.217243	0.018292	0.024134
AC079906.1	0.032388	0.080764	1.318246	9.62E-06	2.08E-05
KIZ-AS1	0.014825	0.067882	2.195041	4.77E-06	1.08E-05
AL445483.1	0.15168	0.394996	1.380807	0.000163	0.000293
AC007953.1	0.076082	0.293001	1.945281	0.000368	0.000632
AL445183.2	0.034583	0.419854	3.601739	9.96E-06	2.15E-05
AL161645.1	0.023757	0.100429	2.079735	0.000164	0.000295
AL354719.2	0.121193	2.097003	4.112949	4.07E-22	8.47E-21
AC023906.5	0.165999	0.379188	1.191739	8.32E-05	0.000157
BX005040.1	0.200806	0.041073	-2.28956	1.04E-12	6.02E-12
U62317.3	0.08833	0.202797	1.199067	8.93E-07	2.25E-06
DKFZp779M0652	0.631019	0.310569	-1.02277	7.23E-16	6.50E-15
AC090204.1	2.337052	4.798072	1.037765	0.000119	0.000219

NADK2-AS1	0.201127	0.521532	1.374648	0.020489	0.026902
AP000866.6	0.31304	0.721404	1.20446	0.024079	0.031232
AL354710.2	0.025917	0.065256	1.332214	0.005681	0.008125
AL031651.2	0.113594	0.249597	1.135711	0.000124	0.000228
AC002306.1	0.020756	0.084254	2.021251	0.004193	0.006124
AC082651.3	0.163989	0.070478	-1.21836	4.35E-12	2.33E-11
AC104699.1	0.282073	1.474172	2.385764	9.74E-13	5.66E-12
AC023157.3	0.941089	2.371772	1.333562	5.65E-15	4.51E-14
LINC01484	0.093489	0.377457	2.013445	1.06E-09	3.98E-09
TMEM147-AS1	0.73715	1.65541	1.167159	3.03E-12	1.65E-11
AC243654.3	0.039593	0.097457	1.299503	5.60E-05	0.000108
AP001432.1	0.313828	0.653474	1.058155	5.09E-09	1.75E-08
AL133243.2	0.428754	1.31283	1.614457	5.51E-13	3.27E-12
NARF-AS1	0.057653	0.236391	2.035712	3.15E-13	1.93E-12
TTLL10-AS1	0.197508	0.098223	-1.00779	5.19E-07	1.35E-06
SEMA6A-AS2	0.151779	0.035048	-2.11458	1.04E-17	1.25E-16
AC073957.3	0.347906	1.115603	1.681055	5.49E-11	2.51E-10
AC019118.1	0.026124	0.090976	1.800085	0.001087	0.001746
AC016999.1	0.069726	0.186357	1.418307	0.000153	0.000277
TMPO-AS1	0.24474	0.810284	1.727176	8.55E-24	2.35E-22
AC006947.1	0.044069	0.126248	1.518433	0.015409	0.020554
AC016065.1	0.666184	1.401075	1.072541	9.77E-18	1.17E-16
AL928654.2	1.410171	3.048322	1.112145	2.43E-07	6.65E-07
AC010336.1	0.009881	0.024068	1.284415	5.04E-06	1.13E-05
LINC01033	0.036246	0.015739	-1.20345	0.002532	0.003833
AC113349.1	1.228396	0.352799	-1.79986	2.03E-11	9.94E-11
LINC00346	0.42202	1.154662	1.452087	6.53E-06	1.44E-05
AC008750.3	0.026655	0.127011	2.252489	6.87E-07	1.75E-06
AC018410.2	0.195348	0.421715	1.110225	0.017848	0.023589
AC083880.1	0.211811	0.72367	1.772553	3.64E-16	3.43E-15
PTENP1-AS	0.075019	0.033372	-1.16862	1.21E-09	4.53E-09
AC006111.2	0.187004	0.515062	1.461672	4.47E-11	2.07E-10
LINC00665	0.63998	3.548814	2.471239	1.05E-13	6.88E-13
AL592071.1	0.083801	0.210942	1.331806	5.58E-07	1.44E-06
AC007182.1	0.183346	0.032263	-2.50664	7.91E-25	2.70E-23
AC021066.1	0.035195	0.154345	2.132716	4.38E-07	1.15E-06
Z94721.2	0.012911	0.072081	2.480987	2.93E-12	1.60E-11
AC108097.1	0.183564	0.083032	-1.14454	1.31E-05	2.79E-05
FGF10-AS1	0.079755	0.011112	-2.84345	2.85E-28	2.16E-26
LINC01978	0.028915	0.120042	2.053656	3.29E-11	1.56E-10
LINC01342	0.046401	0.129116	1.476439	0.000357	0.000614
AC135178.1	0.249945	0.077615	-1.6872	7.85E-15	6.10E-14
AP004608.1	0.119445	1.024555	3.100582	2.31E-06	5.47E-06
AC079467.1	5.61371	1.35321	-2.05257	1.56E-21	3.00E-20

AP005271.1	0.030324	0.160581	2.404764	1.19E-06	2.94E-06
BX255923.2	0.002788	0.009094	1.70577	5.37E-05	0.000104
LINC02560	0.408806	3.619529	3.146315	2.43E-13	1.51E-12
LINC01798	0.141563	0.066105	-1.09862	3.95E-16	3.69E-15
LINC02227	0.004534	0.019399	2.097213	2.91E-07	7.86E-07
LINC00639	0.31391	0.147727	-1.08742	1.13E-11	5.73E-11
AC091057.1	0.196863	0.592913	1.590626	1.31E-17	1.55E-16
AC114730.1	0.019622	0.045383	1.209654	0.001701	0.002647
AC116036.2	0.264409	0.675684	1.353577	1.78E-07	4.96E-07
RUNDC3A-AS1	0.022559	0.357183	3.984897	1.56E-28	1.31E-26
AL132657.1	0.256923	0.535305	1.059026	2.28E-06	5.39E-06
AL592494.3	0.166153	0.627944	1.918125	6.91E-10	2.68E-09
AC007639.1	0.012345	0.284233	4.525033	2.88E-20	4.70E-19
LINC01917	0.045677	0.01692	-1.43273	8.39E-10	3.20E-09
AC090844.3	0.159869	0.0372	-2.10352	9.32E-07	2.34E-06
Z94160.1	0.00578	0.03582	2.631513	4.39E-05	8.62E-05
AC078820.1	0.029677	0.328203	3.467159	1.30E-09	4.84E-09
AC090578.2	0.01142	0.175363	3.940659	2.68E-06	6.29E-06
AC009127.1	0.029038	0.075997	1.387998	0.005421	0.007788
CHL1-AS1	0.015152	0.123162	3.023	4.62E-06	1.05E-05
NCBP2-AS1	0.233637	0.519187	1.151986	5.11E-07	1.33E-06
AC008771.1	2.974224	6.052966	1.025129	4.28E-18	5.39E-17
AL159169.3	0.161631	0.489098	1.597415	0.005585	0.007996
AC010976.1	0.183694	0.383776	1.062961	0.000706	0.001162
AC139887.4	0.483593	1.131589	1.226484	0.022812	0.029694
KC877392.1	0.286773	0.059058	-2.2797	2.95E-17	3.28E-16
AC008764.6	0.224238	0.515652	1.201367	5.54E-13	3.29E-12
AC018653.3	0.601181	1.481195	1.300889	1.20E-10	5.21E-10
AC087878.1	0.036362	0.127773	1.813082	0.00243	0.003688
AC012645.3	0.268465	0.643391	1.26096	3.85E-08	1.19E-07
TAF1A-AS1	0.501014	1.142276	1.188988	5.00E-15	4.03E-14
MINCR	1.190726	2.520637	1.081947	6.13E-12	3.21E-11
AC092667.1	0.109489	0.044865	-1.28712	3.93E-20	6.36E-19
SEMA3B-AS1	2.695099	1.281502	-1.0725	1.84E-17	2.11E-16
AC004817.3	0.029772	0.128555	2.110356	0.000253	0.000444
AC091588.3	0.700025	0.196914	-1.82984	1.01E-20	1.73E-19
AC023590.1	0.086655	0.431357	2.315519	0.000157	0.000284
AC232271.1	0.398849	0.84641	1.085512	1.15E-08	3.80E-08
AC108451.2	0.047118	0.783067	4.054772	1.41E-05	2.97E-05
AC129507.4	0.274305	3.30013	3.588671	5.04E-05	9.82E-05
DLX6-AS1	0.003706	0.117821	4.990542	2.33E-10	9.63E-10
AC011773.4	0.027465	0.091508	1.736305	6.64E-07	1.70E-06
AC010307.2	0.039062	0.17946	2.199813	4.92E-06	1.11E-05
KIAA1614-AS1	0.024473	0.070317	1.522711	1.17E-06	2.90E-06

LINC02576	0.096501	0.532803	2.464989	5.77E-18	7.15E-17
FRMD6-AS1	0.079761	0.210351	1.399044	8.52E-07	2.15E-06
AC107959.2	0.011663	0.029691	1.348153	0.000641	0.001065
AP000695.1	0.223884	0.697765	1.639992	1.26E-10	5.47E-10
AL021026.1	0.180342	0.086621	-1.05795	1.21E-13	7.84E-13
HPAT5	0.035241	0.013669	-1.36637	2.35E-06	5.56E-06
AC017002.3	0.142479	0.343568	1.269845	9.53E-09	3.17E-08
AC004943.3	0.048414	0.14597	1.592185	4.40E-05	8.65E-05
AC020891.2	0.005164	0.191578	5.213379	3.93E-10	1.57E-09
CTD-2297D10.2	0.01485	0.074836	2.333227	3.00E-07	8.09E-07
GATA6-AS1	1.537469	0.494469	-1.63661	6.97E-23	1.65E-21
LINC00535	0.222913	0.098258	-1.18183	2.87E-19	4.22E-18
AP000266.1	0.038316	0.147716	1.946827	0.031759	0.040435
AC015712.6	0.040445	0.377781	3.22352	5.02E-12	2.66E-11
AC144548.1	0.155862	0.320425	1.039718	5.29E-07	1.38E-06
AC040160.1	0.409031	0.841324	1.040451	5.42E-07	1.41E-06
AC211476.2	0.094405	0.296701	1.652074	1.31E-05	2.78E-05
AC131097.1	0.013955	0.097258	2.80108	8.84E-05	0.000166
AP000753.2	0.055287	0.32135	2.53914	0.024175	0.031344
LINC01888	0.083524	0.030333	-1.46129	2.12E-06	5.06E-06
AL358216.1	0.015035	0.146427	3.28374	0.037385	0.047049
STPG3-AS1	0.086994	0.401415	2.206098	1.70E-09	6.21E-09
PCAT7	0.091977	0.547294	2.572977	3.72E-16	3.50E-15
AL390729.1	0.041812	0.301221	2.848818	6.00E-06	1.34E-05
AL022316.1	0.152942	0.581739	1.927386	1.14E-10	4.95E-10
LINC00987	1.750354	0.579073	-1.59583	1.52E-26	7.44E-25
SAP30L-AS1	0.279939	0.657096	1.230993	0.000593	0.000991
AC090796.1	0.934712	0.154372	-2.59811	1.96E-23	5.10E-22
AL606760.3	0.00688	0.016042	1.221354	0.000604	0.001009
AC245884.8	0.346304	0.815793	1.236165	8.75E-08	2.56E-07
ASH1L-IT1	0.02474	0.286289	3.532537	0.000978	0.00158
AC010735.1	0.01826	0.19667	3.429016	3.27E-07	8.76E-07
C12orf80	0.018761	0.082784	2.141625	1.79E-07	4.97E-07
AC138207.4	0.56142	1.244638	1.148574	6.84E-13	4.04E-12
AL121852.1	0.038589	0.16626	2.107185	9.42E-12	4.81E-11
AL391152.1	0.035768	0.182725	2.352928	0.002492	0.003776
AC007014.2	0.134709	0.609729	2.178322	8.77E-15	6.75E-14
AC135895.1	0.023505	0.006041	-1.96006	6.83E-11	3.07E-10
AC020907.4	0.791722	1.85806	1.230731	2.68E-07	7.27E-07
AL606970.1	0.005136	0.135755	4.724217	6.60E-05	0.000127
AC144450.1	0.04822	0.331484	2.781247	2.25E-09	8.02E-09
LINC01785	0.38486	0.128467	-1.58294	3.17E-34	1.64E-31
LINC00958	0.174741	1.042715	2.577053	0.025226	0.032589
LINC01213	0.009575	0.266979	4.801364	1.58E-10	6.74E-10

CYP4F26P	0.082266	0.264202	1.683273	0.001544	0.002413
AC127496.1	0.024215	0.056306	1.2174	0.018833	0.024827
AC141930.1	0.07875	0.569903	2.855361	7.09E-10	2.74E-09
LINC00544	0.01271	0.030815	1.277718	0.000163	0.000293
AP003392.3	0.121687	0.256815	1.077554	1.12E-05	2.41E-05
AP000721.2	0.006428	0.045163	2.812784	8.24E-08	2.42E-07
AC092162.2	0.004409	0.015495	1.813272	0.027018	0.034754
DDX11-AS1	0.059002	0.356688	2.595822	1.83E-30	2.64E-28
LINC00659	0.017319	0.277413	4.001626	1.59E-06	3.87E-06
AC139713.2	0.144319	0.053642	-1.42782	5.42E-16	4.95E-15
AC091114.1	0.011577	0.038752	1.742983	0.000116	0.000214
AC093484.1	0.172188	0.084215	-1.03184	5.50E-08	1.66E-07
Z82246.1	0.630555	0.063564	-3.31035	3.13E-29	3.09E-27
AC093281.2	0.004638	0.224106	5.594605	1.29E-11	6.47E-11
AL589765.7	0.355901	0.162807	-1.12831	2.70E-05	5.45E-05
LINC00111	0.026875	0.06585	1.292935	9.72E-05	0.000182
AL133320.1	0.532866	0.13702	-1.95938	1.64E-10	6.96E-10
SRGAP3-AS2	7.202714	1.532895	-2.23228	9.90E-13	5.74E-12
BBOX1-AS1	0.030951	1.572359	5.666804	5.25E-21	9.35E-20
LINC00941	0.068878	0.67699	3.297028	9.95E-05	0.000186
AL354732.1	0.07079	0.171308	1.274976	5.85E-10	2.29E-09
PACRG-AS1	0.068061	0.016315	-2.06059	5.01E-17	5.41E-16
AC091173.1	0.006482	0.052979	3.030811	0.006219	0.00885
AC008734.1	0.029884	0.081494	1.447311	5.75E-05	0.000111
AC108058.1	0.062922	0.401881	2.675141	1.23E-12	7.07E-12
AL109615.2	0.048035	0.12802	1.414201	0.037445	0.047106
AL139300.2	0.236727	0.104904	-1.17416	4.54E-16	4.18E-15
LINC00519	0.079544	0.473332	2.573026	2.67E-16	2.57E-15
AL359538.1	0.011784	0.031716	1.428392	0.033961	0.043025
AL050320.1	0.051917	0.141726	1.44883	0.006441	0.009152
AC026904.2	0.12094	0.040536	-1.57701	3.52E-13	2.14E-12
AC131159.1	0.133894	0.292922	1.129428	0.001669	0.002597
AP003680.1	0.008817	0.033887	1.942437	2.30E-15	1.95E-14
EXTL3-AS1	0.047433	0.158683	1.742187	6.90E-11	3.10E-10
NCF4-AS1	0.022953	0.055705	1.279115	0.034984	0.044188
MIR3945HG	5.205868	0.497507	-3.38735	6.84E-31	1.17E-28
AC092757.2	0.171365	0.381438	1.154373	5.91E-05	0.000114
MIS18A-AS1	0.110601	0.326224	1.560501	9.76E-11	4.30E-10
MAFA-AS1	0.036297	0.876967	4.59461	3.32E-11	1.57E-10
AC083906.3	0.03702	0.203788	2.460688	1.51E-07	4.25E-07
AC015468.3	0.120635	0.026234	-2.20112	4.15E-14	2.91E-13
LBX2-AS1	2.106965	4.564234	1.115207	5.16E-16	4.73E-15
LNCOC1	0.032042	0.121105	1.918216	6.71E-13	3.97E-12
AC011365.2	0.1364	0.047183	-1.5315	1.68E-16	1.67E-15

MAGI2-AS3	3.156912	1.170714	-1.43113	4.30E-26	1.95E-24
AC025539.1	0.024722	0.108537	2.134348	6.74E-06	1.49E-05
CLDN10-AS1	0.020316	2.072484	6.672586	3.51E-17	3.87E-16
AC006946.3	0.02401	0.144159	2.585952	1.49E-07	4.21E-07
LINC00891	0.261706	0.054607	-2.26078	4.02E-28	2.84E-26
TTC3-AS1	0.035027	0.186411	2.41196	1.69E-11	8.38E-11
AC022400.2	0.035637	0.147043	2.044806	0.001522	0.002382
AC009262.1	0.000973	0.147116	7.240541	5.21E-12	2.75E-11
AL360093.1	0.032551	0.126413	1.957385	0.005114	0.007375
AC107959.3	0.186751	0.975462	2.384973	1.05E-19	1.63E-18
HIF1A-AS2	0.310293	2.311999	2.897439	9.60E-07	2.41E-06
AC015660.2	0.892392	0.333662	-1.41929	2.99E-06	6.95E-06
LINC01356	0.028223	0.149925	2.409314	0.00012	0.000221
AC023090.1	0.059612	0.455548	2.933918	8.60E-08	2.52E-07
AL109741.1	2.578332	0.487915	-2.40174	2.16E-29	2.17E-27
C8orf31	0.321629	1.013762	1.65625	1.83E-09	6.65E-09
AL353801.1	0.056002	0.232432	2.053266	0.002182	0.003333
AL031728.1	0.111378	0.277403	1.31652	3.63E-09	1.26E-08
AL589765.1	0.050977	0.23457	2.202092	0.000135	0.000246
ATP6V1B1-AS1	0.015579	0.059671	1.937442	0.000462	0.000782
AC007249.2	0.365679	0.813433	1.153448	1.19E-09	4.44E-09
AL109811.1	0.054154	0.119092	1.136947	2.74E-08	8.60E-08
AL365356.4	0.032419	0.817453	4.65621	9.82E-23	2.27E-21
RMST	0.259215	0.084776	-1.61242	7.67E-20	1.21E-18
CRTC3-AS1	0.12594	0.297621	1.240743	2.35E-10	9.72E-10
AC011503.1	0.078095	0.235885	1.59479	0.000319	0.000551
C20orf197	0.114964	1.052337	3.194347	6.54E-11	2.95E-10
AL354707.2	0.023127	0.088621	1.938086	1.28E-06	3.15E-06
AC106037.1	0.156609	0.394935	1.334447	1.04E-08	3.45E-08
AC005387.2	0.182619	0.423712	1.214245	7.90E-10	3.04E-09
CACNA1G-AS1	0.027642	0.074252	1.425547	8.05E-05	0.000152
AC109454.3	0.01443	0.264956	4.198569	1.50E-06	3.64E-06
AC022784.6	0.035556	0.2482	2.803336	2.65E-11	1.28E-10
AC022148.2	0.017184	0.064302	1.903842	3.20E-06	7.40E-06
AC009120.5	0.026611	0.084001	1.658403	6.10E-06	1.36E-05
AC005757.1	0.026246	0.122881	2.22708	0.006806	0.009615
TBX5-AS1	4.262145	1.31053	-1.70143	4.87E-26	2.13E-24
MYCNOS	0.00514	0.087932	4.096656	1.04E-07	2.99E-07
AL583810.2	0.032206	0.083515	1.374705	0.000496	0.000837
AL021392.1	0.376418	0.820921	1.124908	1.85E-09	6.71E-09
AC073283.2	0.05115	0.163269	1.674443	0.000152	0.000275
AC005899.1	0.077571	0.189538	1.288906	0.00043	0.000731
AP000350.7	0.013229	0.040136	1.601187	5.88E-10	2.30E-09
AC012213.2	0.01104	0.029359	1.411126	8.47E-05	0.00016

AC245100.1	0.022246	0.055434	1.317199	2.10E-06	5.01E-06
AL354793.1	0.030451	0.097151	1.673752	0.0037	0.005456
AC097717.1	0.004794	0.016832	1.81192	1.12E-06	2.78E-06
AC105053.1	0.301857	0.13575	-1.15291	3.88E-16	3.64E-15
LINC01918	0.019575	0.040997	1.066541	0.021355	0.027937
AC092171.3	0.45323	0.993852	1.132789	9.12E-05	0.000171
RDH10-AS1	0.116955	0.535073	2.193789	1.22E-05	2.61E-05
AC016044.1	0.001954	0.483099	7.95	4.69E-11	2.17E-10
PIK3CD-AS2	1.020531	3.614771	1.824585	7.88E-14	5.25E-13
AC245060.6	0.081994	0.240759	1.554004	6.87E-13	4.05E-12
STEAP3-AS1	0.058435	0.162265	1.47344	2.02E-07	5.59E-07
AL138902.1	0.017603	0.13241	2.911116	2.19E-14	1.60E-13
AC091153.2	0.024091	0.098643	2.033744	4.62E-05	9.05E-05
AL353768.1	0.035367	0.120751	1.771558	0.000903	0.001465
LINC01913	0.021854	0.188731	3.110338	0.000112	0.000208
AL139383.1	0.220521	0.100945	-1.12734	9.26E-17	9.58E-16
FALEC	0.096391	0.284975	1.563867	1.34E-11	6.68E-11
APCDD1L-DT	0.016105	0.147934	3.199377	7.92E-08	2.34E-07
LINC02345	2.015321	0.847536	-1.24966	4.84E-14	3.35E-13
AL445423.1	0.967875	0.191592	-2.33678	4.88E-25	1.73E-23
AL133255.1	0.035474	0.121419	1.775143	0.000117	0.000215
TCEAL3-AS1	0.033436	0.26666	2.995539	0.000511	0.00086
AC008758.2	0.02741	0.079383	1.534126	0.007636	0.010714
AC097532.2	0.013309	0.039482	1.568826	4.89E-06	1.10E-05
AC010319.4	0.193624	0.435983	1.171017	1.25E-11	6.30E-11
AC027338.1	0.052077	0.126815	1.284019	0.007348	0.010342
AC010735.2	0.230428	1.06765	2.212048	3.82E-05	7.56E-05
AL391095.3	0.067131	0.134392	1.001386	0.030766	0.039243
LINC02473	0.002724	0.073185	4.747843	4.10E-18	5.18E-17
HCFC1-AS1	0.07648	0.211418	1.46694	6.76E-05	0.000129
AC027031.2	0.44227	1.411978	1.674718	3.14E-09	1.10E-08
DCST1-AS1	0.544073	1.952514	1.843461	6.99E-24	1.94E-22
AC011405.1	0.045462	0.19148	2.074466	0.008804	0.01222
AP002336.2	0.208605	0.592934	1.507095	3.20E-10	1.29E-09
AC079340.2	0.015395	0.045811	1.573263	0.000104	0.000194
AL049874.3	0.008923	0.113284	3.666236	9.33E-10	3.54E-09
AC068580.1	0.768331	1.821999	1.245722	1.06E-09	4.00E-09
SENCR	1.289903	0.537514	-1.26289	4.41E-23	1.08E-21
AC121757.1	0.589505	1.517978	1.364575	4.72E-06	1.07E-05
AC025580.1	0.301615	5.817253	4.269556	6.03E-24	1.73E-22
AC226118.1	0.154525	0.424486	1.457876	0.009664	0.013336
AC244453.1	0.025827	0.065797	1.349147	0.000752	0.001234
AC007938.2	0.024644	0.08683	1.816953	5.56E-09	1.90E-08
AP003419.3	0.310423	0.648966	1.063908	5.87E-07	1.51E-06

AP000253.1	0.015508	0.054233	1.806145	4.05E-11	1.89E-10
AC008121.2	0.09998	0.208563	1.060766	0.033447	0.042428
HCG15	0.198102	0.590676	1.57612	6.50E-14	4.40E-13
MIR4435-2HG	1.218079	2.759324	1.179707	1.48E-19	2.23E-18
AL121652.1	0.039676	0.315469	2.991164	3.08E-05	6.16E-05
LINC00319	0.002002	0.016445	3.037849	1.79E-06	4.34E-06
LINC02381	1.962675	4.538867	1.209511	0.03151	0.040135
AC026401.3	2.833622	7.864198	1.472652	1.47E-25	6.10E-24
AC025271.4	0.55039	0.137881	-1.99703	2.29E-24	7.18E-23
AP001528.2	1.05182	0.473836	-1.15043	5.99E-20	9.57E-19
AC005996.1	0.017595	0.224834	3.675638	1.09E-07	3.15E-07
AP003352.1	1.089431	2.294235	1.074439	2.50E-12	1.37E-11
DEPDC1-AS1	0.00706	0.040208	2.509751	2.03E-09	7.32E-09
AC073957.1	0.018328	0.137197	2.904135	6.85E-06	1.51E-05
AC109361.2	0.666814	0.314256	-1.08534	1.34E-14	1.01E-13
LINC02104	0.614965	0.148526	-2.04979	1.76E-28	1.44E-26
AL109917.1	0.192233	0.388199	1.013942	1.27E-05	2.71E-05
AC035139.1	0.14387	0.395078	1.457371	0.029233	0.037449
AC133961.1	0.058228	0.129284	1.150748	0.000388	0.000663
LINC01594	0.043053	0.205201	2.252836	0.00097	0.001568
AC005537.1	0.010712	0.151585	3.822834	6.03E-07	1.55E-06
AC008635.1	0.055105	0.121926	1.145736	0.002692	0.004059
AL031600.2	0.031035	0.143846	2.21256	0.00109	0.001749
LINC01234	0.006271	0.310649	5.630537	4.99E-10	1.97E-09
AC016597.1	0.016274	0.060993	1.906084	0.001428	0.002247
AC116552.1	0.111633	0.310506	1.475862	0.000111	0.000207
AC018809.1	0.183947	0.508495	1.466939	4.07E-09	1.41E-08
C8orf34-AS1	8.883668	3.697244	-1.26471	1.86E-18	2.46E-17
AP001469.3	0.340622	0.955346	1.487853	8.77E-16	7.81E-15
U47924.1	0.049474	0.266401	2.428849	8.52E-14	5.65E-13
AC109809.1	0.06931	0.142926	1.044127	0.027463	0.035298
AL356270.1	0.023172	0.086245	1.896036	0.00042	0.000716
AC007494.2	0.023083	0.301066	3.705162	0.026304	0.033893
AC147651.1	0.024175	0.07707	1.672661	0.029485	0.03771
FOXCUT	0.051844	0.299207	2.528907	7.85E-06	1.72E-05
AL121895.1	0.039985	0.145256	1.861065	0.039702	0.049724
AC090515.5	0.033831	0.131679	1.960621	0.00946	0.013075
SBF2-AS1	0.526166	1.457126	1.469535	1.92E-27	1.13E-25
AC104836.1	0.313156	0.135937	-1.20394	3.87E-15	3.16E-14
LINC02454	0.028252	0.240625	3.090368	0.000632	0.001049
AP002360.3	0.343895	0.982038	1.513811	2.97E-10	1.21E-09
LINC00342	2.179967	9.596258	2.138165	2.24E-05	4.59E-05
AC068025.1	0.04588	0.224484	2.290672	2.80E-15	2.35E-14
AC004585.1	0.452926	1.183981	1.386297	3.02E-11	1.44E-10

AC099518.1	0.140794	0.719221	2.352851	2.87E-13	1.77E-12
LINC01055	0.059195	0.143764	1.280163	0.000503	0.000849
AC008937.2	0.028466	0.317776	3.480714	0.036297	0.045763
ERVE-1	0.951512	0.447106	-1.08961	8.92E-17	9.24E-16
AL138921.1	0.128788	0.264179	1.036521	2.71E-08	8.52E-08
AC068473.3	0.037784	0.086202	1.189932	0.012396	0.016797
C6orf99	0.487139	1.148374	1.237186	1.65E-10	7.00E-10
AC114956.2	0.115059	0.250507	1.122477	6.71E-05	0.000128
RHPN1-AS1	0.431064	1.409891	1.709609	1.64E-19	2.47E-18
AL022724.1	0.100961	0.290781	1.52614	0.014536	0.019477
LINC00114	0.025915	0.011605	-1.15903	3.81E-10	1.53E-09
AC026992.1	0.663062	0.143491	-2.20818	4.62E-24	1.37E-22
AC011815.2	0.062735	0.170875	1.445591	1.34E-05	2.85E-05
AP000446.1	0.013721	0.033052	1.268375	0.000539	0.000906
AFAP1-AS1	0.135049	11.84695	6.454888	5.64E-22	1.13E-20
LINC02081	0.135611	1.122754	3.0495	3.04E-16	2.90E-15
AC022148.1	0.015147	0.182637	3.59192	1.38E-05	2.91E-05
CARS-AS1	0.013226	0.054936	2.054353	0.002286	0.003482
AL079303.1	0.05043	0.561805	3.477707	1.12E-19	1.72E-18
XXYLT1-AS1	0.030739	0.138592	2.172709	7.14E-05	0.000136
LINC00165	0.522192	0.096396	-2.43754	5.56E-25	1.95E-23
LHFPL3-AS1	0.148253	0.053485	-1.47085	6.82E-19	9.60E-18
AC122710.3	0.024014	0.213388	3.151557	7.64E-14	5.12E-13
AL357079.1	0.340246	0.736201	1.113524	1.70E-11	8.40E-11
FO680682.1	0.033096	0.246885	2.899104	2.33E-08	7.40E-08
AC021491.2	0.028387	0.110542	1.961281	1.00E-08	3.33E-08
AC004543.1	0.037727	0.114686	1.604031	1.50E-05	3.15E-05
AC010834.3	0.702125	1.573474	1.164153	3.09E-07	8.30E-07
LINC00305	0.026853	0.0126	-1.09162	0.000366	0.000628
AC025174.1	0.19418	0.068417	-1.50496	1.10E-07	3.17E-07
AC074135.1	0.185488	0.871196	2.231674	0.003811	0.005606
AC023301.1	0.011861	0.272213	4.520382	1.27E-17	1.50E-16
C1orf220	0.056868	0.306967	2.432398	1.09E-22	2.49E-21
IRAIN	0.098615	0.282146	1.516558	1.15E-06	2.84E-06
AC239809.3	0.018813	0.044237	1.233535	0.002754	0.004144
AC023825.2	0.074359	0.247466	1.734659	2.29E-05	4.68E-05
TNKS2-AS1	0.262022	0.541072	1.046134	4.82E-06	1.09E-05
AL161729.4	0.126757	0.254606	1.006196	0.00024	0.000423
AC100791.3	0.035182	0.100691	1.517016	0.015275	0.020387
AC011899.3	1.317664	0.325141	-2.01885	3.99E-25	1.45E-23
AC243965.2	0.032359	0.076822	1.247339	2.17E-05	4.45E-05
AC093520.1	0.015128	0.072682	2.264422	9.60E-08	2.79E-07
AC106795.3	0.091032	0.034965	-1.38045	7.65E-09	2.58E-08
AC100827.4	0.016386	0.039553	1.271294	0.039013	0.048939

AC003985.2	0.208031	0.066309	-1.64953	1.13E-11	5.71E-11
AC020659.1	0.086349	0.279474	1.694462	1.88E-05	3.90E-05
OR7E11P	0.162008	0.04098	-1.98307	5.96E-20	9.56E-19
LINC01537	0.279291	0.1039	-1.42658	4.69E-23	1.13E-21
AC069360.1	0.744177	0.191183	-1.96069	6.34E-25	2.20E-23
LINC00649	0.329694	0.758045	1.201157	6.53E-07	1.67E-06
LINC01588	0.126707	0.327279	1.36902	7.31E-20	1.16E-18
SPATA3-AS1	0.004623	0.029342	2.665995	1.14E-09	4.26E-09
AC122134.1	0.001351	0.123641	6.515594	1.62E-10	6.87E-10
AC074366.1	0.010355	0.075147	2.859336	0.003053	0.004562
AP006284.1	1.333177	2.712039	1.02451	0.012396	0.016797
AL031985.3	0.367852	1.467183	1.995853	5.59E-30	6.32E-28
AL022323.3	0.003426	0.021572	2.65454	0.001433	0.002254
AL353747.3	0.540484	0.223342	-1.275	2.42E-08	7.66E-08
AC008011.2	0.178509	0.058114	-1.61905	1.32E-18	1.77E-17
AC110285.1	0.100901	0.258817	1.358989	6.52E-09	2.22E-08
AL390198.1	0.709543	2.111819	1.573524	4.51E-06	1.02E-05
AL132800.1	0.23767	0.556351	1.227038	2.40E-09	8.54E-09
AC092338.1	0.154784	0.51649	1.738481	0.016519	0.021944
AZIN1-AS1	0.542226	1.111675	1.035769	1.84E-10	7.72E-10
LINC01659	0.093237	0.44129	2.242746	0.000469	0.000794
AC004584.1	0.052569	0.204417	1.95922	4.97E-13	2.98E-12
AC080023.1	0.056476	0.14283	1.338589	8.97E-05	0.000169
AC004584.3	0.041424	0.08291	1.001074	0.010004	0.013757
AC096637.2	0.58138	0.179244	-1.69756	3.05E-05	6.10E-05
AC007743.1	1.319561	0.415754	-1.66626	2.30E-23	5.87E-22
DANT2	0.052369	0.40402	2.947654	0.033966	0.043025
AGBL1-AS1	0.03891	0.015495	-1.32834	4.72E-26	2.08E-24
AC092078.1	0.106538	0.011427	-3.22083	6.73E-38	8.36E-35
FNDC1-IT1	0.008378	0.112418	3.746107	2.48E-11	1.20E-10
LINC01185	0.01659	0.058382	1.815243	8.70E-07	2.20E-06
AC010978.1	0.018848	0.133837	2.827976	0.008015	0.011211
AL353693.1	0.009359	0.65578	6.13068	4.44E-17	4.82E-16
AC087741.1	0.471056	1.560282	1.727837	1.29E-14	9.67E-14
LINC02159	0.04575	0.876506	4.259929	2.18E-18	2.85E-17
AC036214.1	0.02363	0.084708	1.841858	0.003307	0.004915
AP003469.2	0.129854	1.007983	2.956513	1.08E-21	2.12E-20
AC026469.1	0.069791	0.031018	-1.16993	9.35E-17	9.65E-16
AC069209.1	0.544024	0.212912	-1.35341	1.07E-18	1.47E-17
AC008991.1	0.052772	0.017586	-1.58534	6.90E-07	1.76E-06
TM4SF1-AS1	0.210941	0.775048	1.877447	5.09E-09	1.75E-08
AL357146.1	0.100887	0.015675	-2.68616	2.72E-27	1.56E-25
AL161804.1	0.022537	0.069084	1.616081	3.49E-05	6.92E-05
AC007249.1	0.165146	0.603961	1.870716	1.41E-08	4.58E-08

KCNQ1DN	0.025895	0.011012	-1.23352	4.92E-05	9.60E-05
LINC01973	0.004061	0.008184	1.011015	0.010381	0.014238
AL445685.1	0.023095	0.08746	1.921012	0.029267	0.037486
AC234778.2	0.037783	0.143674	1.926988	0.01478	0.019785
AL583722.1	0.048926	0.166268	1.764846	1.19E-07	3.40E-07
CASC19	0.023735	0.627508	4.72456	0.000164	0.000296
AP000845.1	0.048617	0.10849	1.158026	1.32E-07	3.74E-07
AC097537.1	0.096099	0.022304	-2.10721	4.03E-39	8.36E-36
LMO7DN-IT1	0.679636	0.30432	-1.15918	4.64E-10	1.83E-09
SLX1A-SULT1A3	0.010326	0.030099	1.543449	2.64E-09	9.39E-09
AL109914.1	0.105219	0.65514	2.638413	3.62E-09	1.26E-08
AC010864.1	0.140927	0.336407	1.255255	8.06E-15	6.24E-14
LINC01524	0.001234	0.018745	3.925334	2.04E-06	4.88E-06
LINC00461	0.001639	0.042263	4.68874	2.91E-17	3.24E-16
LINC01510	0.008829	0.151829	4.104045	7.10E-06	1.57E-05
AC022445.1	0.0346	0.163731	2.242501	2.04E-06	4.88E-06
AC103736.1	0.244192	0.12104	-1.01254	5.10E-11	2.35E-10
AC008735.2	1.33641	2.753931	1.04313	1.97E-07	5.43E-07
LINC02041	0.941167	3.591536	1.932079	9.46E-05	0.000177
AC007365.1	0.134853	0.335613	1.31541	0.001253	0.001993
AL391832.2	0.233399	0.503323	1.108683	1.94E-09	7.03E-09
AC137894.1	0.174337	0.56711	1.701747	2.10E-05	4.32E-05
AL031717.1	0.218435	0.699589	1.679303	2.49E-10	1.03E-09
AC008063.2	0.014329	0.089387	2.641151	6.53E-09	2.22E-08
AC003681.1	0.082713	0.20346	1.298552	0.000206	0.000367
AC104695.3	0.657728	2.503512	1.92839	0.004873	0.007043
LINC02231	0.16433	0.046564	-1.81931	1.13E-13	7.35E-13
AL139035.1	0.074277	0.218919	1.559409	4.73E-12	2.52E-11
AL049646.1	0.006579	0.026521	2.01125	0.015519	0.020673
AC010997.4	0.073846	0.175557	1.249347	0.00101	0.001629
GS1-594A7.3	0.03352	0.297892	3.151696	2.89E-09	1.02E-08
AC091946.2	0.047799	0.199653	2.062433	0.001517	0.002375
AC016590.1	0.035774	0.125804	1.814211	0.00015	0.000272
AC125611.4	0.257054	0.0836	-1.6205	4.84E-09	1.67E-08
AL589743.4	0.012457	0.127976	3.360811	3.79E-06	8.67E-06
LINC01337	0.032812	0.089265	1.443893	2.73E-05	5.51E-05
AC083949.1	1.00169	0.446856	-1.16455	1.32E-16	1.33E-15
SMILR	0.024689	0.17127	2.794341	0.002174	0.003323
AC015689.1	0.106515	0.314132	1.56031	2.78E-05	5.60E-05
AC106795.2	0.156917	0.343759	1.131395	0.003208	0.004777
AL513327.2	0.038764	0.276597	2.834995	2.36E-13	1.48E-12
AL365194.1	0.064443	0.029515	-1.12655	0.000146	0.000265
AL162411.1	0.080547	0.495022	2.619583	4.55E-06	1.03E-05
AC239803.3	0.04103	0.269448	2.715268	9.31E-16	8.22E-15

LINC02068	0.021598	0.074841	1.792916	3.91E-05	7.72E-05
AL137786.1	0.096815	0.037756	-1.3585	9.67E-07	2.43E-06
AP001021.2	0.122898	0.315791	1.361509	0.000788	0.001289
AC099066.2	0.030708	0.280565	3.19165	8.30E-21	1.44E-19
AC069120.1	0.02259	0.458757	4.344006	3.14E-05	6.26E-05
AC004707.1	0.080106	0.228195	1.510289	4.38E-08	1.34E-07
AL358072.1	0.155187	0.317147	1.031143	0.000117	0.000215
AC108134.2	0.445089	0.984219	1.144887	1.91E-12	1.07E-11
PCAT6	0.776601	5.629806	2.857839	3.38E-28	2.47E-26
Z82243.1	0.267302	0.767361	1.521434	2.15E-07	5.92E-07
AL161668.3	0.063576	0.167534	1.397898	2.72E-10	1.12E-09
LINC00844	0.422556	0.058155	-2.86116	2.85E-28	2.16E-26
PDXDC2P-NPIPB14P	0.61691	1.420761	1.203532	6.31E-09	2.15E-08
AC093535.1	0.185304	0.495971	1.420364	2.26E-05	4.63E-05
AL355488.1	0.679824	1.735782	1.352353	1.17E-09	4.37E-09
AL512656.1	0.133219	0.334195	1.326889	1.53E-05	3.20E-05
AC010998.3	1.426173	0.474297	-1.58829	3.54E-22	7.44E-21
AC024592.2	0.058303	0.338294	2.536629	7.69E-14	5.14E-13
AC011773.3	0.051638	0.116858	1.178245	7.30E-06	1.61E-05
RHOXF1-AS1	3.731158	1.282982	-1.54012	4.15E-19	6.02E-18
AC131097.3	0.055854	0.268683	2.266171	1.68E-07	4.69E-07
AC009686.2	0.326397	0.893859	1.45342	1.22E-11	6.13E-11
DPH6-DT	0.225535	0.112666	-1.0013	9.16E-15	7.03E-14
LINC00880	0.027618	0.331789	3.586595	6.12E-21	1.08E-19
LINC01572	0.019983	0.190689	3.254346	4.58E-29	4.44E-27
AC104561.3	0.049589	0.131071	1.402255	1.15E-07	3.31E-07
AC100826.1	0.019226	0.950254	5.627146	1.60E-05	3.35E-05
GRM5-AS1	0.002721	0.01694	2.638402	2.48E-07	6.76E-07
AL662844.3	0.292944	0.600873	1.036436	0.000137	0.000251
AL513218.1	0.304506	0.811288	1.413743	7.84E-09	2.63E-08
AC099811.1	0.027122	0.238656	3.137421	0.005459	0.007837
ZNF793-AS1	0.418559	1.213027	1.535108	1.59E-09	5.83E-09
LINC01224	0.089989	0.611429	2.764366	0.00128	0.002032
LINC02122	0.016825	0.572418	5.088429	1.31E-10	5.66E-10
AL049539.1	0.028682	0.445059	3.955757	5.17E-16	4.74E-15
AC022182.1	0.043408	0.212962	2.294556	3.40E-11	1.60E-10
AC007991.3	0.029388	0.120455	2.035184	0.001318	0.002086
AC009065.5	0.223218	3.250209	3.864007	9.28E-22	1.82E-20
MIR3142HG	0.327094	0.920323	1.492433	1.53E-05	3.20E-05
LINC02182	0.010848	0.060248	2.473462	6.39E-06	1.42E-05
BARX1-DT	0.001619	0.393406	7.924721	1.23E-12	7.04E-12
AC017002.1	0.042611	0.228478	2.422746	0.000436	0.000741
AC103681.2	0.128195	0.063378	-1.0163	2.84E-09	1.00E-08
LINC01165	0.137265	0.028441	-2.27092	8.12E-23	1.91E-21

LINC00514	0.063294	0.143056	1.176437	0.014453	0.019378
FRGCA	0.047062	0.855032	4.183342	9.35E-18	1.13E-16
AC017033.1	0.082085	0.231335	1.494794	0.034291	0.043375
AP000553.2	0.095041	0.867322	3.189947	6.38E-19	9.03E-18
AL161757.2	0.044686	0.156888	1.811826	0.007517	0.010562
FOXD3-AS1	0.005244	1.111198	7.727223	1.78E-26	8.45E-25
TFAP2A-AS1	0.049028	0.456439	3.218735	3.24E-25	1.21E-23
AC112721.2	0.031859	0.409362	3.68361	7.24E-17	7.64E-16
LINC02048	0.0028	0.064484	4.525455	1.07E-06	2.67E-06
AC005699.1	0.024401	0.097717	2.001664	3.71E-05	7.35E-05
AC093690.1	0.085939	0.235652	1.455265	0.030909	0.039418
LEF1-AS1	0.025301	0.067091	1.40695	7.00E-12	3.64E-11
ITGA6-AS1	0.153788	0.407101	1.404441	1.92E-12	1.07E-11
AC103858.2	0.031274	0.093422	1.578812	5.03E-05	9.80E-05
FOXP4-AS1	0.346392	0.801895	1.211008	2.77E-05	5.57E-05
LINC01271	0.04758	0.307436	2.691867	3.65E-25	1.34E-23
AL157895.1	0.724855	0.292202	-1.31073	2.83E-18	3.65E-17
AL451074.2	0.076816	0.199231	1.37496	5.50E-10	2.16E-09
AC104316.2	0.022386	0.090748	2.019246	0.000313	0.000541
CERS6-AS1	0.00512	0.014088	1.460353	1.55E-06	3.77E-06
AL355303.1	0.021261	0.092596	2.122728	0.000916	0.001485
AC110619.1	0.131981	0.616542	2.223865	1.46E-06	3.57E-06
AC139887.2	0.560432	1.456404	1.377798	1.75E-16	1.73E-15
AC012456.2	0.021467	0.10385	2.274316	4.21E-06	9.60E-06
NKX2-1-AS1	2.715102	5.634702	1.053333	1.13E-06	2.81E-06
EPB41L4A-DT	1.245451	0.589581	-1.07891	2.18E-18	2.85E-17
AL008718.3	0.107125	0.340629	1.668909	2.00E-05	4.11E-05
AC145285.2	0.220811	0.477184	1.111737	9.00E-12	4.61E-11
AC004975.2	0.186164	0.444295	1.25494	9.44E-06	2.05E-05
SNHG12	2.356605	5.288872	1.16625	2.38E-13	1.48E-12
AC117382.2	0.006331	0.022039	1.799607	4.97E-06	1.12E-05
AC129510.2	0.048338	0.161933	1.744157	0.00033	0.000569
LINC00858	0.012917	0.47176	5.190737	2.31E-08	7.33E-08
AC133540.1	0.024241	0.201154	3.052766	7.15E-05	0.000136
AF117829.1	0.473727	1.029367	1.119631	3.00E-07	8.09E-07
AC034206.1	0.010827	0.179958	4.054914	6.85E-10	2.66E-09
DPYD-IT1	0.033009	0.174363	2.401175	0.029444	0.037681
AC005355.1	0.041659	0.118355	1.506424	0.000255	0.000447
AC027601.1	0.152255	0.338041	1.150707	1.01E-08	3.35E-08
AC015987.1	0.114541	0.319972	1.482083	0.000548	0.00092
AC008655.2	0.192147	0.457903	1.25283	5.04E-08	1.53E-07
AC079384.1	0.091824	1.317633	3.842926	0.0082	0.011441
MCM3AP-AS1	0.329631	0.697917	1.082203	1.31E-11	6.56E-11
AP005233.2	0.028867	2.062583	6.158869	5.17E-19	7.39E-18

SIDT1-AS1	0.04645	0.209475	2.173023	0.024477	0.031684
TH2LCRR	0.116772	0.335972	1.524645	0.000366	0.000629
LINC00460	0.115627	2.553053	4.464673	6.10E-12	3.19E-11
LINC01376	0.296035	0.687404	1.215391	1.46E-10	6.24E-10
AL139289.1	0.418365	0.991562	1.244939	8.31E-11	3.68E-10
LINC02280	0.008061	0.033535	2.056604	6.99E-12	3.64E-11
AC108134.1	0.372643	0.812922	1.125322	2.20E-06	5.22E-06
LINC01108	0.685455	0.338305	-1.01874	8.07E-07	2.05E-06
LINC01732	0.195692	0.074464	-1.39397	0.000159	0.000287
AC011287.1	0.009504	0.066076	2.797465	6.65E-05	0.000127
AC004477.1	0.071668	0.222974	1.637479	1.60E-14	1.18E-13
AC017076.1	0.101783	0.273459	1.425823	0.000589	0.000985
AL137793.1	0.181244	0.612136	1.755918	0.001904	0.00294
AC016924.1	0.565229	0.207389	-1.4465	7.89E-21	1.38E-19
AC089999.2	0.072191	0.472535	2.710535	1.81E-14	1.34E-13
AC006206.2	0.06473	0.645711	3.318392	0.000227	0.000402
UBE2R2-AS1	0.083444	0.280273	1.747957	0.0001	0.000187
AC005021.1	0.308149	0.762398	1.306915	0.000656	0.001087
AC114284.1	0.070977	0.150372	1.083117	4.53E-06	1.03E-05
AC017074.1	0.503179	1.19689	1.250148	1.60E-10	6.79E-10
AC010336.2	0.067197	0.163459	1.282458	4.04E-06	9.22E-06
FAM225B	0.029756	0.085197	1.517646	3.99E-10	1.59E-09
AL356417.2	0.068443	0.375415	2.455506	5.89E-14	4.02E-13
AL022067.1	0.210326	0.688163	1.710122	0.000331	0.000571
AC010328.1	0.004623	0.075257	4.024959	1.20E-12	6.91E-12
AC090409.1	0.348437	0.16349	-1.0917	7.66E-15	5.98E-14
AP001781.1	0.043227	0.196499	2.184509	0.002222	0.003389
AC007966.1	0.03465	0.097692	1.495382	0.029967	0.038319
AC010643.1	0.16364	0.58897	1.847667	0.000174	0.000312
AC012360.1	0.043877	0.113803	1.375012	0.000112	0.000208
LINC00624	0.051282	0.353668	2.785881	9.32E-19	1.28E-17
AC105398.1	0.070446	0.030317	-1.2164	3.56E-12	1.92E-11
LINC01891	0.024836	0.06836	1.460706	0.000233	0.000412
AC145285.3	0.111535	0.270335	1.277255	4.24E-07	1.12E-06
AC010168.2	0.3103	0.720158	1.214648	1.49E-07	4.20E-07
AP001628.1	0.180454	0.708271	1.972671	2.05E-09	7.38E-09
CU639417.4	0.00166	0.025206	3.924389	1.05E-11	5.34E-11
AL136528.1	0.058752	0.165311	1.492483	0.000103	0.000192
AC073842.2	0.256164	0.876917	1.77537	1.14E-14	8.61E-14
AC022784.1	0.056223	1.625497	4.853575	1.67E-21	3.20E-20
LINC01983	0.060497	0.928652	3.940209	1.73E-14	1.27E-13
AC012555.1	0.057032	0.189543	1.732687	4.62E-10	1.83E-09
LINC00884	0.256502	0.877088	1.773753	7.86E-16	7.05E-15
LINC01583	0.007244	0.040511	2.483535	1.80E-08	5.80E-08

AL136172.1	0.009025	0.027633	1.614425	9.16E-06	1.99E-05
AL442125.2	0.236082	0.525836	1.155324	0.01741	0.023029
AC112206.2	0.9271	0.16407	-2.49841	1.56E-28	1.31E-26
AC024361.3	0.170164	0.455946	1.421934	1.37E-08	4.46E-08
HIF1A-AS1	0.088973	0.186598	1.068493	0.004592	0.006667
AC104211.1	0.353548	0.052894	-2.74072	2.07E-23	5.35E-22
AC073573.1	0.021814	0.052889	1.277741	0.000143	0.000261
AC025171.3	0.242837	0.573601	1.240058	3.23E-05	6.42E-05
LINC00482	0.694863	1.801841	1.374671	1.27E-05	2.71E-05
AC007991.4	0.132736	0.50077	1.915592	0.000218	0.000387
LINC01270	0.14927	1.221912	3.033143	2.66E-28	2.06E-26
AC114956.1	0.107102	0.257532	1.265769	3.13E-05	6.24E-05
AL512353.1	0.300336	0.650067	1.114012	2.29E-05	4.69E-05
LINC00337	0.05204	0.445632	3.098156	9.01E-19	1.25E-17
LINC00115	0.219445	0.685291	1.642855	4.05E-19	5.89E-18
RBAKDN	0.061199	0.2813	2.200535	0.034099	0.043176
MIR181A1HG	0.016058	0.299262	4.220025	0.000751	0.001232
AC012213.1	0.002717	0.27164	6.643379	2.83E-09	9.98E-09
AC012146.3	0.058506	0.127807	1.127299	0.002273	0.003463
AC138393.3	0.12134	0.751774	2.631242	2.19E-16	2.14E-15
MHENCR	2.426738	5.115366	1.075819	6.84E-09	2.32E-08
AC008114.1	0.038474	0.113795	1.564499	0.012905	0.017457
AC113382.1	0.017209	0.036851	1.098557	0.00011	0.000203
AC010207.1	0.370003	0.125592	-1.5588	9.76E-18	1.17E-16
AL356299.2	0.092726	0.394851	2.090256	1.58E-16	1.57E-15
LINC02154	3.963764	0.430078	-3.2042	8.08E-30	8.81E-28
AC105219.1	0.069521	0.604901	3.12117	1.27E-13	8.22E-13
DRAIC	0.236652	3.906401	4.045003	0.001832	0.00284
AC008691.1	0.123297	0.303802	1.300999	0.009806	0.013511
RNF139-AS1	0.132294	0.387082	1.548891	8.78E-15	6.75E-14
LINC02003	0.003504	0.095117	4.762713	2.28E-18	2.97E-17
AP003717.1	0.190714	0.695796	1.867253	5.02E-08	1.52E-07
AC092384.1	0.12896	0.039927	-1.69149	1.03E-22	2.38E-21
AC011465.1	0.150194	0.462069	1.621283	0.001879	0.002906
AC010307.4	0.038262	0.296542	2.954237	1.68E-06	4.08E-06
AC106881.1	0.289796	0.141805	-1.03113	6.14E-17	6.56E-16
LINC02246	0.026521	0.103055	1.958231	2.30E-05	4.70E-05
AC131009.1	0.165734	0.524946	1.663299	1.51E-12	8.55E-12
AC108134.3	2.15127	0.872072	-1.30267	2.05E-19	3.06E-18
BMS1P4	0.140583	0.318424	1.179531	7.03E-11	3.15E-10
AC011676.1	0.142727	0.559193	1.970083	1.29E-14	9.71E-14
AC093151.2	0.219209	0.535633	1.288937	0.000149	0.00027
AC006017.1	0.137818	0.555759	2.011697	2.69E-11	1.30E-10
AC109460.2	0.567213	1.144199	1.012376	2.36E-08	7.48E-08

AC013264.1	7.094685	1.793646	-1.98384	3.32E-13	2.03E-12
LINC02202	0.21627	0.093902	-1.2036	2.48E-18	3.21E-17
LINC01287	0.007949	0.515889	6.020091	9.29E-06	2.02E-05
AC048382.5	0.490876	0.232902	-1.07564	8.09E-18	9.83E-17
AL121832.3	0.056957	0.299867	2.396387	3.80E-21	6.95E-20
LINC02197	0.040454	0.018284	-1.1457	1.01E-16	1.04E-15
AC107464.1	0.015636	0.063619	2.024588	0.009138	0.012662
AC008610.1	0.725212	1.948916	1.426196	2.91E-13	1.79E-12
LINC01116	0.316666	1.376561	2.120035	1.32E-05	2.80E-05
AL583722.3	0.009735	0.031401	1.689599	0.000109	0.000202
AP003500.1	0.006273	0.242948	5.275403	1.64E-09	6.00E-09
AC245060.5	0.207692	0.423672	1.028501	6.42E-06	1.42E-05
MAP3K20-AS1	0.092226	0.212181	1.202053	0.026245	0.033838
AC016737.1	0.113428	0.473087	2.060326	3.53E-16	3.34E-15
AC005281.1	0.112275	0.258869	1.20519	0.005742	0.008203
AC026333.4	0.10081	0.259094	1.361841	1.91E-06	4.62E-06
AC090579.1	0.266615	0.625505	1.230264	5.87E-07	1.51E-06
AL355076.2	0.038971	0.20994	2.42951	3.75E-15	3.08E-14
AC011462.4	0.505521	1.273792	1.333287	5.07E-10	2.00E-09
AC018552.3	0.022319	0.116741	2.386975	0.000746	0.001225
LINC01366	0.224434	0.10189	-1.13928	3.36E-16	3.18E-15
TMLHE-AS1	0.010955	0.049381	2.172423	2.18E-09	7.81E-09
AL450270.1	0.190151	0.052736	-1.85029	6.45E-14	4.38E-13
AC061975.1	0.002835	0.035268	3.636752	1.96E-12	1.09E-11
VCAN-AS1	0.026137	0.458244	4.131948	0.000129	0.000237
ZNF436-AS1	0.424952	0.882754	1.054714	1.03E-12	5.97E-12
AC010501.1	0.018525	0.048867	1.399418	0.000829	0.001354
AP003390.1	0.107276	0.555847	2.373356	1.00E-14	7.63E-14
AP001619.1	0.062062	0.260787	2.071085	6.70E-11	3.02E-10
LINC02588	0.008277	0.577452	6.124389	3.12E-08	9.74E-08
AC006064.3	0.064731	0.33073	2.353131	2.20E-16	2.14E-15
AC009269.4	0.121097	0.05576	-1.11886	4.34E-06	9.88E-06
AL136307.1	0.057065	0.273802	2.262457	4.14E-08	1.27E-07
LINC01634	0.013041	0.052508	2.00952	0.000605	0.00101
AL049747.1	0.017484	0.052792	1.594295	0.001376	0.002173
AL354989.1	0.221565	0.594906	1.424932	8.12E-09	2.72E-08
LINC01506	1.007719	0.195781	-2.36378	1.51E-27	9.18E-26
AC010271.2	0.01718	0.071543	2.0581	2.35E-07	6.45E-07
AC007663.2	0.186257	0.080184	-1.21592	5.54E-13	3.29E-12
AC006435.2	0.573402	1.187418	1.050211	1.14E-05	2.45E-05
LINC00578	0.788013	2.14358	1.443731	2.81E-07	7.60E-07
LINC01344	0.005657	0.045945	3.021908	3.68E-08	1.14E-07
CNTN4-AS1	0.036507	0.018221	-1.00257	8.22E-07	2.08E-06
C1orf140	0.054937	0.010297	-2.41551	4.47E-26	2.01E-24

LINC02373	0.035143	0.079589	1.179314	0.008719	0.012108
AC244100.2	0.009693	0.004626	-1.06719	1.03E-05	2.22E-05
LINC02036	0.262499	1.121197	2.094658	4.15E-08	1.27E-07
AL024508.2	0.161522	0.713051	2.142279	4.19E-17	4.59E-16
CDKN2A-DT	0.007974	0.08591	3.429457	5.37E-07	1.40E-06
AC100771.2	0.021571	0.092644	2.102612	6.45E-11	2.91E-10
AC016575.1	0.030012	0.096476	1.684611	2.42E-05	4.92E-05
LINC00824	0.007785	0.151585	4.283351	0.001939	0.00299
AC020916.2	0.093374	0.262243	1.489806	0.001961	0.003023
Z98885.3	0.10628	0.253454	1.253861	0.005218	0.00752
AC090125.1	0.005742	0.158311	4.785054	2.66E-09	9.42E-09
LINC01616	0.109707	0.012521	-3.13121	1.51E-25	6.22E-24
SNHG3	2.575382	8.341415	1.695506	5.59E-21	9.93E-20
AL121829.2	0.040369	0.150248	1.896032	1.98E-06	4.76E-06
LINC02580	0.343151	0.147987	-1.21337	2.17E-17	2.47E-16
AC026356.1	0.490574	1.083948	1.143752	3.14E-09	1.10E-08
AC104667.2	0.563797	1.195886	1.084834	9.70E-10	3.67E-09
AP001893.1	0.234062	0.505028	1.109472	0.000291	0.000506
AC021755.3	0.068154	0.137196	1.009361	0.0023	0.003501
AC243830.1	0.018533	0.278455	3.909302	3.55E-15	2.93E-14
AL139412.1	0.022719	0.500491	4.461375	1.11E-20	1.89E-19
AC011498.6	0.260924	0.56074	1.103702	1.72E-06	4.16E-06
AC027281.1	1.32626	0.308556	-2.10376	1.19E-09	4.44E-09
LINC01730	0.286363	0.694123	1.277347	0.01735	0.02296
AC012640.2	0.740052	2.271779	1.618124	7.93E-19	1.11E-17
AC021218.1	0.039347	0.285746	2.860388	7.54E-05	0.000143
AC128709.1	0.255031	0.101288	-1.33222	1.22E-18	1.65E-17
AC012377.1	0.005551	0.011454	1.0452	0.001296	0.002054
AL513365.2	0.27991	0.587505	1.069639	0.006175	0.00879
FLJ42969	0.02635	0.115628	2.133593	6.32E-05	0.000122
AL138889.1	0.001433	0.00398	1.474086	0.010086	0.013864
AP002856.2	2.008729	0.127822	-3.97407	1.33E-13	8.58E-13
AC025171.4	0.541375	1.154851	1.093007	0.000225	0.000399
AC016885.2	0.310836	0.047025	-2.72464	1.04E-26	5.28E-25
PARD3-AS1	0.235607	0.602962	1.355682	5.02E-06	1.13E-05
LINC02418	0.002783	0.706675	7.98813	0.000151	0.000273
AL355075.2	0.54507	1.115704	1.033441	6.33E-05	0.000122
AC002066.1	0.974452	0.300981	-1.69492	1.23E-24	4.07E-23
AL513327.3	0.148525	0.464924	1.646287	0.000459	0.000777
MATN1-AS1	0.226904	0.482633	1.088841	2.33E-11	1.13E-10
AC092718.1	0.027331	0.057231	1.066283	0.01244	0.016854
LINC00163	0.454798	0.04683	-3.27972	1.16E-30	1.86E-28
AC009955.4	0.088405	0.203699	1.204241	0.008597	0.011954
AC093607.1	0.100129	0.025899	-1.95091	5.74E-10	2.25E-09

RAMP2-AS1	0.913051	0.297458	-1.61801	4.18E-24	1.26E-22
AL513327.1	0.506119	1.155684	1.191199	1.45E-05	3.05E-05
CU634019.6	0.006246	0.022904	1.874637	2.93E-06	6.81E-06
AP000688.1	0.103118	0.288343	1.483495	0.000153	0.000276
AL360270.3	0.083731	0.186742	1.157209	2.49E-10	1.03E-09
AC011298.1	0.002049	0.323856	7.303975	5.57E-10	2.18E-09
AC004551.1	0.023075	0.10062	2.124482	0.000715	0.001176
AL731533.2	0.126757	0.642337	2.341264	1.62E-16	1.61E-15
STAM-AS1	0.069519	0.189777	1.448812	5.88E-11	2.67E-10
AC099811.5	0.029567	0.205009	2.793608	0.039839	0.049885
AC087521.1	0.926068	0.167593	-2.46615	3.47E-22	7.35E-21
AC083967.1	0.030832	0.216248	2.810184	5.48E-05	0.000106
AC139887.1	0.472598	1.450854	1.618217	4.18E-11	1.95E-10
AP000302.1	0.02599	0.065542	1.334466	0.001898	0.002932
DSCAS	0.049148	0.122252	1.314646	7.53E-06	1.66E-05
AC068631.1	0.004224	0.016388	1.955845	9.28E-12	4.75E-11
LINC02031	0.123421	0.059537	-1.05172	3.24E-06	7.47E-06
LINC00973	0.024507	1.978517	6.33506	4.52E-08	1.38E-07
AC007255.1	0.129767	0.399259	1.621397	0.004793	0.006936
AC023355.1	0.048679	0.101005	1.053055	1.75E-06	4.24E-06
LINC01704	0.014889	0.03194	1.101075	0.000767	0.001256
TRMT2B-AS1	0.058919	0.127725	1.116228	0.01538	0.020523
LINC01971	0.006382	0.095837	3.90847	3.87E-13	2.34E-12
AC125807.2	3.027995	0.747481	-2.01825	5.67E-28	3.83E-26
AC244197.2	0.308715	0.667089	1.111602	0.00178	0.002766
BCAR4	0.006478	0.778133	6.908313	2.96E-05	5.93E-05
LINC02535	0.011289	0.256741	4.507354	5.40E-24	1.58E-22
AC020907.1	0.012477	0.920209	6.204609	1.29E-27	8.00E-26
AC016738.2	0.009803	0.057396	2.5496	1.09E-06	2.71E-06
AC091212.1	0.037631	0.213485	2.504156	6.29E-06	1.40E-05
AC010331.1	0.131171	0.420339	1.680102	4.92E-10	1.94E-09
AC006449.5	0.135439	0.464262	1.777289	3.61E-19	5.29E-18
DARS-AS1	0.097103	0.418528	2.107741	1.71E-30	2.60E-28
AC021086.1	0.02888	0.063123	1.128102	0.000743	0.00122
AC009268.2	0.149919	0.322386	1.104612	0.000879	0.001429
AC027228.2	0.05183	0.830841	4.002704	4.64E-26	2.06E-24
AL158151.2	0.039751	0.097261	1.290883	0.00193	0.002979
AC132192.1	0.076806	0.377963	2.298951	1.79E-12	1.01E-11
AL844908.1	0.348856	2.983842	3.096467	4.48E-13	2.70E-12
AC009806.1	0.759002	0.163621	-2.21375	1.27E-25	5.29E-24
AC020663.2	0.110042	0.690848	2.650313	1.67E-22	3.67E-21
TBL1XR1-AS1	0.014475	0.360497	4.638378	9.74E-08	2.83E-07
LINC01134	0.080749	0.246862	1.612192	8.28E-13	4.84E-12
AC012645.1	0.585365	1.816939	1.634102	0.00033	0.000569

AJ011932.1	0.401313	0.151752	-1.40302	5.90E-18	7.29E-17
AL450306.1	0.066734	0.169095	1.341342	2.30E-08	7.31E-08
AC103739.1	0.192666	0.450556	1.225604	0.002418	0.003671
ITFG1-AS1	0.155502	0.338257	1.121184	2.87E-06	6.70E-06
AC092338.2	0.020335	0.072947	1.842899	4.60E-05	9.03E-05
Z82188.2	0.0398	0.154252	1.954435	2.10E-11	1.02E-10
AP001189.1	2.288457	0.289995	-2.98027	9.50E-26	4.05E-24
LHFPL3-AS2	10.95965	2.941762	-1.89745	8.17E-25	2.77E-23
LINC01615	0.073887	0.400679	2.439063	8.44E-16	7.54E-15
LINC01338	0.030448	0.101093	1.731287	0.028429	0.036465
LINC01281	0.042504	0.176974	2.057873	3.64E-14	2.58E-13
AC008105.3	0.622611	1.537614	1.30429	2.00E-07	5.53E-07
MANCR	0.028027	0.241294	3.105892	5.88E-06	1.31E-05
AC009102.2	0.012559	0.029611	1.237388	0.005502	0.00789
AC007906.1	0.04803	0.258982	2.430844	0.004049	0.005934
LINC01169	0.660719	0.156967	-2.07358	5.12E-21	9.18E-20
AL158839.1	0.056692	0.253147	2.158753	6.68E-11	3.01E-10
AC093510.2	0.349327	0.117365	-1.57358	3.23E-22	6.87E-21
AC138932.5	0.22016	0.690505	1.649102	2.20E-07	6.04E-07
AC020656.2	0.041832	0.12605	1.591328	0.011604	0.015796
AC025822.2	0.041739	0.019805	-1.0755	6.00E-11	2.72E-10
AC124276.2	0.072129	0.177131	1.296155	0.022255	0.029018
AC148476.1	0.098368	0.386148	1.972889	2.15E-06	5.12E-06
AGAP11	0.13741	0.035991	-1.93278	1.04E-15	9.13E-15
AC004870.2	0.011986	0.34248	4.836635	1.76E-13	1.12E-12
AL109923.1	0.025163	0.181862	2.85349	0.004853	0.007015
LINC01571	0.612155	0.152134	-2.00855	1.03E-11	5.25E-11
AC091563.1	2.104304	4.686152	1.155061	2.00E-09	7.21E-09
AC008764.8	0.153885	0.39687	1.366814	6.89E-13	4.06E-12
CEP83-DT	0.100872	0.224156	1.151971	1.04E-10	4.57E-10
AC008937.1	0.014566	0.04192	1.525076	6.48E-06	1.44E-05
AC023154.1	0.007649	0.058314	2.930565	0.00117	0.001869
AP001094.1	0.399734	0.153877	-1.37726	3.56E-15	2.93E-14
AC090673.1	0.012572	0.187732	3.900387	4.81E-18	6.00E-17
Z83851.2	0.297673	0.833306	1.485119	1.11E-18	1.52E-17
AL136985.2	0.120082	0.246066	1.035021	0.000477	0.000806
AC243960.3	0.834174	0.307186	-1.44123	1.39E-17	1.63E-16
DNAH17-AS1	0.046206	0.153187	1.729126	1.96E-06	4.72E-06
AL117379.1	0.394227	1.282527	1.701891	4.55E-16	4.18E-15
AL133284.1	0.003124	0.132726	5.408693	1.13E-09	4.26E-09
LINC02244	0.730295	0.314623	-1.21485	2.23E-06	5.28E-06
LINC02099	0.013643	0.048266	1.822829	2.25E-17	2.54E-16
LINC00158	0.016336	0.047029	1.525463	5.79E-05	0.000112
LINC02332	0.019514	0.387722	4.312464	1.38E-23	3.65E-22

AL035461.2	0.80014	1.741213	1.121769	7.96E-10	3.06E-09
AC011632.1	0.004255	0.175379	5.365279	0.000595	0.000994
AC003965.2	0.260302	1.192344	2.195543	1.05E-11	5.34E-11
PACERR	0.155157	0.570389	1.878215	1.07E-06	2.67E-06
EIPR1-IT1	0.089108	0.323538	1.860305	5.08E-08	1.54E-07
AL449423.1	0.032678	0.1077	1.720611	0.003558	0.005267
AC115284.1	0.006264	0.031007	2.307421	5.17E-07	1.35E-06
AC012485.2	0.100648	0.311239	1.628705	0.028387	0.036418
LINC01150	1.4661	0.59146	-1.30963	6.54E-17	6.95E-16
AC046195.1	1.188577	0.246354	-2.27043	2.75E-26	1.29E-24
TMEM51-AS1	0.106742	0.403755	1.919356	2.29E-18	2.97E-17
PKNOX2-AS1	0.055336	0.0146	-1.92224	1.78E-09	6.50E-09
LINC01843	0.233793	1.123967	2.265298	1.34E-09	4.95E-09
AP000851.2	0.026437	0.204819	2.953692	0.024269	0.031453
SPAG5-AS1	0.22555	0.469319	1.057124	2.14E-10	8.90E-10
LINC00881	0.157566	0.339107	1.10578	0.001451	0.002279
AL583805.2	0.057903	0.021137	-1.45388	3.64E-13	2.21E-12
AC022028.2	0.007144	0.051245	2.842538	3.63E-07	9.67E-07
AC114296.1	0.02598	0.269971	3.377342	1.03E-07	2.97E-07
AC093591.2	0.011309	0.049235	2.122247	0.001101	0.001766
AC125603.4	0.005032	0.024225	2.267184	6.15E-06	1.37E-05
AL354726.1	0.060294	0.281277	2.221906	0.001288	0.002043
LINC02477	0.003536	0.048124	3.76666	4.40E-07	1.16E-06
AP000265.1	0.010327	0.024179	1.227283	0.012583	0.017036
AC010615.2	0.203943	0.712223	1.804162	4.71E-05	9.23E-05
LINC02422	0.099451	0.29603	1.573689	6.41E-05	0.000123
AL031665.1	0.145929	0.048067	-1.60214	5.32E-13	3.17E-12
AC021192.1	0.063701	0.024028	-1.4066	1.36E-08	4.44E-08
Z83851.1	0.826695	2.409685	1.543418	3.09E-28	2.31E-26
AC009005.1	0.657538	3.128304	2.250235	5.03E-22	1.02E-20
AP003119.3	0.196735	0.776718	1.981136	2.06E-11	1.00E-10
LINC02562	0.675246	3.376489	2.322039	5.99E-12	3.14E-11
AP001626.1	0.015739	0.121801	2.952153	3.08E-12	1.67E-11
AL590683.1	0.026467	0.012527	-1.07918	0.000262	0.000458
AL355802.2	0.247788	0.631562	1.349819	3.74E-12	2.02E-11
AL391069.2	0.380334	0.992862	1.384327	1.43E-18	1.91E-17
AP003031.1	0.009042	0.074121	3.035179	3.57E-10	1.43E-09
AP005136.2	0.201743	0.419535	1.056277	1.53E-06	3.72E-06
TDRKH-AS1	0.244094	1.070763	2.133131	4.14E-26	1.91E-24
ERVK-28	0.004028	0.095597	4.568777	1.62E-10	6.89E-10
AC008687.2	0.0246	0.10826	2.137793	1.73E-05	3.59E-05
DDX39B-AS1	0.043066	0.11288	1.390184	3.01E-07	8.09E-07
LINC02158	0.386453	0.161696	-1.25701	1.29E-23	3.42E-22
AL008723.1	0.132848	0.467925	1.816499	6.80E-06	1.50E-05

AC005522.1	0.040467	0.305491	2.916297	0.000282	0.000491
AC007285.2	0.056101	0.478144	3.091358	4.64E-22	9.49E-21
LINC02320	0.114582	0.425099	1.891414	8.05E-09	2.70E-08
AC010422.4	0.11723	0.344292	1.554293	0.000208	0.000369
IER3-AS1	0.331856	0.726626	1.130654	1.07E-10	4.67E-10
SNHG1	3.418572	11.15734	1.706527	7.65E-25	2.63E-23
AL359764.1	0.009125	0.25887	4.826251	7.50E-09	2.53E-08
LINC00501	0.005601	0.323507	5.852079	8.48E-12	4.37E-11
AC104237.1	0.069502	0.025986	-1.4193	6.10E-11	2.76E-10
AL731569.1	0.116378	0.270782	1.218307	1.79E-12	1.01E-11
AC010168.1	0.067428	0.37047	2.45793	4.47E-10	1.77E-09
HAR1B	0.084686	0.445339	2.394706	0.000683	0.001127
LYRM4-AS1	0.396955	0.906636	1.191549	4.85E-17	5.25E-16
AC127164.1	0.022684	0.16461	2.859278	8.20E-10	3.14E-09
Z83844.2	0.030525	0.079028	1.372365	2.44E-07	6.67E-07
AC104462.1	0.065985	0.23014	1.802301	1.23E-06	3.03E-06
AP003548.1	0.126936	0.036514	-1.79757	1.15E-18	1.57E-17
AC090589.2	0.040701	0.205634	2.336935	0.003735	0.005501
AP001025.1	0.019558	0.149084	2.93027	0.014049	0.018897
LINC01393	0.044402	0.201812	2.184322	5.87E-12	3.09E-11
AL139039.3	0.023476	0.091774	1.966914	7.46E-08	2.21E-07
AC005291.2	0.032974	0.334307	3.341758	4.85E-09	1.67E-08
AC016394.1	0.544067	1.448017	1.412222	1.10E-14	8.35E-14
AL365277.1	0.194888	0.800261	2.037828	1.95E-05	4.03E-05
AC018467.1	0.063776	0.018061	-1.82011	1.92E-10	8.03E-10
AC114811.2	0.038011	0.13469	1.825158	1.74E-12	9.81E-12
AC008278.2	0.258646	0.083067	-1.63864	4.63E-07	1.22E-06
LINC01429	0.002234	0.024413	3.450025	9.43E-08	2.75E-07
AL359237.1	0.01519	0.005952	-1.35177	5.14E-08	1.55E-07
AL512306.2	0.15711	0.31888	1.021243	0.002702	0.004073
AC099687.1	0.054824	0.021446	-1.35408	2.55E-13	1.58E-12
SMCR2	0.021671	0.091298	2.074841	7.20E-10	2.78E-09
AC097359.3	0.03347	0.013073	-1.35634	2.85E-06	6.65E-06
AL445222.1	0.313847	0.734481	1.226666	1.26E-10	5.46E-10
LINC01792	0.00247	0.04191	4.084913	3.11E-11	1.48E-10
LINC01117	0.087932	0.317705	1.85323	0.000372	0.000637
LINC00330	0.009708	0.053877	2.472488	2.55E-06	6.00E-06
AC092802.2	0.123132	0.26186	1.088587	0.001404	0.002214
AC007391.1	0.026356	0.061202	1.21543	0.002541	0.003843
CPEB1-AS1	0.026956	0.011816	-1.18985	2.27E-05	4.65E-05
AL353807.2	0.058628	0.322544	2.459833	2.96E-14	2.13E-13
PLCH1-AS2	0.030295	0.13745	2.181764	0.000243	0.000428
AC027307.1	0.080454	0.211058	1.391409	4.54E-06	1.03E-05
AP003119.1	0.054667	0.708072	3.695154	1.69E-05	3.51E-05

LINC02570	0.588438	0.058436	-3.33195	1.31E-31	2.80E-29
AC079062.1	0.011348	1.298726	6.838565	4.12E-14	2.89E-13
AC020911.2	0.136019	0.051662	-1.39664	3.43E-08	1.07E-07
AP006621.4	0.255547	0.681251	1.414599	2.45E-05	4.98E-05
AC104365.2	0.08824	0.033711	-1.38819	1.92E-05	3.97E-05
AC010632.1	0.054061	0.170618	1.658115	5.48E-07	1.42E-06
AL139385.1	0.060049	0.236342	1.976659	1.61E-08	5.23E-08
AC008669.1	1.760018	0.718384	-1.29276	1.47E-24	4.78E-23
AC025280.2	0.073092	0.028668	-1.35025	8.09E-08	2.38E-07
AC090164.3	0.00297	0.053677	4.175743	1.49E-11	7.40E-11
AC099850.3	1.172631	6.783185	2.532214	7.83E-23	1.84E-21
LINC01301	0.085888	0.219301	1.352389	1.59E-09	5.83E-09
AC017100.1	0.24935	0.638021	1.35543	3.87E-05	7.64E-05
AC018529.1	1.086103	0.220733	-2.29879	6.22E-23	1.48E-21
AL442224.1	0.071844	1.069923	3.896493	0.001863	0.002886
CHKB-DT	0.594305	1.385503	1.221134	6.28E-14	4.27E-13
AC098828.2	0.013992	0.047133	1.752102	8.29E-05	0.000157
AL158166.1	0.829785	1.966222	1.244616	7.28E-05	0.000139
LINC02544	0.086892	1.159096	3.737636	1.55E-20	2.58E-19
AL391335.1	0.034617	0.076095	1.136336	0.021358	0.027937
LINC02257	0.57298	3.022028	2.39896	1.19E-06	2.94E-06
AP001893.3	0.019814	0.084778	2.097201	0.000118	0.000218
AC132872.2	0.05252	0.144411	1.459245	1.90E-09	6.88E-09
ZNF451-AS1	0.099322	0.234284	1.238072	0.01025	0.014077
AC022167.4	0.018065	0.105956	2.55217	3.19E-11	1.52E-10
AC087645.2	0.268766	0.591717	1.13856	1.10E-11	5.58E-11
AL158068.2	0.020947	0.060569	1.531843	0.004994	0.007208
AC100860.1	0.079323	0.205238	1.371489	0.020876	0.027369
AC024941.2	0.356843	0.756678	1.084389	4.35E-08	1.33E-07
AC097358.2	0.012078	0.115288	3.254811	2.51E-20	4.11E-19
SNHG4	0.288969	1.537934	2.412007	1.54E-24	4.96E-23
AC008840.1	0.091712	0.221228	1.270354	1.62E-07	4.53E-07
ZNF252P-AS1	0.03817	0.169936	2.154496	4.11E-18	5.19E-17
FARSA-AS1	0.045593	0.135417	1.570531	0.000493	0.000833
AC092723.1	0.087885	0.032532	-1.43376	1.54E-09	5.68E-09
AC007608.1	0.135015	0.064338	-1.06937	2.69E-17	3.01E-16
LINC02512	0.35324	0.105319	-1.74589	3.41E-14	2.43E-13
AC092535.3	0.068672	0.449379	2.710146	0.005034	0.007263
KRTAP5-AS1	0.287086	0.580933	1.016888	0.003199	0.004764
LINC01975	0.061535	0.023175	-1.40882	8.08E-10	3.10E-09
AC010776.2	1.131822	0.176526	-2.6807	5.18E-28	3.58E-26
AC027348.1	0.208874	0.097869	-1.09372	1.97E-09	7.12E-09
AC005383.1	0.141281	0.289307	1.034036	0.026651	0.034304
AC233976.1	0.208415	0.098839	-1.07631	7.05E-11	3.16E-10

GTF3C2-AS1	0.056424	0.168303	1.576688	2.38E-11	1.16E-10
AL008726.1	0.103639	0.295854	1.513312	5.27E-11	2.41E-10
AC010285.1	0.025995	0.140905	2.438417	3.35E-08	1.04E-07
AC124067.2	0.298331	0.859826	1.527129	0.014264	0.01917
AL355338.1	1.833177	6.544702	1.835981	3.23E-24	9.94E-23
AC096677.1	0.994871	2.443515	1.296376	2.44E-19	3.62E-18
AP006621.1	0.07382	0.177597	1.266533	0.001876	0.002905
TCL6	0.013705	0.050347	1.87721	0.00951	0.013139
AC011442.1	0.240856	0.640609	1.411271	2.16E-06	5.15E-06
MED4-AS1	1.219727	0.292358	-2.06075	1.50E-24	4.86E-23
AC007848.1	0.01764	0.246199	3.802925	0.00018	0.000322
AC124016.1	0.196955	0.432952	1.136339	2.43E-11	1.18E-10
MIR9-3HG	0.090452	0.644579	2.833128	1.90E-06	4.60E-06
AL031768.1	0.129906	0.504843	1.958368	1.45E-14	1.08E-13
AL359881.1	0.332785	1.17576	1.820932	0.000225	0.000398
LINC00862	0.028148	0.474938	4.076648	4.57E-22	9.37E-21
HID1-AS1	1.292926	0.194792	-2.73063	5.23E-30	6.02E-28
DKFZP434A062	0.020538	0.009906	-1.05197	3.94E-07	1.04E-06
AC099066.1	0.021356	0.056191	1.395722	0.010779	0.014748
AC026362.1	0.047324	0.121686	1.362522	4.27E-14	2.99E-13
LINC01625	0.111974	0.037746	-1.56875	1.49E-21	2.88E-20
AL356966.1	0.027924	0.061607	1.141594	0.000304	0.000527
AC087294.1	0.086771	0.333235	1.941252	2.01E-10	8.38E-10
AC018358.1	0.029446	0.003793	-2.95655	1.23E-30	1.92E-28
AL670729.3	0.031502	0.083417	1.404883	4.48E-05	8.80E-05
AC010980.2	0.240384	0.705358	1.553016	7.32E-06	1.61E-05
LINC01415	0.241904	0.092898	-1.38072	8.00E-21	1.39E-19
AC084083.1	0.041854	0.236165	2.496354	5.34E-09	1.83E-08
AC138305.1	0.011168	0.155518	3.799651	3.35E-06	7.72E-06
AC061975.7	0.034045	0.116916	1.779963	6.45E-06	1.43E-05
AC073320.1	0.014528	0.04799	1.723923	2.05E-08	6.55E-08
AL162431.1	0.032812	0.25628	2.965415	5.55E-13	3.29E-12
HDAC11-AS1	0.027949	0.089043	1.67173	3.76E-07	9.99E-07
AC067930.3	0.097585	0.237811	1.285076	1.99E-05	4.10E-05
AC135782.3	0.033699	0.102645	1.606886	2.20E-07	6.06E-07
AC012640.1	0.015161	0.108895	2.844544	8.07E-15	6.24E-14
AC137834.2	0.009493	0.068383	2.848709	1.07E-09	4.01E-09
AC243772.2	0.04463	0.133832	1.584348	1.04E-06	2.60E-06
AC011825.2	0.175462	0.526727	1.585895	6.87E-05	0.000131
AC020928.1	0.035232	0.310912	3.141564	1.63E-08	5.26E-08
AC027796.5	0.064243	0.202695	1.657691	1.23E-05	2.64E-05
AC144831.1	1.794141	0.580141	-1.62882	1.81E-22	3.97E-21
AC021028.1	0.249444	0.564862	1.17918	4.84E-07	1.27E-06
AL121658.1	0.110478	0.291878	1.401609	5.23E-11	2.40E-10

AC090502.1	0.00319	0.508427	7.316236	7.02E-22	1.39E-20
AC093159.1	0.557924	0.192435	-1.5357	5.97E-08	1.79E-07
AC024451.4	0.128776	0.460995	1.839882	5.56E-08	1.67E-07
AL450384.1	0.115847	0.320112	1.466354	0.004157	0.006078
LUARIS	0.006452	0.037297	2.531293	3.00E-10	1.22E-09
LINC00589	0.045648	0.207951	2.187621	5.10E-07	1.33E-06
AP000346.1	0.056644	0.118192	1.06114	0.010256	0.014081
AC105429.1	0.053812	0.193924	1.849481	4.45E-08	1.36E-07
AC092755.1	0.14368	0.451521	1.651928	0.00026	0.000456
AC093249.2	0.088265	0.405211	2.198758	3.49E-18	4.43E-17
AJ239328.1	0.015008	0.050128	1.739862	4.79E-05	9.36E-05
AL121820.2	0.491001	1.514023	1.624588	3.05E-08	9.56E-08
LINC01945	0.053061	0.005004	-3.40654	3.20E-30	4.23E-28
AC005329.3	0.027981	0.060803	1.119694	0.004781	0.006921
AL022157.1	0.184878	0.401145	1.117551	1.97E-09	7.14E-09
AC011899.2	2.775815	0.598294	-2.21399	1.17E-30	1.86E-28
AC004908.2	0.374647	0.913851	1.286428	1.44E-10	6.14E-10
TRPM2-AS	0.048435	0.600265	3.631475	5.73E-07	1.48E-06
AC007996.1	0.565952	1.470034	1.377098	4.84E-11	2.23E-10
AC010273.2	0.686513	1.397331	1.025315	0.000542	0.00091
AC138207.7	0.058239	0.141397	1.279689	0.000515	0.000868
AC073111.1	0.065461	0.207277	1.662853	7.91E-07	2.01E-06
LINC00636	0.087222	0.033448	-1.38275	6.64E-08	1.98E-07
AC026368.1	0.151964	0.983927	2.694824	5.56E-11	2.53E-10
AC073316.2	0.041812	0.705878	4.077426	2.07E-09	7.43E-09
KDM4A-AS1	0.250386	0.643144	1.360988	9.09E-15	6.98E-14
AC025040.1	0.010088	0.070332	2.801604	0.008158	0.011387
AL445224.1	0.050285	0.010509	-2.25852	7.46E-14	5.01E-13
AL512631.1	0.059588	0.125938	1.079608	0.003797	0.005585
LINC02331	0.041607	0.320305	2.944545	1.10E-06	2.74E-06
NKILA	0.700896	1.873408	1.418392	1.54E-10	6.57E-10
LINC01082	2.056705	0.115522	-4.15409	1.11E-32	3.58E-30
GAS5	26.33212	62.40668	1.244877	4.34E-13	2.62E-12
AC004888.1	0.013963	0.140431	3.330191	2.51E-06	5.90E-06
AL445686.2	0.084901	0.182347	1.102836	0.000181	0.000324
AL355601.1	0.856559	0.407114	-1.07312	0.001878	0.002906
OVOL1-AS1	0.04408	0.107165	1.281647	0.004247	0.006196
LL22NC03-63E9.3	0.00168	0.027395	4.027137	6.02E-10	2.35E-09
FAM83A-AS1	0.032945	3.364168	6.674065	3.95E-31	7.22E-29
AC022126.1	0.086984	0.363476	2.063038	2.18E-09	7.81E-09
LINC01197	0.322875	0.087388	-1.88547	1.67E-28	1.39E-26
AC007038.2	0.754055	1.526101	1.01711	1.14E-06	2.82E-06
LINC01747	0.077647	0.032376	-1.262	2.80E-12	1.53E-11
AC007128.2	0.002188	0.339649	7.278064	2.02E-17	2.30E-16

LINC00467	1.528915	3.413781	1.158862	6.19E-20	9.87E-19
AL122125.1	0.095044	0.382423	2.008507	7.28E-11	3.25E-10
LINC02015	0.0537	0.369667	2.783236	5.05E-09	1.73E-08
AC006116.9	0.008916	0.034988	1.972315	0.000274	0.000477
AC061975.6	0.002388	0.406332	7.410907	4.95E-13	2.97E-12
AL049869.3	0.323688	0.850912	1.394407	0.008712	0.012103
AP001033.2	0.507708	1.337117	1.397055	2.12E-09	7.61E-09
LINC01814	0.056815	0.160261	1.496092	2.57E-09	9.14E-09
AC113189.4	0.001502	0.007073	2.235818	6.84E-09	2.32E-08
AC092691.1	1.206331	0.168921	-2.83621	3.78E-30	4.52E-28
AC004837.2	0.160725	0.518635	1.690126	2.02E-08	6.47E-08
LINC01587	0.028433	0.268416	3.238826	2.94E-09	1.03E-08
LMO7DN	0.375926	0.15274	-1.29937	1.26E-12	7.24E-12
LINC02004	0.359869	0.732301	1.024965	0.027023	0.034754
AC244517.2	0.02619	0.064257	1.294841	0.016164	0.021491
FAM138B	0.031544	0.012608	-1.32304	2.28E-08	7.26E-08
LINC01691	0.029722	0.11533	1.956182	1.64E-05	3.42E-05
AC018816.1	0.370296	1.15691	1.643525	1.97E-12	1.10E-11
LINC02469	0.048829	0.023069	-1.0818	2.48E-08	7.83E-08
AC099684.2	0.061087	0.142767	1.224725	0.003265	0.004857
SZT2-AS1	0.032906	0.072679	1.143198	0.000238	0.000419
AC093278.2	4.334245	1.256125	-1.7868	2.57E-27	1.50E-25
AC096734.1	0.011455	0.066281	2.532552	2.95E-09	1.03E-08
AC013652.1	0.059689	0.389094	2.704571	1.33E-10	5.73E-10
HLCS-IT1	0.043746	0.183229	2.066422	0.001577	0.002462
AC005180.2	0.68975	0.200287	-1.784	1.35E-18	1.82E-17
AC134775.1	0.090705	0.235931	1.379103	0.001136	0.001815
AC007405.2	0.007251	0.080534	3.473292	2.87E-09	1.01E-08
AC233280.1	0.017811	0.108263	2.603707	3.84E-18	4.87E-17
LINC02585	0.383732	0.991008	1.3688	9.00E-14	5.95E-13
AC092718.4	2.343545	5.099391	1.121633	6.84E-15	5.41E-14
AC084880.3	0.378482	0.091215	-2.05289	4.45E-18	5.58E-17
AC009159.2	0.034573	0.109191	1.659122	0.006385	0.009075
AC011933.3	0.091163	0.258659	1.504536	0.001885	0.002916
AC040934.1	0.079715	0.243008	1.60808	1.42E-05	3.00E-05
C15orf54	0.021713	0.176967	3.026836	2.72E-06	6.37E-06
AC245060.2	0.223618	0.560371	1.325349	3.62E-09	1.26E-08
GPC5-AS1	0.592138	0.173908	-1.7676	4.73E-13	2.84E-12
LINC01355	0.406109	1.260899	1.634514	8.67E-13	5.07E-12
AC073352.1	0.069985	0.239544	1.775172	1.15E-09	4.30E-09
AL008628.1	0.030775	0.009134	-1.7524	2.91E-17	3.24E-16
AC008760.2	0.622172	1.927225	1.631139	0.026612	0.034261
LINC02299	0.109818	0.017821	-2.62347	3.18E-15	2.65E-14
LINC02147	0.124196	0.043205	-1.52334	6.63E-17	7.04E-16

AC008434.1	0.055758	0.187925	1.752908	1.70E-09	6.23E-09
AC018648.1	0.108205	0.300284	1.472569	8.67E-07	2.19E-06
PURPL	0.014019	0.229999	4.036219	0.001426	0.002244
UFL1-AS1	0.038938	0.101541	1.382791	0.007931	0.011098
AC011815.1	0.259073	0.603559	1.220136	6.31E-11	2.85E-10
AC138904.1	0.036278	0.376226	3.374435	3.48E-22	7.35E-21
AP001189.3	3.579495	0.678061	-2.40027	1.43E-27	8.77E-26
AL136982.6	0.073202	0.025584	-1.51663	8.68E-08	2.55E-07
ERI3-IT1	0.072351	0.321669	2.1525	0.022984	0.0299
AP003064.2	1.323382	0.265458	-2.31767	3.25E-16	3.09E-15
AC104170.1	0.139731	0.36621	1.390017	4.66E-06	1.05E-05
AL033527.2	0.01358	0.062089	2.19282	2.74E-07	7.43E-07
AL359378.1	0.211465	0.02664	-2.98875	1.79E-34	1.01E-31
AC124319.1	0.422717	1.713232	2.018957	1.13E-08	3.75E-08
AL589765.4	0.790003	2.55358	1.692592	4.48E-21	8.09E-20
AL137802.2	0.056872	0.132959	1.225179	0.000205	0.000365
AC005091.1	0.019737	0.061263	1.634126	2.45E-07	6.69E-07
AC022165.1	0.023093	0.148229	2.682305	2.49E-10	1.03E-09
AC008105.1	0.224156	0.469087	1.065354	4.99E-06	1.12E-05
LINC01890	0.018669	0.007789	-1.26119	2.91E-06	6.78E-06
KCNMB2-AS1	0.026735	1.39743	5.7079	1.07E-13	7.00E-13
AC004988.1	0.863236	0.314966	-1.45456	7.20E-18	8.82E-17
MGC27382	1.304557	0.192201	-2.76287	2.04E-28	1.65E-26
AC116025.2	0.021662	0.243294	3.48943	3.80E-15	3.11E-14
VPS9D1-AS1	0.425291	3.235547	2.927486	4.44E-25	1.60E-23
ADAMTS9-AS2	0.425882	0.187319	-1.18496	2.73E-23	6.77E-22
SCAANT1	0.236698	0.567965	1.262756	5.54E-06	1.24E-05
AC078909.2	0.054897	0.218609	1.993546	3.33E-10	1.34E-09
GACAT2	0.011804	0.069431	2.556312	0.002088	0.003202
AC090164.2	0.021396	0.138304	2.692448	0.000298	0.000517
AP002954.1	1.932686	0.849003	-1.18676	1.93E-15	1.65E-14
DPP10-AS1	0.155096	1.833972	3.563734	0.000256	0.000448
AC011352.3	0.002755	0.206967	6.231438	8.68E-17	9.03E-16
EWSAT1	0.028841	0.081365	1.496275	0.007994	0.011183
AC010719.1	0.215032	1.661123	2.949533	2.85E-19	4.21E-18
AP003555.3	0.513404	0.175245	-1.55072	3.07E-16	2.92E-15
LINC00092	0.922654	0.272426	-1.75993	2.41E-25	9.36E-24
IFNG-AS1	0.082003	0.3153	1.942984	1.86E-07	5.17E-07
AC019294.2	0.01664	0.046367	1.478421	7.17E-11	3.21E-10
AC018695.3	0.027506	0.080329	1.54618	0.002018	0.003104
AC008063.1	0.169227	0.910726	2.428058	7.19E-06	1.58E-05
LINC01926	0.009229	0.179557	4.282159	1.43E-14	1.07E-13
AL450332.1	0.054152	0.022365	-1.27578	1.49E-08	4.85E-08
RMDN2-AS1	0.02836	0.202342	2.834879	9.20E-16	8.16E-15

AC098936.1	0.006356	0.034645	2.446529	1.31E-09	4.85E-09
FP325332.1	0.035247	0.091441	1.375352	0.008987	0.012464
AP001363.2	0.549225	0.210165	-1.38588	3.28E-18	4.17E-17
AL035252.2	0.006949	0.087396	3.652637	2.36E-07	6.48E-07
AC103724.4	0.385543	0.178429	-1.11154	1.69E-11	8.38E-11
AC005237.1	0.118736	0.404074	1.766866	0.006585	0.009334
AC234781.1	0.010562	0.040593	1.942345	3.73E-07	9.91E-07
AC009065.1	0.010032	0.025541	1.348273	0.003612	0.005335
LOXL1-AS1	0.484037	1.285467	1.409102	2.58E-18	3.33E-17
LINC01268	0.64766	0.291446	-1.15201	4.72E-16	4.33E-15
AC015849.1	0.044934	0.113329	1.334622	0.000891	0.001446
AL360004.1	0.009659	0.028439	1.557975	0.000659	0.00109
AC010624.1	0.413426	0.121132	-1.77104	7.53E-11	3.36E-10
LINC01786	0.318161	0.875346	1.460098	3.05E-08	9.56E-08
HHATL-AS1	0.308511	0.076878	-2.00468	9.95E-13	5.75E-12
BASP1-AS1	0.014827	0.06053	2.029414	0.004268	0.006223
AC091588.2	0.07644	0.019869	-1.94379	2.86E-09	1.01E-08
AC063944.3	0.143361	0.063344	-1.17838	2.70E-07	7.32E-07
AC084864.1	0.032884	0.503739	3.937207	5.87E-25	2.05E-23
AC022007.1	0.631935	1.3026	1.043546	5.33E-14	3.68E-13
FAM167A-AS1	0.142745	0.049466	-1.52894	2.91E-22	6.23E-21
AC110769.2	0.049172	0.190303	1.952383	3.04E-12	1.65E-11
AC068987.1	0.13173	0.471021	1.838207	0.000446	0.000757
AC099791.2	0.055633	0.125243	1.170727	0.000338	0.000583
AL512283.1	0.006378	0.065638	3.363273	1.51E-05	3.16E-05
LIVAR	0.044239	0.196088	2.148096	0.002539	0.003842
MIR137HG	0.001356	0.076191	5.812376	2.07E-09	7.44E-09
AC019193.2	0.65211	0.134299	-2.27966	6.11E-24	1.74E-22
AC083809.1	0.164243	5.413098	5.042549	0.000439	0.000746
AC010186.1	0.007795	0.058532	2.908554	5.24E-07	1.36E-06
AC007277.1	0.247033	0.080411	-1.61924	7.55E-18	9.24E-17
UBE2Q1-AS1	0.158934	0.638783	2.006897	2.29E-15	1.94E-14
AL645924.1	1.981918	0.840393	-1.23776	0.000253	0.000443
AL391095.1	0.087046	0.200108	1.200933	2.91E-05	5.83E-05
AL360270.2	0.042685	0.135123	1.662471	8.01E-07	2.03E-06
AC105118.1	0.040444	1.049017	4.696961	9.95E-13	5.75E-12
AC079305.3	0.132257	0.063503	-1.05846	0.001625	0.002533
AC016396.1	0.04408	0.093953	1.0918	0.000825	0.001348
CSE1L-AS1	0.027352	0.094754	1.792545	1.48E-07	4.18E-07
AC008609.1	0.186203	0.064957	-1.51931	0.00012	0.000222
AC068228.1	0.00539	0.417197	6.274356	3.20E-27	1.82E-25
LINC01168	0.03365	0.006451	-2.38304	5.34E-14	3.68E-13
TBX2-AS1	3.403922	0.887698	-1.93906	4.29E-27	2.36E-25
AL356608.1	0.079743	0.037412	-1.09185	3.22E-06	7.44E-06

AC005726.2	0.023367	0.139926	2.5821	1.04E-10	4.57E-10
AC103702.2	0.038317	0.888387	4.535149	3.21E-09	1.12E-08
DANCR	5.953401	12.08299	1.021191	4.93E-15	3.98E-14
AC005180.1	0.412432	0.141324	-1.54515	8.29E-17	8.67E-16
AC108751.4	0.056245	0.210807	1.906139	4.01E-08	1.23E-07
LINC02195	0.34065	1.353275	1.990094	8.98E-09	2.99E-08
LINC01979	0.010346	0.061738	2.577124	1.54E-13	9.87E-13
AC026412.3	0.191094	0.635627	1.733896	4.34E-12	2.33E-11
U62317.1	0.870825	6.917477	2.989792	8.00E-27	4.11E-25
AL356055.1	0.041374	0.406469	3.296355	2.26E-05	4.62E-05
AC019193.3	0.087658	0.032456	-1.43337	8.81E-10	3.35E-09
LINC01204	0.003446	0.021365	2.632087	0.002582	0.003901
LINC01876	0.143151	0.815821	2.510715	7.49E-08	2.22E-07
AC245884.10	0.065944	0.250315	1.92444	0.000157	0.000283
AC012447.1	0.078948	0.269518	1.771402	4.43E-07	1.17E-06
AC025580.2	0.174431	0.498552	1.515085	1.71E-12	9.65E-12
AL035530.1	0.63499	0.262699	-1.27332	3.03E-11	1.45E-10
AC013480.1	0.150918	0.046728	-1.69142	1.67E-13	1.06E-12
MIR194-2HG	0.011577	0.062547	2.43371	0.000522	0.000878
AC026495.1	0.020677	0.055698	1.429608	0.00404	0.005922
AC011676.2	0.025043	0.110978	2.147814	0.009067	0.012569
AC073323.1	0.013639	0.133322	3.28915	2.55E-06	5.99E-06
LINC00924	0.368154	0.164544	-1.16184	2.90E-15	2.42E-14
LINC00028	0.107843	0.044003	-1.29327	1.07E-07	3.08E-07
AL451070.1	0.043403	0.165665	1.932395	0.009932	0.013673
AC084855.1	0.119109	0.049589	-1.26419	3.21E-11	1.52E-10
AL731563.3	0.143733	0.310903	1.113077	3.53E-10	1.42E-09
AL021707.2	0.228376	0.694612	1.604798	1.70E-16	1.68E-15
AC002059.1	0.18656	0.426303	1.19224	5.88E-05	0.000114
AC092720.1	0.057384	0.129979	1.179554	1.97E-07	5.43E-07
LINC02185	1.651269	0.490157	-1.75226	6.65E-23	1.58E-21
LINC02343	0.00767	0.075261	3.294666	9.14E-08	2.67E-07
AC069277.1	0.004102	0.110482	4.751214	1.23E-06	3.03E-06
AL137060.1	0.04698	0.149684	1.671796	2.37E-16	2.29E-15
WASIR2	0.042712	0.577077	3.756043	4.07E-24	1.23E-22
AL359504.2	0.091307	0.32711	1.840978	4.81E-09	1.66E-08
AC010789.1	0.011539	0.906158	6.295227	1.85E-10	7.76E-10
DSCR9	0.032875	0.089126	1.438836	1.04E-10	4.56E-10
ZFPM2-AS1	0.042389	1.88721	5.476436	5.17E-23	1.24E-21
AC106037.2	0.106678	0.271539	1.347892	0.007131	0.010056
AC107926.1	0.021242	0.100317	2.239567	1.06E-05	2.29E-05
AC093895.1	0.029298	0.403016	3.781945	3.92E-17	4.31E-16
AC130371.1	0.028483	0.159102	2.481792	0.000229	0.000405
CADM3-AS1	0.42499	0.108248	-1.97309	1.18E-19	1.80E-18

AC090260.1	0.028609	0.075493	1.399856	3.91E-08	1.20E-07
AL031667.3	0.169414	0.556641	1.716194	1.05E-10	4.60E-10
LINC02243	0.045774	0.020122	-1.18578	4.71E-11	2.18E-10
AC087276.2	0.095757	0.231758	1.275174	0.001541	0.002409
AC090527.3	0.076601	0.199497	1.380939	6.53E-07	1.67E-06
AC078778.2	0.092319	0.32702	1.824682	1.02E-06	2.55E-06
AC025265.1	0.097658	0.351926	1.849468	4.30E-14	3.00E-13
AL392046.1	0.016486	0.053072	1.686667	5.89E-05	0.000114
BX324167.1	0.023627	0.119213	2.335017	0.009177	0.01271
OGFRP1	0.154692	0.500079	1.692762	4.70E-25	1.68E-23
AL161756.1	0.029938	0.093396	1.641356	8.55E-14	5.67E-13
LINC01800	0.023799	0.049013	1.042244	4.05E-05	7.98E-05
AL022069.1	0.035563	0.085628	1.26771	8.32E-07	2.11E-06
ZBTB20-AS1	0.022879	0.259769	3.505118	3.97E-06	9.07E-06
AL353152.1	0.446821	0.11303	-1.98299	1.22E-24	4.05E-23
AC084026.2	0.035539	0.254852	2.842205	8.62E-07	2.18E-06
AC006042.1	1.399674	4.051588	1.533396	1.01E-11	5.17E-11
CBR3-AS1	0.446056	0.915969	1.038073	4.90E-09	1.69E-08
AC009185.1	0.021928	0.044035	1.005873	0.003045	0.004553
LINC01771	0.044614	0.169103	1.922342	4.36E-10	1.73E-09
AL645608.6	0.028067	0.378149	3.752022	3.62E-15	2.98E-14
AC008243.1	0.049488	0.23736	2.261917	0.006855	0.009681
AC006946.2	0.037072	0.271548	2.872819	1.19E-07	3.40E-07
LINC00517	0.004404	0.032163	2.868595	0.025551	0.03299
LINC02433	0.005057	0.086602	4.097916	1.60E-05	3.35E-05
AC090517.2	0.339611	0.749588	1.142215	6.65E-08	1.98E-07
AC010491.1	0.054295	0.192005	1.82226	3.58E-14	2.54E-13
LINC00632	0.044184	0.130244	1.559625	0.001503	0.002353
Z84485.1	0.133058	0.696749	2.388585	1.33E-21	2.58E-20
AL592114.3	0.283103	0.13404	-1.07866	1.63E-10	6.90E-10
AL022318.1	0.022707	0.115441	2.345935	0.004644	0.006738
UBXN10-AS1	0.544905	2.024848	1.893738	1.74E-10	7.34E-10
LINC00160	0.033002	0.544028	4.043064	5.50E-18	6.83E-17
AC145207.8	0.30177	0.726947	1.2684	0.000857	0.001396
AC012594.1	0.000943	0.012365	3.713604	2.45E-06	5.75E-06
AC055855.2	0.015748	0.065199	2.049648	5.48E-07	1.42E-06
AL353764.1	0.158473	0.325594	1.03884	0.000195	0.000348
LINC01123	0.293797	0.680611	1.212011	4.64E-08	1.42E-07
AC079907.1	0.246534	0.537927	1.125623	3.37E-09	1.18E-08
AC245041.1	3.895285	0.911985	-2.09465	3.49E-22	7.36E-21
AC092436.2	0.016237	0.71877	5.468144	6.76E-10	2.63E-09
AC084125.2	0.114173	0.458307	2.005096	8.90E-15	6.84E-14
LNCSRLR	0.072906	0.41192	2.498246	3.59E-06	8.25E-06
C1orf147	0.048224	0.203163	2.074804	5.55E-16	5.06E-15

AC073534.1	0.249808	0.651823	1.383659	0.001536	0.002403
AC245052.4	0.109253	0.280481	1.360233	3.23E-15	2.67E-14
AC015914.1	0.804272	0.354465	-1.18204	4.20E-16	3.89E-15
AC004156.1	0.018134	0.062481	1.784687	2.86E-11	1.37E-10
THOC7-AS1	0.027511	0.168041	2.610741	0.004276	0.006233
AC104135.1	0.113158	0.242248	1.098143	0.033524	0.042516
FLJ34503	0.1882	0.042757	-2.13803	1.13E-18	1.53E-17
LINC00894	0.246068	0.555677	1.175188	5.48E-05	0.000106
AC007991.2	0.861625	2.590702	1.588211	0.030552	0.038994
AC004130.1	0.753565	1.802313	1.258045	9.61E-06	2.08E-05
AL365181.3	0.187848	4.208941	4.48582	2.32E-17	2.61E-16
AC118757.1	0.023927	0.051013	1.092209	0.011473	0.015632
AP001001.1	0.115973	0.318065	1.45553	1.82E-08	5.86E-08
LINC01556	0.1744	0.565293	1.696596	2.60E-06	6.11E-06
AC025171.5	0.286832	0.657026	1.195743	9.73E-08	2.83E-07
AC009229.2	0.076651	0.210655	1.458496	0.003677	0.005424
AC092675.1	0.033826	0.225576	2.737419	5.84E-07	1.51E-06
AC130324.2	0.024243	0.066335	1.45217	0.002203	0.003363
AC008554.1	0.018686	0.113205	2.598875	0.000369	0.000632
AC027514.1	0.013602	0.158399	3.541648	0.003989	0.00585
AL110115.1	0.065952	0.390908	2.567333	1.09E-06	2.72E-06
AL645608.7	0.361162	0.727503	1.010308	0.006794	0.0096
FSIP2-AS1	0.052296	0.151009	1.529853	0.000665	0.0011
LINC02596	0.081681	0.029429	-1.47278	1.73E-07	4.82E-07
AL513217.1	0.079389	0.248621	1.64693	0.000162	0.000291
AL645608.2	0.014135	0.17428	3.624038	1.94E-13	1.22E-12
AC005821.1	0.026762	0.067184	1.327951	0.007662	0.010746
AC005041.3	0.209647	0.892219	2.089437	4.91E-24	1.45E-22
LINC01480	0.34007	0.729479	1.101034	2.03E-06	4.86E-06
AC026355.1	0.370284	1.683838	2.185048	1.25E-06	3.08E-06
SMAD9-IT1	0.374372	0.178901	-1.06531	5.76E-12	3.03E-11
ST7-OT4	0.0278	0.308623	3.47271	8.43E-06	1.84E-05
AC134407.1	0.072835	0.246126	1.756686	0.000102	0.000191
AC104984.2	0.023327	0.198086	3.086053	1.95E-05	4.03E-05
AL049836.1	0.177689	0.786054	2.145271	3.09E-11	1.47E-10
AC104472.1	0.022659	0.13777	2.604138	1.43E-10	6.14E-10
AC124944.1	0.038427	0.094995	1.30571	6.44E-06	1.43E-05
AC087239.1	0.212063	0.521346	1.297747	8.09E-07	2.05E-06
AP001610.1	0.084587	0.230866	1.448551	0.000173	0.000311
AC105415.1	0.021239	0.008731	-1.28243	3.09E-06	7.16E-06
AC022240.1	0.008738	0.102868	3.557419	4.09E-08	1.26E-07
SSTR5-AS1	0.002405	0.117508	5.610864	4.44E-05	8.71E-05
AL512413.1	0.193362	0.89611	2.212375	2.06E-13	1.30E-12
AL355512.1	0.040843	0.211977	2.375761	2.07E-08	6.61E-08

AL662890.1	0.030412	0.243531	3.001389	2.40E-12	1.32E-11
AC067930.5	0.055796	0.286832	2.361972	1.91E-13	1.20E-12
AL354811.1	0.066799	0.173352	1.375805	0.000505	0.000851
AC007342.4	0.294305	1.116404	1.923477	6.88E-16	6.20E-15
AP001574.1	0.134932	1.157694	3.100953	4.54E-10	1.80E-09
U62317.4	0.366484	2.988135	3.027421	2.24E-22	4.86E-21
AC009962.1	0.296578	0.113659	-1.38369	2.01E-24	6.39E-23
AC120036.4	0.088212	0.489061	2.47096	5.11E-15	4.11E-14
AP005717.1	0.022094	0.084075	1.928025	1.20E-14	9.06E-14
AC010205.1	0.126436	0.041468	-1.60835	1.43E-13	9.22E-13
AC226101.1	0.160738	0.039559	-2.02263	1.74E-20	2.90E-19
AP001412.1	0.093986	0.216447	1.203487	1.28E-07	3.64E-07
RPS6KA2-IT1	0.390303	0.961023	1.299978	2.77E-06	6.49E-06
AC025871.1	0.012197	0.06596	2.435098	9.41E-06	2.04E-05
AC131009.2	0.007428	0.135601	4.190268	2.85E-10	1.16E-09
AL133410.1	0.327058	0.730955	1.160236	1.18E-07	3.38E-07
AC008731.1	0.18717	0.380254	1.022617	0.008426	0.011722
AC005062.1	0.056893	0.258647	2.184655	1.87E-08	6.01E-08
AC114763.1	0.157054	0.381965	1.282184	2.70E-14	1.96E-13
AC005324.1	0.097216	0.012394	-2.97156	8.30E-26	3.56E-24
AL365436.2	0.093814	0.33851	1.851328	6.85E-10	2.66E-09
AL031186.1	0.482174	1.048917	1.121274	2.47E-08	7.79E-08
AC022211.1	0.22375	1.01338	2.179217	4.19E-17	4.59E-16
ZNF114-AS1	0.028623	0.178181	2.63809	1.63E-05	3.40E-05
CLEC12A-AS1	0.006935	0.027984	2.012669	9.18E-05	0.000172
MNX1-AS2	0.124523	1.100998	3.14433	3.28E-14	2.34E-13
AL031599.1	0.18309	0.03852	-2.24888	9.31E-24	2.55E-22
LINC00106	1.397687	3.0026	1.103172	6.21E-05	0.00012
AL139246.4	0.049493	0.125443	1.34172	0.002532	0.003833
ITGB2-AS1	0.727936	2.520028	1.791556	1.76E-09	6.41E-09
AP000438.1	0.557556	0.074155	-2.91049	1.12E-28	9.96E-27
AC004264.1	0.191211	0.812486	2.087175	5.40E-10	2.12E-09
LINC01341	0.119935	0.298012	1.313114	9.87E-05	0.000184
AC020913.3	0.312274	0.848696	1.442433	0.013852	0.018652
AC006033.2	0.756821	0.333225	-1.18345	1.15E-19	1.77E-18
LINC00365	0.326844	0.136075	-1.2642	1.06E-16	1.08E-15
AC074138.1	0.150652	0.353865	1.231974	7.28E-05	0.000139
HOXD-AS2	0.063293	0.26032	2.040166	2.68E-05	5.41E-05
PRRT3-AS1	0.645466	2.374874	1.879438	6.75E-19	9.51E-18
AC106820.4	0.0866	0.219266	1.340246	0.010224	0.014043
LINC00702	0.85289	0.196636	-2.11684	6.92E-27	3.61E-25
AC011625.1	0.115714	0.0392	-1.56164	1.88E-12	1.05E-11
AP005328.1	0.006258	0.118802	4.246676	3.28E-06	7.57E-06
TMC3-AS1	0.163003	0.350604	1.104947	6.09E-10	2.38E-09

JMJD1C-AS1	0.42719	1.201818	1.492267	2.05E-06	4.92E-06
AL121832.1	0.029359	0.095079	1.695339	2.53E-05	5.13E-05
AL354861.3	0.085938	0.018818	-2.19119	1.78E-25	7.22E-24
AC092171.1	0.08882	0.294679	1.730189	5.09E-05	9.91E-05
AC126696.3	0.007346	0.083252	3.50248	1.36E-11	6.81E-11
LINC01811	0.132424	0.04214	-1.6519	5.84E-14	4.00E-13
Z69733.1	1.065803	0.339055	-1.65235	9.55E-21	1.64E-19
AC012409.3	0.237926	0.118005	-1.01166	4.12E-14	2.89E-13
LINC02363	0.073866	0.158224	1.098997	2.46E-09	8.76E-09
LINC02362	0.155284	1.074704	2.790961	9.24E-19	1.28E-17
BX539320.1	0.702256	1.474796	1.070447	9.06E-05	0.00017
AC020634.1	0.370695	0.178274	-1.05614	1.86E-13	1.17E-12
AC092112.1	0.052448	0.200902	1.937519	0.000306	0.00053
AC112721.1	0.028183	0.228209	3.017467	9.91E-14	6.51E-13
AC012676.4	0.085602	0.184244	1.105908	8.04E-05	0.000152
FLJ16779	0.011875	0.087219	2.87675	1.29E-05	2.75E-05
AL035458.2	0.070378	0.247171	1.812323	9.64E-09	3.20E-08
AC116914.1	0.09075	0.210263	1.212233	0.00109	0.001749
AC087045.2	0.031811	0.162647	2.354163	1.87E-12	1.05E-11
AC127024.5	1.123101	2.507889	1.158986	1.88E-11	9.22E-11
AL360270.1	0.198693	0.461176	1.214777	7.60E-12	3.95E-11
AC092969.1	0.015903	0.29783	4.227164	4.89E-08	1.49E-07
AC010255.1	0.530586	0.132403	-2.00265	5.00E-12	2.65E-11
AC134312.4	0.085815	0.025886	-1.72906	2.64E-08	8.30E-08
CASC9	0.056345	2.727965	5.59739	3.21E-09	1.12E-08
AC100861.1	0.471118	1.403032	1.574388	1.77E-27	1.07E-25
AC087289.5	0.134374	0.348501	1.374905	2.82E-14	2.04E-13
LNCAROD	0.004595	0.369679	6.329996	2.03E-10	8.45E-10
AC105206.1	0.282988	0.084427	-1.74497	2.42E-24	7.55E-23
AL355388.1	2.456694	0.77351	-1.66723	4.47E-16	4.13E-15
AL022068.1	0.696335	0.312568	-1.15561	5.26E-11	2.41E-10
AC073314.1	0.024057	0.05664	1.235348	1.33E-06	3.27E-06
AC245884.9	0.032202	0.076613	1.250452	0.010876	0.014868
AC026780.2	0.075411	0.016161	-2.22229	4.63E-18	5.79E-17
LINC02395	0.002495	0.009097	1.866565	0.002436	0.003697
AC105339.3	0.084265	0.231842	1.460133	5.08E-05	9.90E-05
AC245100.6	0.002814	0.031819	3.499124	6.04E-10	2.36E-09
AL359313.1	0.002721	0.043449	3.996871	2.73E-07	7.39E-07
AL049555.1	0.137584	1.395658	3.342559	1.91E-05	3.96E-05
AC092119.2	0.247906	0.804943	1.699094	1.01E-13	6.61E-13
AC006262.1	0.013584	0.08714	2.681451	8.46E-11	3.75E-10
AL118505.1	0.083232	0.355197	2.093415	8.48E-16	7.57E-15
AL512288.1	0.060168	0.139602	1.214239	0.001242	0.001977
LINC00630	0.33074	0.684354	1.049046	1.17E-15	1.02E-14

LINC01943	0.340074	0.759076	1.158393	1.31E-09	4.85E-09
AC015813.4	0.052298	0.26768	2.355694	2.50E-07	6.81E-07
AC127496.2	0.185983	0.409657	1.139248	0.034316	0.043397
LINC01305	0.001052	0.068658	6.027649	9.84E-09	3.27E-08
AC009275.1	0.096796	0.543884	2.49028	3.46E-10	1.39E-09
AC087392.2	0.501903	0.209951	-1.25736	2.82E-20	4.60E-19
SRD5A3-AS1	0.22532	0.689129	1.612802	4.36E-12	2.33E-11
AP005436.2	0.059021	0.275073	2.220516	0.001288	0.002043
AC093904.2	0.030197	0.379775	3.652653	1.14E-16	1.17E-15
AL157931.1	0.026127	0.376342	3.84841	3.79E-12	2.04E-11
PHKA1-AS1	0.011411	0.069375	2.604026	2.79E-06	6.51E-06
AL512604.2	0.011567	0.040563	1.810129	5.16E-05	0.0001
AC022893.1	0.500038	0.248086	-1.0112	1.53E-18	2.04E-17
AC105345.2	0.018375	0.070115	1.931986	0.018544	0.024457
P4HA2-AS1	0.11889	0.054722	-1.11945	4.23E-11	1.97E-10
AL445465.2	0.040323	0.140463	1.800515	0.004733	0.006858
AC015909.3	0.00442	0.022589	2.353687	0.000422	0.000718
NPSR1-AS1	0.000536	0.06005	6.808287	2.62E-22	5.65E-21
AC012349.1	0.081128	0.020769	-1.96575	2.22E-25	8.79E-24
AC107021.1	0.073782	0.383284	2.377075	0.029476	0.037707
AL021154.1	0.161588	0.460289	1.510217	1.30E-05	2.76E-05
AC002398.2	0.180786	0.036172	-2.32134	3.12E-25	1.17E-23
AL357518.1	0.127831	0.040851	-1.64581	1.27E-12	7.25E-12
SEPT4-AS1	0.146725	0.312267	1.089665	3.39E-13	2.07E-12
LINC01623	0.00509	0.012188	1.259777	0.015037	0.020109
LINC02289	0.566566	0.231331	-1.29228	1.21E-20	2.05E-19
AC131971.1	0.055483	0.685665	3.627398	0.000321	0.000555
AC026202.2	0.391419	0.787987	1.009457	3.90E-05	7.70E-05
LINC01238	0.130278	0.318439	1.289429	2.93E-06	6.81E-06
AL358473.2	0.041151	0.008453	-2.28337	1.73E-12	9.74E-12
AC007881.3	0.075783	0.21193	1.483652	2.80E-07	7.58E-07
LUNAR1	0.024759	0.054208	1.130559	0.003384	0.005023
AL606748.1	0.05158	0.015627	-1.72278	3.92E-10	1.57E-09
LINC00284	0.129371	0.052761	-1.29396	1.20E-14	9.08E-14
AC091153.3	0.095386	0.388328	2.025423	1.98E-14	1.45E-13
AL021807.1	0.310741	0.942532	1.600829	4.88E-05	9.54E-05
INTS9-AS1	0.043676	0.153276	1.811213	0.002602	0.003931
LINC00954	0.080111	0.259527	1.695818	3.47E-08	1.08E-07
PICSAR	1.347209	0.618076	-1.12412	9.25E-20	1.45E-18
AC020765.2	0.319087	0.682894	1.097712	3.12E-05	6.22E-05
AC012617.1	0.052493	0.114196	1.121298	0.00395	0.005798
AC022211.3	0.394957	1.558997	1.980849	6.71E-17	7.10E-16
AC093484.2	0.026195	0.011105	-1.2381	1.11E-10	4.87E-10
AC093151.3	0.007679	0.054871	2.837029	4.43E-09	1.53E-08

MIR497HG	0.519429	0.2139	-1.27999	1.40E-23	3.68E-22
HPN-AS1	0.136546	0.467674	1.776113	6.36E-11	2.87E-10
AC007663.3	0.112946	0.303009	1.423729	2.36E-06	5.57E-06
AL023803.3	0.012577	0.078962	2.650397	1.98E-08	6.36E-08
AC004490.1	0.173346	0.048483	-1.83809	5.17E-22	1.04E-20
AL355472.3	0.095188	0.324462	1.769195	3.64E-07	9.68E-07
AC078852.2	0.041965	0.222226	2.404759	0.00522	0.00752
AC126773.2	0.107354	0.344386	1.681656	8.29E-14	5.52E-13
AP001453.2	0.210506	2.676686	3.668515	1.70E-31	3.53E-29
AL354861.2	0.015921	0.105746	2.731604	1.92E-09	6.96E-09
AL357054.4	1.285339	0.430966	-1.5765	2.57E-23	6.38E-22
FAM83C-AS1	0.0342	0.223504	2.708214	2.73E-15	2.30E-14
AC016205.1	0.019905	0.354742	4.155537	1.43E-22	3.19E-21
AL445307.1	0.821938	0.080624	-3.34974	4.26E-32	1.10E-29
ITGB5-AS1	0.026581	0.167509	2.655752	0.003137	0.004679
AC004832.4	0.087276	0.393212	2.171641	1.51E-05	3.17E-05
AC068594.2	0.093223	0.402669	2.110839	2.22E-05	4.54E-05
BRWD1-AS1	0.019701	0.097888	2.312886	1.82E-07	5.04E-07
CALML3-AS1	0.015971	0.201476	3.657108	7.41E-15	5.82E-14
AL355353.1	1.068577	2.972926	1.476193	1.51E-15	1.31E-14
AL161751.1	0.032535	0.011538	-1.49566	4.00E-14	2.82E-13
AC005037.1	0.067174	0.141582	1.075661	2.51E-07	6.82E-07
AC007163.1	0.012431	0.051315	2.045432	0.000142	0.000258
ELF3-AS1	0.709821	2.605467	1.876015	1.31E-24	4.32E-23
SLC25A25-AS1	0.622049	1.499658	1.269534	7.19E-10	2.78E-09
AC008870.2	0.352037	0.913897	1.376306	9.69E-13	5.64E-12
AC109466.1	0.010551	0.004508	-1.2268	4.30E-17	4.69E-16
LINC01748	0.016818	0.552173	5.037022	1.85E-07	5.15E-07
PTPRG-AS1	0.101523	0.402578	1.987464	2.01E-21	3.80E-20
AC099560.1	0.032561	0.130289	2.000491	0.006894	0.009734
LINC01426	0.284273	1.860514	2.71035	2.45E-23	6.20E-22
SMIM25	22.63802	4.466012	-2.34169	1.94E-30	2.74E-28
LINC02133	0.006689	0.153642	4.521563	1.25E-11	6.26E-11
AC007552.2	0.464346	1.625499	1.80761	0.000291	0.000506
AC117834.1	0.028362	0.165999	2.549123	0.009826	0.013533
AC005329.1	0.010801	0.035363	1.711038	3.81E-05	7.53E-05
AC027544.2	0.062847	0.235244	1.904252	0.002392	0.003634
AL132712.1	0.583233	1.902956	1.706098	1.46E-21	2.82E-20
JARID2-AS1	0.116123	0.354294	1.609294	7.17E-05	0.000137
LINC00943	0.024608	0.10422	2.082428	2.33E-06	5.51E-06
KCNK4-TEX40	0.020885	0.008367	-1.3197	8.28E-08	2.43E-07
AC009139.1	0.007097	0.124268	4.130027	8.23E-09	2.76E-08
AP001189.6	0.096008	0.029667	-1.6943	1.76E-13	1.12E-12
AC078852.1	0.05038	0.245666	2.285771	0.009989	0.01374

TTLL11-IT1	0.020322	0.234333	3.527441	2.98E-11	1.43E-10
AC023024.1	0.120431	0.321804	1.417974	1.80E-10	7.55E-10
AC092354.1	0.315503	0.15326	-1.04168	3.35E-11	1.58E-10
FLJ31356	0.161848	0.432004	1.416404	1.29E-06	3.17E-06
AC010343.3	0.007674	0.176091	4.52028	9.88E-10	3.73E-09
TXNDC12-AS1	0.049352	0.214859	2.122203	0.000173	0.000311
MIR646HG	0.137785	0.400287	1.538619	6.05E-05	0.000117
AC068305.2	0.084128	0.808867	3.265244	7.07E-13	4.16E-12
AC069542.1	0.046817	0.397206	3.084781	0.000669	0.001105
AC078942.1	0.226478	0.076255	-1.57046	4.17E-15	3.40E-14
LINC02043	0.008702	0.057602	2.726656	1.34E-12	7.65E-12
AL139246.1	0.043129	0.136693	1.664226	0.001985	0.003058
AL589765.6	0.070143	0.382365	2.446585	3.48E-15	2.88E-14
AC053503.2	0.025785	0.149689	2.537383	4.26E-17	4.65E-16
AC111149.2	0.076152	1.906014	4.645529	7.60E-19	1.07E-17
AC008083.1	0.03209	0.228222	2.830239	8.22E-12	4.24E-11
CASC15	0.299738	0.863983	1.527303	0.000711	0.001169
AC027117.1	1.839378	4.711463	1.356957	0.008419	0.011718
SNAP47-AS1	0.008034	0.056801	2.821666	9.13E-09	3.04E-08
MIR210HG	1.473172	3.462999	1.233096	1.36E-11	6.79E-11
AC080037.1	0.047743	2.140241	5.486345	7.02E-26	3.03E-24
AC134312.5	0.03668	0.245713	2.743924	2.97E-16	2.84E-15
AC019186.1	0.15036	0.322382	1.100353	2.34E-06	5.52E-06
PTGES2-AS1	0.019387	0.116036	2.581388	8.53E-15	6.59E-14
GHET1	0.093793	0.387263	2.045766	1.55E-24	4.98E-23
LINC01060	0.012857	0.055143	2.100668	0.001634	0.002546
AC067817.2	1.472241	0.511811	-1.52433	2.96E-18	3.79E-17
AL161729.2	0.017505	0.177277	3.340147	0.000249	0.000436
AL158835.2	0.143938	0.47966	1.736568	1.80E-12	1.01E-11
LINC00216	0.171773	0.611601	1.832085	0.002447	0.003712
AP001453.1	0.080358	0.173795	1.112877	8.25E-09	2.76E-08
AP000777.2	0.020457	0.049165	1.265034	0.000112	0.000207
AP000525.1	0.081661	0.382993	2.229604	2.66E-08	8.35E-08
CAPN10-DT	0.269115	0.716702	1.413153	6.53E-17	6.95E-16
AF015262.1	0.002394	0.067157	4.80979	3.23E-10	1.30E-09
DUBR	1.66908	0.785586	-1.08721	1.80E-21	3.41E-20
LINC02595	0.137109	0.527503	1.943856	0.001808	0.002806
TAPT1-AS1	0.59687	1.205861	1.014576	3.65E-10	1.46E-09
LINC01770	1.780861	3.949393	1.149056	2.08E-07	5.75E-07
LINC01781	0.282997	0.719261	1.345731	1.53E-07	4.29E-07
ARHGAP26-AS1	0.023984	0.210502	3.133716	0.001018	0.001641
AC112719.2	0.006996	0.044417	2.666589	0.00029	0.000503
AC092954.1	0.108143	0.043648	-1.30895	4.67E-08	1.42E-07
FMR1-AS1	0.005628	0.023268	2.047569	6.70E-08	1.99E-07

AC034102.8	0.104979	0.317541	1.596837	5.37E-07	1.40E-06
AC018682.1	0.215353	0.547714	1.346718	0.034199	0.043294
AL161725.2	0.100243	0.223153	1.154539	0.0002	0.000356
AC022613.1	0.424128	1.281911	1.595725	9.67E-07	2.43E-06
AC108704.1	0.071972	0.14609	1.021353	0.002685	0.00405
LINC01208	0.003737	0.131126	5.13287	2.75E-16	2.64E-15
AL136115.1	0.110268	0.430183	1.963931	0.003557	0.005267
HOXC-AS2	0.00728	0.31555	5.437781	1.20E-16	1.22E-15
AC113192.2	0.115608	0.02314	-2.32079	3.02E-12	1.64E-11
LINC00857	0.385661	2.075723	2.42821	7.20E-28	4.71E-26
LINC01825	0.014874	0.072936	2.29383	2.84E-05	5.71E-05
SNHG15	4.027224	8.548804	1.085937	1.89E-13	1.19E-12
AP000842.2	0.075303	0.026262	-1.51976	1.21E-12	6.95E-12
AC078960.1	0.052288	0.295288	2.497566	9.48E-08	2.76E-07
AP005899.1	0.412231	1.038111	1.332436	1.05E-05	2.27E-05
AC100793.4	0.836406	0.191162	-2.12941	3.90E-26	1.82E-24
AC021087.3	0.013876	0.192975	3.797784	1.80E-11	8.90E-11
AL445471.1	0.037066	0.247896	2.741569	1.67E-14	1.24E-13
SOX2-OT	0.050326	0.100806	1.002217	0.027107	0.034856
AL161669.3	0.105828	0.274752	1.376406	0.032744	0.04157
GCC2-AS1	0.161448	0.352988	1.128555	2.52E-14	1.84E-13
AL353593.1	0.171857	0.348787	1.021139	1.05E-08	3.49E-08
AL139095.4	0.073901	0.192828	1.383646	8.47E-05	0.00016
LINC01605	0.090494	0.374279	2.048212	9.36E-05	0.000175
AL772337.2	0.304577	0.034446	-3.14438	3.54E-30	4.41E-28
AC024337.2	0.27201	0.091222	-1.5762	6.89E-21	1.21E-19
AC005330.1	0.036824	0.202223	2.457221	5.44E-09	1.86E-08
LINC00607	0.615802	0.263201	-1.2263	1.01E-23	2.75E-22
AC034231.1	0.567567	1.275458	1.168154	9.35E-10	3.55E-09
LINC01852	1.376506	0.655648	-1.07002	3.90E-25	1.42E-23
LINC01569	0.541902	1.675441	1.628438	2.17E-20	3.59E-19
DLEU7-AS1	0.047018	0.22681	2.270193	3.38E-17	3.73E-16
AC092687.3	0.76079	1.885944	1.309718	4.76E-08	1.45E-07
LINC01914	1.203838	0.391426	-1.62083	6.46E-21	1.14E-19
AL121992.3	0.0462	0.241138	2.383906	8.11E-12	4.19E-11
LINC02076	0.010882	0.035462	1.704306	3.27E-06	7.54E-06
AL357093.1	0.700577	0.200296	-1.80641	1.05E-07	3.03E-07
AL158151.1	0.083507	0.211613	1.341453	0.006452	0.009167
AC111152.2	0.684203	0.196848	-1.79734	1.75E-19	2.61E-18
AC021594.1	0.008908	0.113028	3.665379	8.34E-10	3.19E-09
AL513303.1	0.077599	0.015472	-2.32638	2.52E-11	1.22E-10
AC026904.1	0.175618	0.069962	-1.32779	7.04E-10	2.73E-09
AC013553.3	1.019297	0.359712	-1.50266	5.78E-22	1.16E-20
AC105339.2	0.039387	0.155065	1.977071	5.90E-19	8.40E-18

AC124276.1	0.019106	0.084135	2.138702	2.74E-10	1.12E-09
AC007431.1	0.006368	0.103276	4.019632	7.29E-05	0.000139
AL358075.2	0.242945	0.607118	1.321346	0.000106	0.000196
LINC02428	0.005888	0.188158	4.998005	4.94E-09	1.70E-08
AC084876.1	0.129663	0.323178	1.317559	7.43E-08	2.20E-07
LINC00466	0.000581	0.039098	6.073482	1.99E-11	9.73E-11
AC026992.2	0.783404	0.133363	-2.55439	2.19E-27	1.28E-25
BFSP2-AS1	0.017214	0.091607	2.411866	2.80E-09	9.88E-09
LINC02033	0.216879	0.098546	-1.13802	3.24E-14	2.32E-13
AP001972.1	0.722176	0.243357	-1.56928	7.15E-20	1.13E-18
AF131215.6	1.796591	0.652361	-1.46152	3.67E-21	6.76E-20
AC124861.1	0.163007	0.056509	-1.52837	4.12E-14	2.89E-13
LINC01311	0.146309	0.46117	1.656286	1.15E-16	1.17E-15
GFOD1-AS1	0.126468	0.061332	-1.04407	8.60E-17	8.95E-16
AC010201.2	0.27029	0.585871	1.116073	1.37E-05	2.91E-05
AC100821.2	0.087548	0.306645	1.808417	7.53E-09	2.54E-08
LINC00524	0.040262	0.518825	3.687761	9.25E-05	0.000173
AP000842.3	0.005742	0.077138	3.747919	3.67E-07	9.77E-07
CXXC5-AS1	0.023534	0.054293	1.206045	0.021598	0.028233
LINC00412	0.079782	0.2435	1.609778	0.001473	0.00231
AL121956.1	0.011568	0.098487	3.089801	0.005286	0.007613
AC011498.3	0.013519	0.06464	2.257478	6.66E-08	1.98E-07
LINC02026	0.125121	0.255059	1.02751	2.17E-06	5.16E-06
TEX41	0.028795	0.104424	1.858554	0.009331	0.012912
AC068831.6	0.036724	0.087426	1.251315	0.000794	0.001298
AC083900.1	0.288642	0.644662	1.159261	0.008714	0.012103
AL032821.1	0.093105	0.044053	-1.07963	9.87E-05	0.000184
AP000915.1	0.10236	0.031501	-1.7002	8.38E-14	5.56E-13
PKP4-AS1	0.045421	0.290026	2.674742	2.12E-13	1.33E-12
AL031710.2	0.046007	0.270915	2.55792	2.72E-14	1.97E-13
AP001178.1	0.022999	0.126909	2.464132	3.88E-06	8.89E-06
LINC00443	0.185129	0.068137	-1.44202	9.59E-16	8.44E-15
AL590644.1	0.001897	0.084587	5.47828	1.92E-12	1.07E-11
AL121990.1	0.017226	0.048474	1.492618	0.00087	0.001417
AC018695.6	0.088231	0.309885	1.812377	3.69E-05	7.31E-05
SLC2A1-AS1	0.051579	0.302679	2.552945	2.05E-24	6.48E-23
AL358334.2	0.005789	0.020861	1.849381	0.000113	0.000209
AC104461.1	0.140477	1.159962	3.045672	5.89E-09	2.01E-08
AL121790.2	2.337108	0.910687	-1.3597	8.41E-07	2.13E-06
AC239799.2	0.014127	0.032133	1.18564	0.000424	0.000721
AC063948.1	0.259865	0.855081	1.718296	8.85E-14	5.86E-13
AP000424.1	0.010415	0.241094	4.532853	1.01E-18	1.39E-17
HM13-IT1	0.780745	1.633856	1.065358	1.46E-09	5.40E-09
AC244153.1	3.232227	0.611412	-2.40231	1.04E-26	5.28E-25

LINC02056	0.07794	0.0385	-1.01751	5.83E-09	1.99E-08
AL121906.1	0.038107	0.127048	1.73725	1.75E-05	3.63E-05
LINC00355	0.012345	0.313151	4.664837	1.43E-06	3.51E-06
AL136018.1	0.013323	0.102565	2.944514	0.000272	0.000474
DLGAP1-AS2	0.759294	1.553834	1.033102	1.82E-05	3.77E-05

Gene	lncRNA	cor	pvalue	Regulation
GUCY1A2	AC037198.1	0.626216	1.76E-55	postive
PMM2	AC037198.1	0.599993	6.39E-50	postive
PDE5A	AC037198.1	0.668544	1.15E-65	postive
GUCY1A2	AC130650.2	0.727249	6.16E-83	postive
PMM2	AC130650.2	0.623099	8.61E-55	postive
PDE5A	AC130650.2	0.77004	1.13E-98	postive
GUCY1A2	AL109761.1	0.545735	6.46E-40	postive
PMM2	AL109761.1	0.505227	1.48E-33	postive
PDE5A	AL109761.1	0.564211	4.07E-43	postive
GUCY1A2	DLEU1	0.7137	1.45E-78	postive
PMM2	DLEU1	0.592275	2.22E-48	postive
PDE5A	DLEU1	0.748343	2.69E-90	postive
AK1	SFTA1P	0.571431	2.01E-44	postive
PDE8B	AC112722.1	0.538768	9.25E-39	postive
GUCY1A2	AC112722.1	0.600335	5.45E-50	postive
PDE5A	AC112722.1	0.6241	5.19E-55	postive
GUCY1A2	AC092279.1	0.51285	1.09E-34	postive
PMM2	AC092279.1	0.525765	1.13E-36	postive
PDE5A	AC092279.1	0.588918	1.01E-47	postive
CPT1B	LENG8-AS1	0.506361	1.01E-33	postive
GUCY1A2	AC127024.4	0.690649	1.11E-71	postive
PMM2	AC127024.4	0.622274	1.31E-54	postive
PDE5A	AC127024.4	0.740135	2.39E-87	postive
GPX3	AC003092.1	0.886786	######	postive
XDH	AC003092.1	0.544406	1.08E-39	postive
INPP4B	NAALADL2-AS2	0.503086	3.04E-33	postive
AKR1B10	MIR193BHG	0.550907	8.60E-41	postive
GUCY1A2	NFYC-AS1	0.531495	1.40E-37	postive
PDE5A	NFYC-AS1	0.629312	3.59E-56	postive
GUCY1A2	AC108727.1	0.757111	1.42E-93	postive
PMM2	AC108727.1	0.704974	7.01E-76	postive
PDE5A	AC108727.1	0.779013	######	postive
PDE8B	AC027277.2	0.516228	3.36E-35	postive
GUCY1A2	AC027277.2	0.65253	1.28E-61	postive
PMM2	AC027277.2	0.581823	2.32E-46	postive
PDE5A	AC027277.2	0.768891	3.30E-98	postive
INPP4B	AC027277.2	0.565923	2.01E-43	postive
GUCY1A2	AP001469.2	0.553232	3.43E-41	postive
PMM2	AP001469.2	0.522742	3.35E-36	postive
PDE5A	AP001469.2	0.614755	5.50E-53	postive
AK7	LINC01765	0.829009	######	postive
CYP2F1	LINC01765	0.780566	######	postive
PDE5A	AC021851.1	0.573679	7.77E-45	postive

DTYMK	CYTOR	0.567319	1.13E-43	postive
NME1	CYTOR	0.567308	1.13E-43	postive
PDE5A	AC015813.1	0.599861	6.80E-50	postive
GSR	AL033397.1	0.530112	2.32E-37	postive
G6PD	AL033397.1	0.556427	9.61E-42	postive
GCLM	AL033397.1	0.570337	3.19E-44	postive
GUCY1A2	NARF-IT1	0.564476	3.65E-43	postive
PMM2	NARF-IT1	0.596078	3.91E-49	postive
PDE5A	NARF-IT1	0.610618	4.13E-52	postive
AOC3	LINC01936	0.789209	######	postive
FMO2	LINC01936	0.734852	1.65E-85	postive
ADH1B	LINC01936	0.686594	1.54E-70	postive
GSTM5	LINC01936	0.68391	8.62E-70	postive
INMT	LINC01936	0.698302	6.80E-74	postive
PMM2	SAMD12-AS1	0.554536	2.05E-41	postive
PDE5A	SAMD12-AS1	0.547405	3.38E-40	postive
GUCY1A2	AL035409.1	0.549453	1.52E-40	postive
PDE5A	AL035409.1	0.614485	6.28E-53	postive
GPD1	AC026369.3	0.580555	4.04E-46	postive
ALOX5	AC026369.3	0.503321	2.81E-33	postive
ACP5	AC026369.3	0.573169	9.65E-45	postive
GUCY1A2	AC141002.1	0.623634	6.57E-55	postive
PMM2	AC141002.1	0.5601	2.19E-42	postive
PDE5A	AC141002.1	0.655299	2.66E-62	postive
XDH	Z98257.1	0.535228	3.49E-38	postive
GUCY1A2	DLEU2	0.695754	3.77E-73	postive
PMM2	DLEU2	0.644012	1.45E-59	postive
PDE5A	DLEU2	0.761481	2.91E-95	postive
GPD1	PARAL1	0.552404	4.77E-41	postive
ALOX5	PARAL1	0.502693	3.47E-33	postive
HPGDS	PARAL1	0.516248	3.34E-35	postive
GUCY1A2	AL136115.2	0.806659	######	postive
PMM2	AL136115.2	0.710625	1.32E-77	postive
PDE5A	AL136115.2	0.820999	######	postive
PDE5A	AC113139.1	0.536038	2.58E-38	postive
PDE8B	AC093788.1	0.506256	1.05E-33	postive
GUCY1A2	AC093788.1	0.777265	######	postive
PMM2	AC093788.1	0.645972	4.95E-60	postive
PDE5A	AC093788.1	0.795755	######	postive
GUCY1A2	LINC01655	0.533784	5.98E-38	postive
PDE5A	LINC01655	0.542789	2.01E-39	postive
GUCY1A2	AC097641.2	0.502485	3.72E-33	postive
PMM2	AC097641.2	0.522876	3.19E-36	postive
PDE5A	AC097641.2	0.61861	8.19E-54	postive

GUCY1A2	EGOT	0.560764	1.67E-42	postive
PDE5A	EGOT	0.58101	3.31E-46	postive
PDE8B	CR936218.1	0.532028	1.15E-37	postive
GUCY1A2	CR936218.1	0.755371	6.50E-93	postive
PMM2	CR936218.1	0.672652	9.63E-67	postive
PDE5A	CR936218.1	0.80971	######	postive
GUCY1A2	MBNL1-AS1	0.758238	5.24E-94	postive
PMM2	MBNL1-AS1	0.506549	9.46E-34	postive
PDE5A	MBNL1-AS1	0.741486	7.96E-88	postive
GUCY1A2	AP001160.4	0.735262	1.19E-85	postive
PMM2	AP001160.4	0.621615	1.82E-54	postive
PDE5A	AP001160.4	0.752611	7.10E-92	postive
GUCY1A2	AL353804.2	0.760113	9.91E-95	postive
PMM2	AL353804.2	0.558932	3.51E-42	postive
PDE5A	AL353804.2	0.746618	1.15E-89	postive
G6PD	LINC00942	0.526207	9.63E-37	postive
GCLM	LINC00942	0.553449	3.15E-41	postive
GUCY1A2	LANCL1-AS1	0.611124	3.23E-52	postive
ACADL	LANCL1-AS1	0.684109	7.59E-70	postive
PDE5A	LANCL1-AS1	0.589952	6.33E-48	postive
PDE8B	EP300-AS1	0.551952	5.70E-41	postive
GUCY1A2	EP300-AS1	0.732602	9.74E-85	postive
PMM2	EP300-AS1	0.557594	6.02E-42	postive
PDE5A	EP300-AS1	0.733295	5.65E-85	postive
DNMT3B	AC012073.1	0.571178	2.24E-44	postive
CAD	AC012073.1	0.643888	1.55E-59	postive
DNMT3A	AC012073.1	0.643012	2.50E-59	postive
POLE	AC012073.1	0.55295	3.84E-41	postive
AOC3	HSPC324	0.634885	1.95E-57	postive
PTGDS	HSPC324	0.509458	3.51E-34	postive
FMO2	HSPC324	0.607815	1.59E-51	postive
ADH1B	HSPC324	0.601548	3.09E-50	postive
GSTM5	HSPC324	0.575411	3.72E-45	postive
ACADL	HSPC324	0.507287	7.36E-34	postive
CA4	HSPC324	0.764834	1.40E-96	postive
ADCY4	HSPC324	0.623714	6.31E-55	postive
INMT	HSPC324	0.665947	5.44E-65	postive
PDE8B	AC005856.1	0.56862	6.55E-44	postive
GUCY1A2	AC005856.1	0.806369	######	postive
PMM2	AC005856.1	0.574012	6.75E-45	postive
PDE5A	AC005856.1	0.851177	######	postive
INPP4B	AC005856.1	0.571966	1.61E-44	postive
GUCY1A2	AC253576.2	0.691095	8.26E-72	postive
PMM2	AC253576.2	0.609144	8.41E-52	postive

PDE5A	AC253576.2	0.768098	6.90E-98	postive
MAOB	AL031058.1	0.534706	4.25E-38	postive
CPT1B	AL031600.1	0.593295	1.39E-48	postive
PDE8B	AP000692.1	0.50063	6.91E-33	postive
GUCY1A2	AP000692.1	0.67592	1.30E-67	postive
PMM2	AP000692.1	0.600545	4.94E-50	postive
PDE5A	AP000692.1	0.74465	5.90E-89	postive
PMM2	AL606489.1	0.526102	1.00E-36	postive
PTGDS	AL928742.1	0.50839	5.06E-34	postive
GUCY1A2	AL359697.1	0.688382	4.85E-71	postive
PMM2	AL359697.1	0.596769	2.85E-49	postive
PDE5A	AL359697.1	0.738284	1.07E-86	postive
PMM2	AC004596.1	0.540184	5.41E-39	postive
PDE5A	AC004596.1	0.522235	4.02E-36	postive
PMM2	AC084117.1	0.561255	1.37E-42	postive
PDE5A	AC084117.1	0.506702	8.98E-34	postive
AK7	AL121899.1	0.673707	5.05E-67	postive
CYP2F1	AL121899.1	0.666322	4.35E-65	postive
GUCY1A2	ALMS1-IT1	0.586061	3.60E-47	postive
PMM2	ALMS1-IT1	0.528953	3.55E-37	postive
PDE5A	ALMS1-IT1	0.587416	1.97E-47	postive
CPT1B	AC010973.2	0.55215	5.27E-41	postive
PMM2	AL157838.1	0.554741	1.89E-41	postive
PDE5A	AL157838.1	0.556494	9.36E-42	postive
GUCY1A2	AC078778.1	0.588539	1.19E-47	postive
PMM2	AC078778.1	0.593176	1.47E-48	postive
PDE5A	AC078778.1	0.633116	4.94E-57	postive
GUCY1A2	AP002907.1	0.73346	4.96E-85	postive
PMM2	AP002907.1	0.673456	5.89E-67	postive
PDE5A	AP002907.1	0.727655	4.51E-83	postive
PYCR1	MAFG-DT	0.541907	2.81E-39	postive
CAD	AC074117.1	0.520831	6.63E-36	postive
DNMT3A	AC074117.1	0.615126	4.59E-53	postive
PLA2G3	AC016773.1	0.558	5.11E-42	postive
PYCR3	AC138696.2	0.551704	6.28E-41	postive
GUCY1A2	AP000786.1	0.717616	8.41E-80	postive
PMM2	AP000786.1	0.575582	3.45E-45	postive
PDE5A	AP000786.1	0.673996	4.23E-67	postive
CBR3	AL391427.1	0.583952	9.13E-47	postive
CBR1	AL391427.1	0.52295	3.11E-36	postive
UGT2B15	LINC01559	0.503883	2.33E-33	postive
GUCY1A2	AP006621.2	0.515352	4.57E-35	postive
PDE5A	AP006621.2	0.594732	7.24E-49	postive
GUCY1A2	AC133644.2	0.580407	4.31E-46	postive

PMM2	AC133644.2	0.605762	4.24E-51	postive
PDE5A	AC133644.2	0.601336	3.41E-50	postive
AK7	AL357093.2	0.772976	######	postive
CYP2F1	AL357093.2	0.702855	3.04E-75	postive
GUCY1A2	AC099343.2	0.707494	1.21E-76	postive
PMM2	AC099343.2	0.650838	3.32E-61	postive
PDE5A	AC099343.2	0.783626	######	postive
GUCY1A2	ZKSCAN2-DT	0.585483	4.65E-47	postive
PMM2	ZKSCAN2-DT	0.538792	9.17E-39	postive
PDE5A	ZKSCAN2-DT	0.643067	2.43E-59	postive
CPT1B	AL691482.3	0.501648	4.92E-33	postive
CPT1B	AP001160.1	0.500089	8.27E-33	postive
GUCY1A2	AC002128.1	0.578677	9.12E-46	postive
PMM2	AC002128.1	0.509199	3.84E-34	postive
PDE5A	AC002128.1	0.652759	1.13E-61	postive
GUCY1A2	AC103591.3	0.741972	5.35E-88	postive
PMM2	AC103591.3	0.597157	2.38E-49	postive
PDE5A	AC103591.3	0.70845	6.15E-77	postive
CPT1B	ASMTL-AS1	0.522874	3.20E-36	postive
GUCY1A2	AC010186.3	0.800139	######	postive
PMM2	AC010186.3	0.677139	6.09E-68	postive
PDE5A	AC010186.3	0.787197	######	postive
ALOX5	AC090559.1	0.732513	1.04E-84	postive
HK3	AC090559.1	0.657596	7.16E-63	postive
HPGDS	AC090559.1	0.573169	9.65E-45	postive
CPT1B	STAG3L5P-PVRIG2P-PILRB	0.6158	3.29E-53	postive
GUCY1A2	AC090739.1	0.811337	######	postive
PMM2	AC090739.1	0.631138	1.39E-56	postive
PDE5A	AC090739.1	0.822712	######	postive
INPP4B	AC090739.1	0.526661	8.17E-37	postive
GUCY1A2	AP000873.2	0.693875	1.32E-72	postive
PMM2	AP000873.2	0.630021	2.49E-56	postive
PDE5A	AP000873.2	0.736451	4.64E-86	postive
GUCY1A2	AC063965.1	0.748978	1.57E-90	postive
PMM2	AC063965.1	0.690505	1.22E-71	postive
PDE5A	AC063965.1	0.759023	2.61E-94	postive
PDE8B	AC093110.1	0.520236	8.19E-36	postive
GUCY1A2	AC093110.1	0.66872	1.04E-65	postive
ACADL	AC093110.1	0.507385	7.12E-34	postive
PDE5A	AC093110.1	0.636365	8.90E-58	postive
PDE5A	LINC01535	0.514628	5.88E-35	postive
IL4I1	USP30-AS1	0.532581	9.34E-38	postive
PLA2G2D	USP30-AS1	0.545795	6.31E-40	postive

GUCY1A2	AC022150.4	0.666982	2.94E-65	postive
PMM2	AC022150.4	0.624064	5.28E-55	postive
PDE5A	AC022150.4	0.799262	######	postive
LPGAT1	AC022150.4	0.531001	1.67E-37	postive
INPP4B	AC022150.4	0.6066	2.84E-51	postive
CPT1B	AC005726.3	0.516974	2.59E-35	postive
GUCY1A2	AC004884.2	0.673188	6.94E-67	postive
PMM2	AC004884.2	0.636142	1.00E-57	postive
PDE5A	AC004884.2	0.700753	1.29E-74	postive
GUCY1A2	AC079684.1	0.569626	4.30E-44	postive
PMM2	AC079684.1	0.599602	7.67E-50	postive
PDE5A	AC079684.1	0.626281	1.71E-55	postive
GUCY1A2	AF131215.5	0.66745	2.22E-65	postive
PDE5A	AF131215.5	0.716674	1.68E-79	postive
GUCY1A2	AL132780.1	0.58746	1.93E-47	postive
PMM2	AL132780.1	0.544437	1.07E-39	postive
PDE5A	AL132780.1	0.640426	1.01E-58	postive
PDE8B	ANKRD10-IT1	0.541717	3.02E-39	postive
GUCY1A2	ANKRD10-IT1	0.702123	5.03E-75	postive
PMM2	ANKRD10-IT1	0.636123	1.01E-57	postive
PDE5A	ANKRD10-IT1	0.775754	######	postive
CPT1B	AL513320.1	0.535596	3.05E-38	postive
PDE8B	LINC00513	0.527679	5.64E-37	postive
GUCY1A2	LINC00513	0.749266	1.23E-90	postive
PMM2	LINC00513	0.642858	2.72E-59	postive
PDE5A	LINC00513	0.759801	1.31E-94	postive
GUCY1A2	AL590723.1	0.752041	1.16E-91	postive
PMM2	AL590723.1	0.688094	5.85E-71	postive
PDE5A	AL590723.1	0.785339	######	postive
GUCY1A2	AP003170.3	0.636463	8.45E-58	postive
PMM2	AP003170.3	0.561604	1.19E-42	postive
PDE5A	AP003170.3	0.681702	3.50E-69	postive
GUCY1A2	AC008115.3	0.760464	7.24E-95	postive
PMM2	AC008115.3	0.686036	2.21E-70	postive
PDE5A	AC008115.3	0.784577	######	postive
INPP4B	AC008115.3	0.503482	2.66E-33	postive
PDE8B	AC016590.2	0.519285	1.15E-35	postive
GUCY1A2	AC016590.2	0.665417	7.44E-65	postive
PMM2	AC016590.2	0.60927	7.91E-52	postive
PDE5A	AC016590.2	0.791752	######	postive
LPGAT1	AC016590.2	0.508707	4.54E-34	postive
INPP4B	AC016590.2	0.585534	4.54E-47	postive
CPT1B	AL022322.1	0.535123	3.63E-38	postive
TXNRD1	AC089983.1	0.515828	3.87E-35	postive

PDE8B	AC096921.2	0.522977	3.08E-36	postive
GUCY1A2	AC096921.2	0.77799	######	postive
PDE5A	AC096921.2	0.760615	6.33E-95	postive
GUCY1A2	LINC01290	0.648576	1.17E-60	postive
PDE5A	LINC01290	0.654367	4.53E-62	postive
GUCY1A2	AC007546.1	0.800954	######	postive
PMM2	AC007546.1	0.663429	2.41E-64	postive
PDE5A	AC007546.1	0.796484	######	postive
PDE8B	AL109614.1	0.531071	1.63E-37	postive
GUCY1A2	AL109614.1	0.778771	######	postive
PMM2	AL109614.1	0.635418	1.47E-57	postive
PDE5A	AL109614.1	0.817684	######	postive
INPP4B	AL109614.1	0.504861	1.67E-33	postive
GUCY1A2	AP001429.1	0.78415	######	postive
PMM2	AP001429.1	0.681665	3.58E-69	postive
PDE5A	AP001429.1	0.771912	1.93E-99	postive
GUCY1A2	AC004253.1	0.567704	9.60E-44	postive
PDE5A	AC004253.1	0.589845	6.64E-48	postive
AOC3	FENDRR	0.65632	1.49E-62	postive
FMO2	FENDRR	0.623208	8.15E-55	postive
ADH1B	FENDRR	0.652696	1.17E-61	postive
GSTM5	FENDRR	0.510387	2.55E-34	postive
ACADL	FENDRR	0.591453	3.22E-48	postive
CA4	FENDRR	0.827096	######	postive
ADCY4	FENDRR	0.663531	2.27E-64	postive
INMT	FENDRR	0.68685	1.31E-70	postive
CPT1B	AC027796.4	0.529143	3.31E-37	postive
PDE5A	LINC01389	0.583312	1.21E-46	postive
GUCY1A2	AC245014.3	0.809549	######	postive
PMM2	AC245014.3	0.622767	1.02E-54	postive
PDE5A	AC245014.3	0.808334	######	postive
INPP4B	AC245014.3	0.528864	3.66E-37	postive
PDE5A	AC048341.2	0.635028	1.81E-57	postive
PDE5A	AL080317.1	0.538798	9.15E-39	postive
GUCY1A2	AP000866.6	0.755465	5.98E-93	postive
PMM2	AP000866.6	0.591835	2.71E-48	postive
PDE5A	AP000866.6	0.759887	1.21E-94	postive
GUCY1A2	AP001432.1	0.615028	4.81E-53	postive
PMM2	AP001432.1	0.599984	6.42E-50	postive
PDE5A	AP001432.1	0.722109	3.01E-81	postive
GUCY1A2	AL133243.2	0.771312	3.41E-99	postive
PMM2	AL133243.2	0.716417	2.02E-79	postive
PDE5A	AL133243.2	0.801015	######	postive
PRIM1	TMPO-AS1	0.566421	1.64E-43	postive

TYMS	TMPO-AS1	0.51293	1.06E-34	postive
POLE	TMPO-AS1	0.5509	8.62E-41	postive
RRM2	TMPO-AS1	0.591713	2.86E-48	postive
CPT1B	AL928654.2	0.525815	1.11E-36	postive
POLE	AC091057.1	0.601418	3.29E-50	postive
PDE5A	AL132657.1	0.523142	2.90E-36	postive
GUCY1A2	AC139887.4	0.731172	2.98E-84	postive
PMM2	AC139887.4	0.553988	2.54E-41	postive
PDE5A	AC139887.4	0.746983	8.44E-90	postive
AKR1B10	AC129507.4	0.539297	7.58E-39	postive
PLA2G1B	GATA6-AS1	0.523565	2.50E-36	postive
GUCY1A2	SAP30L-AS1	0.789616	######	postive
PMM2	SAP30L-AS1	0.604953	6.22E-51	postive
PDE5A	SAP30L-AS1	0.80276	######	postive
GUCY1A2	AC245884.8	0.591399	3.30E-48	postive
PMM2	AC245884.8	0.515795	3.92E-35	postive
PDE5A	AC245884.8	0.694266	1.02E-72	postive
GUCY1A2	AC138207.4	0.638219	3.31E-58	postive
PMM2	AC138207.4	0.562802	7.27E-43	postive
PDE5A	AC138207.4	0.649178	8.39E-61	postive
GUCY1A2	AC007014.2	0.793379	######	postive
PMM2	AC007014.2	0.692514	3.25E-72	postive
PDE5A	AC007014.2	0.767031	1.85E-97	postive
CPT1B	AC020907.4	0.593115	1.51E-48	postive
AK7	SRGAP3-AS2	0.821555	######	postive
CYP2F1	SRGAP3-AS2	0.861974	######	postive
GUCY1A2	MAGI2-AS3	0.677081	6.32E-68	postive
PDE5A	MAGI2-AS3	0.725244	2.84E-82	postive
GUCY1A2	HIF1A-AS2	0.525144	1.41E-36	postive
PMM2	HIF1A-AS2	0.600709	4.58E-50	postive
PDE5A	HIF1A-AS2	0.572763	1.15E-44	postive
AOC3	TBX5-AS1	0.788938	######	postive
FMO2	TBX5-AS1	0.720779	8.13E-81	postive
ADH1B	TBX5-AS1	0.615159	4.51E-53	postive
PLPP3	TBX5-AS1	0.538687	9.54E-39	postive
GSTM5	TBX5-AS1	0.747634	4.89E-90	postive
PLA2G5	TBX5-AS1	0.538518	1.02E-38	postive
INMT	TBX5-AS1	0.664692	1.14E-64	postive
HK3	LINC02345	0.51664	2.91E-35	postive
HPGDS	LINC02345	0.516163	3.44E-35	postive
PDE2A	AC027031.2	0.533763	6.03E-38	postive
GUCY1A2	AP002336.2	0.76944	1.98E-98	postive
PMM2	AP002336.2	0.68748	8.71E-71	postive
PDE5A	AP002336.2	0.781299	######	postive

GUCY1A2	AP001528.2	0.579925	5.31E-46	postive
PDE5A	AP001528.2	0.571878	1.67E-44	postive
IL4I1	AC004585.1	0.515503	4.34E-35	postive
PLA2G2D	AC004585.1	0.681856	3.18E-69	postive
GUCY1A2	AC010834.3	0.702919	2.91E-75	postive
PMM2	AC010834.3	0.639848	1.38E-58	postive
PDE5A	AC010834.3	0.742917	2.46E-88	postive
CPT1B	AP006284.1	0.533202	7.42E-38	postive
CPT1B	AC087741.1	0.665034	9.34E-65	postive
CPT1B	AC008735.2	0.570643	2.81E-44	postive
GUCY1A2	AL031717.1	0.505282	1.45E-33	postive
PMM2	AL031717.1	0.594768	7.13E-49	postive
PDE5A	AL031717.1	0.578324	1.06E-45	postive
GUCY1A2	AC104695.3	0.684489	5.96E-70	postive
PMM2	AC104695.3	0.655626	2.21E-62	postive
PDE5A	AC104695.3	0.730439	5.27E-84	postive
GUCY1A2	AC083949.1	0.600086	6.12E-50	postive
PMM2	AC083949.1	0.555421	1.44E-41	postive
PDE5A	AC083949.1	0.6812	4.81E-69	postive
PDE8B	Z82243.1	0.526576	8.42E-37	postive
GUCY1A2	Z82243.1	0.710394	1.55E-77	postive
PMM2	Z82243.1	0.667436	2.24E-65	postive
PDE5A	Z82243.1	0.777002	######	postive
INPP4B	Z82243.1	0.542737	2.05E-39	postive
GUCY1A2	AL355488.1	0.59286	1.70E-48	postive
PMM2	AL355488.1	0.517657	2.04E-35	postive
PDE5A	AL355488.1	0.659486	2.40E-63	postive
GUCY1A2	AL662844.3	0.602657	1.84E-50	postive
PMM2	AL662844.3	0.540714	4.43E-39	postive
PDE5A	AL662844.3	0.725849	1.79E-82	postive
PMM2	AC139887.2	0.523522	2.53E-36	postive
PDE5A	AC139887.2	0.578352	1.05E-45	postive
CPT1B	SNHG12	0.53758	1.45E-38	postive
GUCY1A2	AF117829.1	0.670731	3.09E-66	postive
PMM2	AF117829.1	0.716633	1.73E-79	postive
PDE5A	AF117829.1	0.739102	5.52E-87	postive
GUCY1A2	MCM3AP-AS1	0.650672	3.64E-61	postive
PMM2	MCM3AP-AS1	0.58991	6.45E-48	postive
PDE5A	MCM3AP-AS1	0.717503	9.14E-80	postive
GUCY1A2	LINC01376	0.532992	8.02E-38	postive
PMM2	LINC01376	0.513849	7.72E-35	postive
PDE5A	LINC01376	0.578239	1.10E-45	postive
GUCY1A2	AC005021.1	0.650005	5.29E-61	postive
PMM2	AC005021.1	0.62807	6.81E-56	postive

PDE5A	AC005021.1	0.695519	4.41E-73	postive
GUCY1A2	AL022067.1	0.803047	######	postive
PMM2	AL022067.1	0.646216	4.32E-60	postive
PDE5A	AL022067.1	0.765939	5.07E-97	postive
INPP4B	AL022067.1	0.50348	2.67E-33	postive
GUCY1A2	AC010168.2	0.657562	7.30E-63	postive
PMM2	AC010168.2	0.587175	2.19E-47	postive
PDE5A	AC010168.2	0.721935	3.43E-81	postive
GUCY1A2	AP001628.1	0.60183	2.71E-50	postive
PDE5A	AP001628.1	0.674318	3.48E-67	postive
GUCY1A2	AC025171.3	0.677702	4.30E-68	postive
PMM2	AC025171.3	0.526247	9.49E-37	postive
PDE5A	AC025171.3	0.72038	1.09E-80	postive
INPP4B	AC025171.3	0.516778	2.78E-35	postive
GUCY1A2	AC138393.3	0.713194	2.09E-78	postive
PMM2	AC138393.3	0.700046	2.08E-74	postive
PDE5A	AC138393.3	0.737292	2.37E-86	postive
HK3	AC108134.3	0.559846	2.42E-42	postive
CA4	AC108134.3	0.52379	2.30E-36	postive
ADCY4	AC108134.3	0.612834	1.41E-52	postive
PMM2	AC011676.1	0.540253	5.27E-39	postive
PDE5A	AC011676.1	0.544071	1.23E-39	postive
GUCY1A2	AC006017.1	0.727415	5.42E-83	postive
PMM2	AC006017.1	0.662145	5.11E-64	postive
PDE5A	AC006017.1	0.762954	7.71E-96	postive
AK7	AC013264.1	0.702164	4.89E-75	postive
CYP2F1	AC013264.1	0.65049	4.03E-61	postive
PDE8B	AC090579.1	0.508263	5.28E-34	postive
GUCY1A2	AC090579.1	0.734391	2.38E-85	postive
PMM2	AC090579.1	0.628064	6.83E-56	postive
PDE5A	AC090579.1	0.820793	######	postive
INPP4B	AC090579.1	0.577233	1.70E-45	postive
CPT1B	AC011462.4	0.528122	4.80E-37	postive
GUCY1A2	AL354989.1	0.645801	5.43E-60	postive
PMM2	AL354989.1	0.64772	1.89E-60	postive
PDE5A	AL354989.1	0.712323	3.91E-78	postive
CPT1B	AC006435.2	0.564098	4.27E-43	postive
GUCY1A2	AC026356.1	0.678642	2.39E-68	postive
PMM2	AC026356.1	0.625785	2.20E-55	postive
PDE5A	AC026356.1	0.711708	6.07E-78	postive
INPP4B	AC026356.1	0.529537	2.86E-37	postive
CPT1B	AC011498.6	0.558957	3.47E-42	postive
PDE8B	AL513365.2	0.510847	2.18E-34	postive
GUCY1A2	AL513365.2	0.842798	######	postive

PMM2	AL513365.2	0.674052	4.09E-67	postive
PDE5A	AL513365.2	0.838297	######	postive
GUCY1A2	AC025171.4	0.604207	8.85E-51	postive
PDE5A	AC025171.4	0.656183	1.61E-62	postive
GUCY1A2	AL355075.2	0.695952	3.30E-73	postive
PMM2	AL355075.2	0.56031	2.01E-42	postive
PDE5A	AL355075.2	0.716108	2.53E-79	postive
GUCY1A2	AL513327.1	0.615661	3.52E-53	postive
PMM2	AL513327.1	0.613259	1.14E-52	postive
PDE5A	AL513327.1	0.658801	3.57E-63	postive
PFKP	AL731533.2	0.564057	4.34E-43	postive
GUCY1A2	AC139887.1	0.565235	2.67E-43	postive
PMM2	AC139887.1	0.510927	2.12E-34	postive
PDE5A	AC139887.1	0.640405	1.03E-58	postive
PDE8B	AC138932.5	0.509981	2.93E-34	postive
GUCY1A2	AC138932.5	0.797719	######	postive
PMM2	AC138932.5	0.720188	1.26E-80	postive
PDE5A	AC138932.5	0.819885	######	postive
INPP4B	AC138932.5	0.513246	9.51E-35	postive
PDE5A	AL117379.1	0.565919	2.01E-43	postive
ALOX5	LINC01150	0.542435	2.30E-39	postive
HK3	LINC01150	0.536972	1.82E-38	postive
HPGDS	LINC01150	0.574267	6.05E-45	postive
AKR1B10	AP003119.3	0.571069	2.35E-44	postive
GUCY1A2	AC016394.1	0.560515	1.85E-42	postive
PMM2	AC016394.1	0.525626	1.19E-36	postive
PDE5A	AC016394.1	0.640839	8.11E-59	postive
GUCY1A2	AL365277.1	0.756609	2.20E-93	postive
PMM2	AL365277.1	0.642221	3.84E-59	postive
PDE5A	AL365277.1	0.748637	2.10E-90	postive
CPT1B	AP006621.4	0.556039	1.12E-41	postive
PDE8B	AC008669.1	0.579228	7.19E-46	postive
GUCY1A2	AC008669.1	0.675015	2.26E-67	postive
PDE5A	AC008669.1	0.694782	7.21E-73	postive
PRIM1	AC099850.3	0.617142	1.70E-53	postive
PRIM2	AC099850.3	0.564323	3.89E-43	postive
MTHFD2	AC099850.3	0.516687	2.86E-35	postive
POLE2	AC099850.3	0.510289	2.64E-34	postive
PAICS	AC099850.3	0.561522	1.23E-42	postive
CAD	AC099850.3	0.505379	1.41E-33	postive
GART	AC099850.3	0.504366	1.98E-33	postive
TYMS	AC099850.3	0.575981	2.91E-45	postive
POLE	AC099850.3	0.526294	9.33E-37	postive
RRM2	AC099850.3	0.763407	5.11E-96	postive

PPAT	AC099850.3	0.575821	3.12E-45	postive
TK1	AC099850.3	0.570323	3.21E-44	postive
PDE5A	AL158166.1	0.535751	2.87E-38	postive
GUCY1A2	AC011442.1	0.515293	4.67E-35	postive
PMM2	AC011442.1	0.571888	1.66E-44	postive
PDE5A	AC011442.1	0.605131	5.72E-51	postive
PDE8B	AC004908.2	0.50579	1.22E-33	postive
GUCY1A2	AC004908.2	0.694463	8.92E-73	postive
PMM2	AC004908.2	0.551704	6.28E-41	postive
PDE5A	AC004908.2	0.755142	7.93E-93	postive
PDE5A	AC026368.1	0.528319	4.47E-37	postive
GUCY1A2	KDM4A-AS1	0.598676	1.18E-49	postive
PMM2	KDM4A-AS1	0.528059	4.91E-37	postive
PDE5A	KDM4A-AS1	0.660834	1.10E-63	postive
PDE8B	AC007038.2	0.52782	5.36E-37	postive
GUCY1A2	AC007038.2	0.719632	1.90E-80	postive
PMM2	AC007038.2	0.610622	4.12E-52	postive
PDE5A	AC007038.2	0.776398	######	postive
GUCY1A2	AL049869.3	0.765206	9.93E-97	postive
PMM2	AL049869.3	0.685863	2.47E-70	postive
PDE5A	AL049869.3	0.808344	######	postive
GUCY1A2	AP001033.2	0.788337	######	postive
PMM2	AP001033.2	0.682678	1.89E-69	postive
PDE5A	AP001033.2	0.790038	######	postive
AOC3	AC093278.2	0.67804	3.48E-68	postive
FMO2	AC093278.2	0.657082	9.61E-63	postive
ADH1B	AC093278.2	0.655863	1.93E-62	postive
PLPP3	AC093278.2	0.503083	3.05E-33	postive
GSTM5	AC093278.2	0.562764	7.38E-43	postive
ENPP2	AC093278.2	0.509999	2.92E-34	postive
ADCY4	AC093278.2	0.555578	1.35E-41	postive
INMT	AC093278.2	0.633777	3.49E-57	postive
PDE8B	LINC01355	0.514649	5.84E-35	postive
GUCY1A2	LINC01355	0.710967	1.03E-77	postive
PMM2	LINC01355	0.610068	5.39E-52	postive
PDE5A	LINC01355	0.765383	8.44E-97	postive
PDE5A	AC011815.1	0.549856	1.30E-40	postive
AOC3	AP001189.3	0.695224	5.37E-73	postive
FMO2	AP001189.3	0.5147	5.74E-35	postive
ADCY4	AP001189.3	0.500823	6.48E-33	postive
GUCY1A2	AC124319.1	0.541023	3.94E-39	postive
PMM2	AC124319.1	0.600387	5.32E-50	postive
PDE5A	AC124319.1	0.580671	3.84E-46	postive
GUCY1A2	SCAANT1	0.533631	6.33E-38	postive

PMM2	SCAANT1	0.521192	5.83E-36	postive
PDE5A	SCAANT1	0.622648	1.08E-54	postive
GOT1	AC083809.1	0.610318	4.77E-52	postive
AK7	AL645924.1	0.795052	######	postive
CYP2F1	AL645924.1	0.715102	5.27E-79	postive
CA4	TBX2-AS1	0.507905	5.97E-34	postive
ADCY4	TBX2-AS1	0.516254	3.34E-35	postive
GUCY1A2	AL031667.3	0.526416	8.93E-37	postive
PMM2	AL031667.3	0.555099	1.63E-41	postive
PDE5A	AL031667.3	0.525285	1.34E-36	postive
CPT1B	AC006042.1	0.556318	1.00E-41	postive
GUCY1A2	AC090517.2	0.552362	4.85E-41	postive
PDE5A	AC090517.2	0.629285	3.64E-56	postive
GUCY1A2	AC079907.1	0.524149	2.02E-36	postive
PDE5A	AC079907.1	0.576824	2.03E-45	postive
GUCY1A2	AC073534.1	0.570683	2.76E-44	postive
PDE5A	AC073534.1	0.664965	9.73E-65	postive
PDE8B	LINC00894	0.503446	2.70E-33	postive
GUCY1A2	LINC00894	0.646893	2.98E-60	postive
PMM2	LINC00894	0.568218	7.75E-44	postive
PDE5A	LINC00894	0.741797	6.18E-88	postive
PMM2	AC007991.2	0.552465	4.65E-41	postive
PDE5A	AC007991.2	0.556005	1.14E-41	postive
GSR	AL365181.3	0.502881	3.26E-33	postive
GUCY1A2	AC026355.1	0.654104	5.26E-62	postive
PMM2	AC026355.1	0.543648	1.44E-39	postive
PDE5A	AC026355.1	0.680369	8.11E-69	postive
GUCY1A2	RPS6KA2-IT1	0.520437	7.62E-36	postive
PMM2	RPS6KA2-IT1	0.508947	4.18E-34	postive
PDE5A	RPS6KA2-IT1	0.514898	5.36E-35	postive
CPT1B	AL031186.1	0.535171	3.57E-38	postive
GUCY1A2	AC022211.1	0.55512	1.62E-41	postive
PMM2	AC022211.1	0.629419	3.39E-56	postive
PDE5A	AC022211.1	0.596795	2.81E-49	postive
GUCY1A2	AC020913.3	0.741081	1.11E-87	postive
PMM2	AC020913.3	0.709502	2.92E-77	postive
PDE5A	AC020913.3	0.758569	3.91E-94	postive
GUCY1A2	AC127024.5	0.638236	3.29E-58	postive
PMM2	AC127024.5	0.537246	1.64E-38	postive
PDE5A	AC127024.5	0.687876	6.74E-71	postive
GUCY1A2	LINC00630	0.740628	1.60E-87	postive
PMM2	LINC00630	0.638499	2.85E-58	postive
PDE5A	LINC00630	0.789255	######	postive
INPP4B	LINC00630	0.524011	2.13E-36	postive

TXNRD1	AC009275.1	0.534142	5.24E-38	postive
GCLM	AC009275.1	0.504691	1.77E-33	postive
GPX2	AC009275.1	0.523304	2.74E-36	postive
GUCY1A2	AC131971.1	0.794199	######	postive
PMM2	AC131971.1	0.70573	4.14E-76	postive
PDE5A	AC131971.1	0.78446	######	postive
INPP4B	AC131971.1	0.52465	1.69E-36	postive
GUCY1A2	AC026202.2	0.733592	4.47E-85	postive
PMM2	AC026202.2	0.633043	5.14E-57	postive
PDE5A	AC026202.2	0.772927	######	postive
ENTPD8	SLC25A25-AS1	0.515728	4.01E-35	postive
GUCY1A2	AC008870.2	0.560808	1.64E-42	postive
PMM2	AC008870.2	0.556383	9.78E-42	postive
PDE5A	AC008870.2	0.583875	9.44E-47	postive
GPD1	SMIM25	0.531981	1.17E-37	postive
ALOX5	SMIM25	0.647261	2.43E-60	postive
HK3	SMIM25	0.732404	1.14E-84	postive
ACP5	SMIM25	0.729964	7.61E-84	postive
GUCY1A2	AC007552.2	0.679974	1.04E-68	postive
PMM2	AC007552.2	0.517669	2.03E-35	postive
PDE5A	AC007552.2	0.76645	3.17E-97	postive
RDH10	AC111149.2	0.528678	3.92E-37	postive
PDE8B	AC067817.2	0.509728	3.20E-34	postive
GUCY1A2	AC067817.2	0.759327	2.00E-94	postive
PMM2	AC067817.2	0.517374	2.25E-35	postive
PDE5A	AC067817.2	0.709286	3.41E-77	postive
PDE8B	LINC00216	0.511819	1.56E-34	postive
GUCY1A2	LINC00216	0.815292	######	postive
PMM2	LINC00216	0.692442	3.41E-72	postive
PDE5A	LINC00216	0.842598	######	postive
INPP4B	LINC00216	0.50743	7.02E-34	postive
GUCY1A2	DUBR	0.505567	1.32E-33	postive
PDE5A	DUBR	0.607544	1.81E-51	postive
PDE5A	TAPT1-AS1	0.513685	8.17E-35	postive
PTGDS	LINC01781	0.627537	8.96E-56	postive
GUCY1A2	AC018682.1	0.624168	5.01E-55	postive
PMM2	AC018682.1	0.649937	5.49E-61	postive
PDE5A	AC018682.1	0.643003	2.51E-59	postive
GUCY1A2	AP005899.1	0.617705	1.28E-53	postive
PMM2	AP005899.1	0.628387	5.79E-56	postive
PDE5A	AP005899.1	0.690094	1.59E-71	postive
GUCY1A2	AF131215.6	0.551421	7.03E-41	postive
PDE5A	AF131215.6	0.583244	1.25E-46	postive
PDE8B	AC010201.2	0.526224	9.57E-37	postive

GUCY1A2	AC010201.2	0.706087	3.23E-76	postive
PMM2	AC010201.2	0.529482	2.92E-37	postive
PDE5A	AC010201.2	0.773367	######	postive

Supplementary materials Table Table 4

lncRNA	Coef
MIR193BHG	0.113247
AC245595.1	0.193863
AC084859.1	-0.20993
MYOSLID	0.033207
MIR22HG	-0.05226
HSPC324	-0.15418
LINP1	0.010916
AL034397.3	-0.11026
AC102953.2	0.12467
LINC01703	0.191701
AC084117.1	0.149958
