# Supplementary material for: Construction of 11 metabolic-related lncRNAs to predict the prognosis in lung adenocarcinoma
Source: BMC Med Genomics. 2023 Dec 18;16:330. doi: 10.1186/s12920-023-01764-9 (PMC10726503; doi:10.1186/s12920-023-01764-9)
Supplement: Supplementary file 2 — Supplementary Material 2 [file 12920_2023_1764_MOESM2_ESM.docx]

**Supporting materials**

Supplementary materials Table 1: 253 differentially expressed metabolism-related genes were identified, of which 78 genes were down-regulated and 175 genes were up-regulated.

Supplementary materials Table 2: 1995 differentially expressed lncRNAs, of which 650 lncRNAs were down-regulated and 2345 lncRNAs were up-regulated.

Supplementary materials Table 3: 233 metabolic-related lncRNAs through Pearson correlation analysis, the standards were set as | R2 |>0.5 and p<0.05.

Supplementary materials Table 4: 11 core lncRNAs closely related to prognosis through LASSO regression analysis.
